# ZFP64 Promotes Gallbladder Cancer Progression through Recruiting HDAC1 to Activate NOTCH1 Signaling Pathway

**DOI:** 10.3390/cancers15184508

**Published:** 2023-09-11

**Authors:** Zhiqiang He, Yuhan Zhong, Haijie Hu, Fuyu Li

**Affiliations:** 1 Department of Biliary Surgery, West China Hospital, Sichuan University, Chengdu 610041, China; hzq_97@hotmail.com; 2 Laboratory of Liver Transplantation, Key Laboratory of Transplant Engineering and Immunology, National Health Commission (NHC), West China Hospital, Sichuan University, Chengdu 610041, China; hanzi19971122@163.com

**Keywords:** gallbladder cancer, ZFP64, Notch1, NUMB, HDAC1

## Abstract

**Simple Summary:**

Gallbladder cancer (GBC) is one of the solid tumors with the worst prognosis, and existing treatments for GBC are not effective. Understanding the disease process of GBC from the perspective of molecular mechanisms is helpful in developing new therapies. In this study, we aimed at exploring a meaningful regulator in GBC. Through a series of in vitro and in vivo assays, we confirmed that zinc finger protein 64 (ZFP64) is a potential promoter and ZFP64-Notch1-HDAC1 is an essential oncogenic axis in GBC progression. Targeting ZFP64 may pave the way for the development of effective therapy for this disease.

**Abstract:**

The lack of meaningful and effective early-stage markers remains the major challenge in the diagnosis of gallbladder cancer (GBC) and a huge barrier to timely treatment. Zinc finger protein 64 (ZFP64), a member of the zinc finger protein family, is considered to be a promising predictor in multiple tumors, but its potential effect in GBC still remains unclear. Here, we identified that ZFP64 was a vital regulatory protein in GBC. We found that ZFP64 expressed higher in GBC gallbladder carcinoma tissues than in normal tissues and was positively correlated with poor prognosis. Furthermore, ZFP64 was responsible for the migration, invasion, proliferation, anti-apoptosis, and epithelial mesenchymal transition (EMT) of GBC cells in vitro and in vivo. Mechanistically, through Co-IP assay, we confirmed that ZFP64 recruits HDAC1 localized to the promoter region of *NUMB* for deacetylation and therefore inhibits *NUMB* expression. The downregulation of NUMB enhanced the activation of the Notch1 signaling pathway, which is indispensable for the GBC-promotion effect of ZFP64 on GBC. In conclusion, ZFP64 regulated GBC progression and metastasis through upregulating the Notch1 signaling pathway, and thus ZFP64 is expected to become a new focus for a GBC prognostic marker and targeted therapy.

## 1. Introduction

Gallbladder cancer (GBC) is one of the six major gastrointestinal malignancies and is a common solid tumor in malignant biliary tract cancer (BTC) [1]. In recent years, the incidence of GBC has continuously increased in several countries, particularly in Southeast Asia [2]. Due to the lack of characteristic early symptoms and tumor markers, GBC patients are often diagnosed at an advanced stage, with a poor prognosis and high mortality rate [3]. The pathogenesis of GBC is extremely complex, and most GBC patients also have concomitant gallbladder inflammation and gallstones, leading to the uncontrolled and rapid growth of GBC under this multi-disease setting [2]. Furthermore, common treatments such as surgical resection and chemotherapy are ineffective in the management of advanced GBC [4]. As a result, it is of great therapeutic importance to discover novel targeted therapies for GBC and to prolong the survival time of GBC patients.

Transcriptional dysregulation is a crucial feature of tumor cells and transcription factors have a direct impact on this process [5]. Zinc finger proteins, as the largest family of transcription factors, have been shown to regulate the development of multiple cancers, including gallbladder cancer [6,7]. Zinc finger protein 64 (ZFP64) is a member of the zinc-finger type C2H2 transcription factors and, like other members of this family, the predominant function of ZFP64 is thought to regulate transcription [8]. Early studies identified ZFP64 as a coactivator of Notch1. ZFP64 interacts with the Notch intracellular domain (NICD) and participates in the regulation of Notch1 signaling [9]. In most instances, ZFP64 functions as a tumorigenesis promoter. ZFP64 was significantly upregulated in various types of human cancers, including gastric cancer (GC), hepatocellular carcinoma (HCC), and oesophageal cancer [10,11]. As a transcription factor, ZFP64 also activates the promoter of the mixed spectrum leukaemia (MLL) gene, thereby maintaining this oncogene at a high expression level [12]. In addition, by binding to and increasing the expression of promoters such as Gal-1, PD-1, and CTLA-4, ZFP64 enables cancer cells to acquire stemness and resist immunosuppression; ZFP64 possesses the capability to affect tumor microenvironment and renders tumor cells resistant to immunosuppression [10,13]. These findings are an indication that the targeting of ZFP64 may be a potential cancer therapy.

Notch is a family of receptors that is widely distributed and highly conserved in multicellular organisms. In mammals, the Notch family consists of four receptors, Notch1-4, which can influence organ development and repair organismal damage by regulating cell proliferation and differentiation [14]. Notch1 has complex regulatory functions in cancer. On the one hand, Notch1 has been reported to promote cancer cell migration and invasion and inhibit apoptosis in many tumors, including tongue cancer [15], pancreatic cancer [16], and breast cancer [17], while mechanistic studies in bladder cancer have revealed that Notch1 also serves as an oncogene [18]. The Notch1 signaling pathway can be suppressed by its negative regulator NUMB through several mechanisms, such as Notch1 ubiquitination, Notch1 endocytosis, and degradation of the NICD [19,20]. Considering the possible roles of Notch1 in cancer, NUMB is promised to exhibit the inhibitory potential for tumor progression and has been clarified in lung cancer and pancreatic cancer. Another study found that high Notch1 expression was closely related to poor prognosis in GBC patients [21], but the exact mechanism of Notch1’s regulatory role in GBC has not been fully illustrated.

Here, through in vivo and in vitro assays, we confirmed that ZFP64 was an unfavorable prognostic factor in GBC and that ZFP64 promoted GBC development by supporting malignant biological behaviors such as GBC cell proliferation, anti-apoptosis, migration, invasion, and epithelial mesenchymal transition (EMT). Furthermore, we reported the Notch1 signaling pathway as a downstream mechanism of ZFP64 and further confirmed that ZFP64 activated the Notch1 signaling pathway by recruiting HDAC1 to the *NUMB* promoter region and modifying H4K77 and H3K18 through deacetylation and delactylation, respectively. Our findings provided insights into the concrete mechanisms of GBC and suggested that targeting ZFP64 promised to be an efficient therapeutic option for GBC.

## 2. Materials and Methods

### 2.1. Human Specimens and Cell Culture

Human GBC tissues and adjacent normal tissues were acquired from the Department of Biliary Surgery of the Western China Hospital of Sichuan university with informed consent of the patients and approval of the institutional research ethics committee. Fresh tissues were stored at −80 °C for subsequent research or embedded with paraffin. Corresponding clinicopathological features including age, sex, liver function index, tumor marker, and lymph node metastasis were collected by clinical follow-up. None of the patients received radiotherapy, chemotherapy, and any other adjuvant treatment before surgery.

GBC-SD and NOZ cell line were purchased from Shanghai Key Laboratory of Biliary Disease Research and cultured in Dulbecco’s Modified Eagle’s medium (Hyclone, Logan, UT, USA) supplemented with 10% FBS (Gibco, Carlsbad, CA, USA) and 100 unit/mL penicillin and 100 μg/mL streptomycin.

### 2.2. Construction of Stable Cell Lines

Lentivirus for overexpression and knockdown of ZFP64 was purchased from Shanghai Genechem Co., Ltd. (Shanghai, China) GBC-SD or NOZ cells were implanted into the six-well plate, and when the cells grew to 70% full, lentivirus was added and infected for 24 h. Tumor cells with ZFP64 overexpression or knockdown were subsequently screened using complete culture medium supplemented with 10 μg/mL puromycin.

### 2.3. Reverse Transcription and Quantitative Real-Time PCR (RT-qPCR)

Total RNAs of cells and tissues were extracted using Trizol (Invitrogen, Carlsbad, CA, USA) and then reversely transcribed with SuperScript TMII reverse transcriptase (Vazyme, Nanjing, China). Quantitative real-time PCR was performed with SYBR Green qPCR Master Mix (Vazyme) on Bio-Rad CFX96TM (Hercules, CA, USA) using the 2^−∆∆Ct^ method.

Primer pairs:

- ZFP64 forward: GATGCCTTTGTAGCTCACAAGC
- ZFP64 reverse: GTCTGGGTCTCCGAGGTGAT
- NUMB forward: GCAGCAGACATTCCCTCACT
- NUMB reverse: AGAACCGTTGAGGTGCTGAG

### 2.4. Western Blot

Tissues or cells were splatted on ice for 40 min with RIPA supplemented with 1% protease inhibitor and 1% phosphatase inhibitor, then the protein was quantified using a BCA kit (Epizyme, Shanghai, China). After denaturation, the proteins with different weights were separated via 10% SDS-PAGE and then transferred onto a PVDF membrane (Merck, Darmstadt, Germany). The membrane was blocked with 5% skim milk dissolved in TBST at RT for 1 h, incubated in specific primary antibody solution at 4 °C overnight, and then incubated in corresponding second antibody solution at RT for 1 h. The results were detected using the enhanced chemiluminescence (Epizyme, Shanghai, China). The details of all primary antibodies are listed in Table S3.

### 2.5. Immunofluorescence Staining

The cell slipper was placed into a 24-well plate. GBC-SD and NOZ cells stably overexpressing or knocking down ZFP64 were seeded into a 24-well plate and incubated overnight. Cells were fixed with 4% paraformaldehyde and then permeabilized with 0.5% TritonX-100. Next, cells were incubated in NICD primary antibody at 4 °C overnight and then incubated in second antibody conjugated with FITC. The nucleus was stained with DAPI solution and the fluorescence intensity of each group of cells was digitized using Image J (v2021.8.0, National Institutes of Health, Bethesda, MD, USA).

### 2.6. Cell Migration and Invasion Assay

3 × 10^4^ or 5 × 10^4^ cells were resuspended in 200 μL serum-free DMEM and then seeded into the transwell upper chamber with or without Matrigel (Corning, New York, NY, USA). The transwell lower chamber was filled with 600 μL medium with 10% FBS and incubated for 24 h. Cells on the inside of chamber were gently removed with cotton swabs, and cells on the outside of chamber were fixed with 4% paraformaldehyde and then stained using 1% crystal violet. Five random fields were photographed under the microscope and the average number of cells in the five fields was calculated using Image J.

### 2.7. Cell Proliferation Assay (EdU)

The cell proliferation ability was examined using a EdU kit (KeyGEN BioTECH, Nanjing, China) according to the instructions. GBC-SD and NOZ cells were seeded into a 96-well plate and cultured to a suitable density. An amount of 10 μM EdU solution was added into cells and then the cells were incubated for 2 h. Next, the cells were fixed with 4% paraformaldehyde and permeabilized with 0.05% TritonX-100. Cells labeled with EdU developed color by the addition of a Click-iT reaction mixture. The nuclei were stained with 1 × Hoechst33342 solution for 10 min. Five fields were randomly photographed under an inverted fluorescence microscope and the proportion of EdU-positive cells to total cells was calculated using Image J.

### 2.8. Chromatin Immunoprecipitation (ChIP)-qPCR Assay

ChIP assay was performed used a ChIP kit (Bersin BioTM, Guangzhou, China). Following the instructions, the cells were crosslinked with 1% formaldehyde and then DNA were broken into 100–500 bp lengths using non-contact ultrasound, followed by the addition of anti-ZFP64, anti-H3K18lac, or anti-H4K77ac antibodies and incubated at 4 °C for 12 to 16 h. An amount of 20 µL protein A/G-beads were added and incubated at room temperature for 30 min, and then the DNA fragments were collected using elution buffers. Three primers were designed according to the binding sites (BS) of ZFP64 in the NUMB promoter region predicted by JASPAR (http://jaspar.genereg.net/, accessed on 22 February 2023). Quantitative PCR was used to detect the target DNA fragment (Table 1).

### 2.9. Co-Immunoprecipitation (Co-IP) Assay

PierceTM Co-IP kit (Thermo SCIENTIFIC, Carlsbad, CA, USA) was used to confirm the interaction between ZFP64 with HDAC1. Briefly, ZFP64 or HDAC1 antibody were crosslinked with magnetic beads. Tumor cells were lysed with cell lysate, then centrifuged at 12,000× *g* for 5 min to remove the precipitation and finally total protein of the cells was obtained. The total protein was incubated with magnetic bead-conjugated antibodies at RT for 1 h. Antigens coupled to magnetic beads were eluted and analyzed using mass spectrometry and Western blot.

### 2.10. Mouse Subcutaneous Xenograft Model

Subcutaneous xenograft assay had required approval of the Animal Ethics Committees of Western China Hospital of Sichuan university. Five-week-old BALB/C nude male mice were purchased from Beijing HFK Bioscience Co., Ltd. (Beijing, China) and kept in a sterile, pathogen-free environment. Then, 2 × 10^6^ GBC-SD or NOZ cells in the logarithmic growth phase were injected into the right underarm of the mice. Each group consisted of 6 mice. The mice were sacrificed, and the tumors were removed and then weighed and measured after 20 or 28 days.

### 2.11. Statistical Analysis

All statistics in this study were analyzed using unpaired Student’s *t* test, one-way ANOVA, two-way ANOVA, or Kaplan–Meier survival analysis. *p* values less than 0.05 were considered statistically significant. All the statistics in this paper were conducted by GraphPad Prism 8.0.

## 3. Results

### 3.1. ZFP64 Is Overexpressed in GBC Patients and Associated with Poor Prognosis

**Figure 1:**
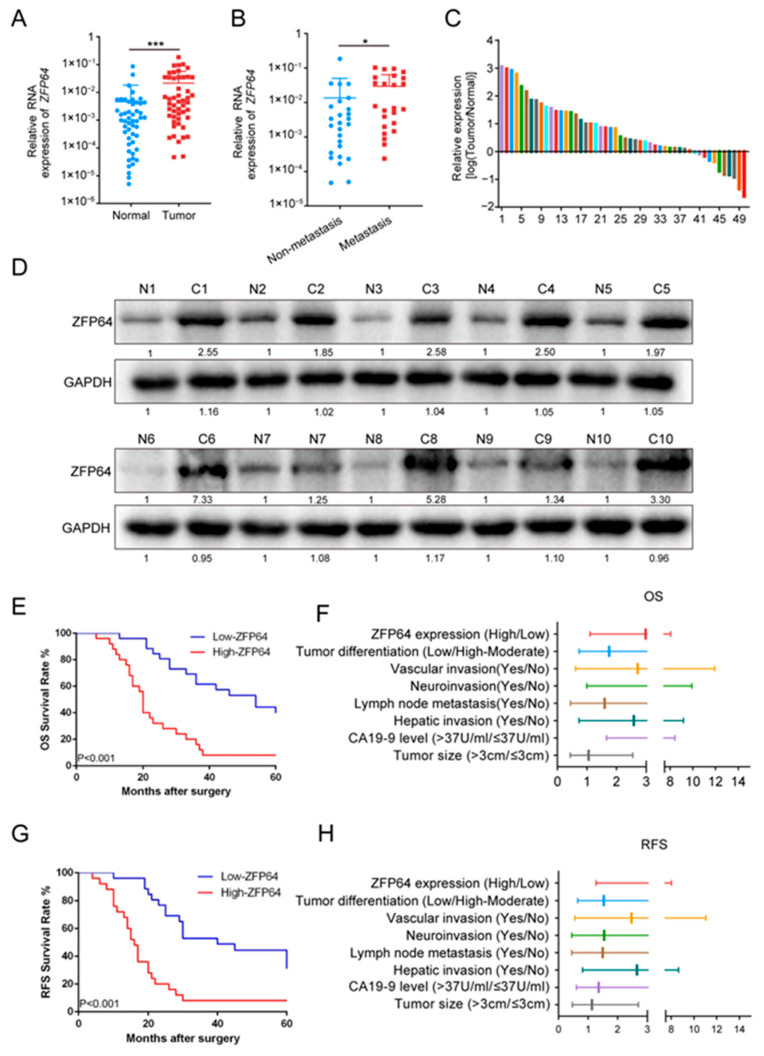
ZFP64 is highly expressed in GBC patients and the higher expression of ZFP64 is associated with poor prognosis. (**A**) The mRNA level of ZFP64 in gallbladder carcinoma tissues and in paired adjacent normal tissues from 50 GBC patients. (**B**) The mRNA level of ZFP64 in metastatic (*n* = 24) and non-metastatic (*n* = 26) gallbladder carcinoma tissues. (**C**) The ratio of ZFP64 mRNA level in gallbladder carcinoma tissues and in paired adjacent normal tissues. Each color represented an individual patient. (**D**) Western blot assays were performed to detect the expression level of ZFP64 in gallbladder carcinoma tissues and in adjacent normal tissues from 10 GBC patients. (N: Normal gallbladder tissues; C: gallbladder carcinoma tissues). (**E**) The relationship between the overall survival (OS) with the expression level of ZFP64 (*n* = 50). (**F**) Prognostic indicators for OS in multivariate analysis (**G**) The relationship between the recurrence-free survival (RFS) with the expression level of ZFP64 (*n* = 50). (**H**) Prognostic indicators for RFS in multivariate analysis. Each color in Figure 1F/1H represented different clinical pathological features as Y-axis shows. All data were assessed using unpaired Student’s *t* test or Kaplan–Meier survival analysis, as mean ± SD, * *p* < 0.05, *** *p* < 0.001. See Figure S2 for original western blots.

As mentioned before, ZFP64 has been implicated in promoting multiple cancers. To clarify the possible role of ZFP64 in GBC, we evaluated and compared the expression difference of ZFP64 between GBC tissues and adjacent normal tissues from 50 GBC patients. Indeed, the RT-qPCR results showed that *ZFP64* mRNA levels were evidently much higher in gallbladder carcinoma tissues than in adjacent normal tissues from GBC patients (Figure 1A,C). In 50 gallbladder carcinoma tissues, the mRNA levels of *ZFP64* in metastatic gallbladder carcinoma tissues were higher than that in non-metastatic gallbladder carcinoma tissues (Figure 1B). Similarly, Western blot analysis showed the analogous results in protein levels: the expression of ZFP64 was significantly higher in gallbladder carcinoma tissues than in adjacent normal tissues in all 10 GBC patient samples (Figure 1D). Overall, ZFP64 is highly expressed in GBC tissues, especially in metastatic GBC tissues.

To further investigate the relationship between the clinicopathological features of GBC patients and ZFP64 expression, we divided 50 GBC patients with detailed clinicopathological information into a high ZFP64 expression group (*n* = 25) and a low ZFP64 expression group (*n* = 25), and then analyzed their pathological features. The analysis revealed that patients with high expression of ZFP64 had higher CA19-9 levels and higher rates of lymph node metastasis, liver invasion, nerve invasion, and lower tumor differentiation (Table 2). These aggressive characteristics may directly contribute to the poor prognosis in these GBC patients. Subsequently, we went on to look at whether ZFP64 had an impact on patient survival. We found that patients with high ZFP64 expression had shorter overall survival (OS) and recurrence-free survival (RFS) compared with the low ZFP64 expression group (Tables S1 and S2; Figure 1E,G). In both univariate and multivariate Cox proportional hazards regression models, the expression level of ZFP64 was an independent prognostic factor for OS and RFS (Tables S1 and S2, Figure 1F,H). Taken together, these results suggest that ZFP64 is a potential predictive biomarker for GBC and is strongly associated with GBC prognosis and metastasis.

### 3.2. ZFP64 Promotes Gallbladder Cancer Progression In Vitro and In Vivo

**Figure 2:**
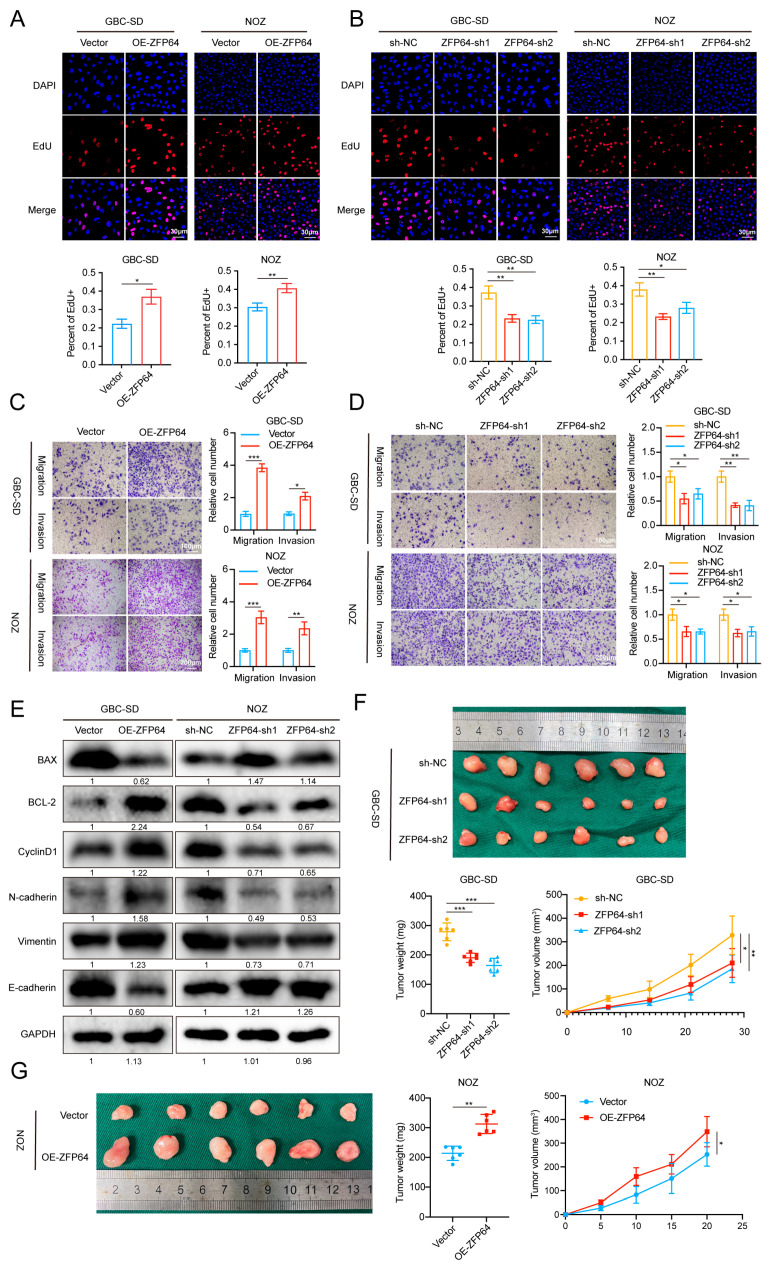
ZFP64 promotes GBC cells in vitro and in vivo invasion, proliferation, EMT, and migration. (**A**,**B**) Transwell assays were conducted to detect the migration and invasion ability of GBC cells overexpressing ZFP64 (**A**) or knocking down ZFP64 (**B**) compared with control group, respectively. (**C**,**D**) EdU assays were used to determine the proliferation ability of GBC cells overexpressing ZFP64 (**C**) or knocking down ZFP64 (**D**). (**E**) Western blot analyzed the expression level of proteins related to apoptosis, proliferation, and EMT. (**F**,**G**) Tumors from ZFP64 knockdown group (**F**), ZFP64 overexpressing group (**G**), and control group were dissected from BALB/C nude mice after transplantation, and the weight and volume were measured. (*n* = 6, 2 × 10^6^ cells per mouse). All data were assessed using unpaired Student’s *t* test, one-way ANOVA, or two-way ANOVA, as mean ± SD, * *p* < 0.05, ** *p* < 0.01, *** *p* < 0.001. See Figure S3 for original western blots.

To further elucidate the specific biological functions of ZFP64 in GBC progression, we generated GBC-SD and NOZ cell lines with stable overexpression or the knockdown of ZFP64 via lentivirus-mediated infection. The RT-qPCR and Western blot results demonstrated that ZFP64 was successfully overexpressed or knocked down at both the mRNA and protein level in both cell lines (Figure S1). The 5-ethynyl-20-deoxyuridine (EdU) assays demonstrated that the overexpression of ZFP64 significantly enhanced GBC cells proliferation and ZFP64 knockdown leading to opposing results (Figure 2A,B). Simultaneously, a transwell assay showed that the migration and invasion ability of tumor cells was significantly enhanced in the overexpressed ZFP64 group, while it was significantly decreased after ZFP64 was knocked down (Figure 2C,D). Western blot also showed a dramatic increase in BCL-2, cyclinD1, N-cadherin, and vimentin expression with ZFP64 overexpression, and a downregulation in BAX and E-cadherin, while ZFP64 knockdown exhibited opposing results (Figure 2E). All the results above meant that ZFP64 had a promotive effect on GBC cell anti-apoptosis, proliferation, migration, invasion, and EMT, thereby contributing to the procession of GBC tumors.

As our results above have revealed that ZFP64 promotes GBC cell progression in vitro, it is necessary to further verify whether ZFP64 has the same promotive effect on GBC cell in vivo through subcutaneous tumorigenesis assays. GBC-SD and NOZ cells stably overexpressing or knocking down ZFP64 were injected into BALB/C nude male mice. In terms of tumor growth rate and final tumor weight, the ZFP64 overexpression group showed the opposite results to the ZFP64 knockdown group: in the ZFP64 overexpression group, the subcutaneous tumor growth rate was faster than that in the control group and the tumors were heavier (Figure 2F), whereas the ZFP64 knockdown group exhibited an obviously slower tumor growth rate and smaller tumors (Figure 2G). Therefore, ZFP64 could promote gallbladder cancer growth in vivo.

### 3.3. ZFP64 Activates Notch1 Signaling Pathways

**Figure 3:**
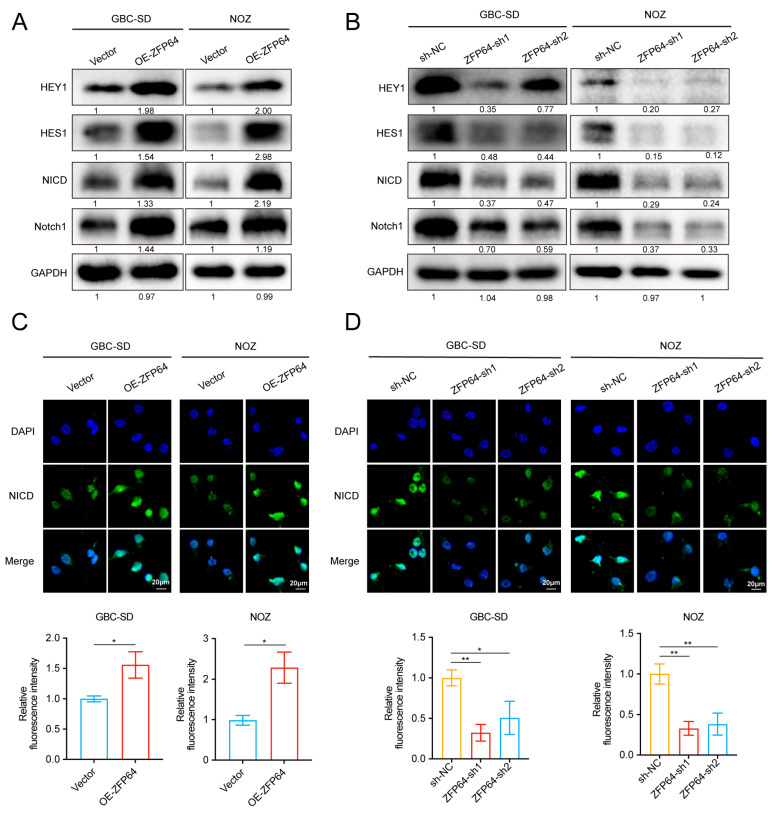
ZFP64 activates Notch1 signaling pathways. (**A**,**B**) Notch1 signaling pathway related protein levels in GBC cells overexpressing ZFP64 (**A**) or knocking down ZFP64 (**B**) were detected by Western blot assays. (**C**,**D**) Representative immunofluorescent staining images and statistical analysis of NICD in GBC-SD and NOZ cells overexpressing ZFP64 or knocking down ZFP64. NICD: green fluorescent; DAPI-stained nuclei: blue fluorescent. All data were assessed using unpaired Student’s *t* test or one-way ANOVA, as mean ± SD, * *p* < 0.05, ** *p* < 0.01, See Figures S4 and S5 for original western blots.

*HEY1* and *HES1* are endogenous Notch1 target genes and previous studies have shown that ZFP64 is a coactivator of NICD that can locate on the promoter regions of HEY1 and HES1 and then facilitate transcription [9]. In addition, in GBC patients, the Notch1 signaling pathway has been implicated in poor prognosis [21]. Therefore, ZFP64 may be involved in the regulation of GBC development through the Notch1 signaling pathway. Western blot assays showed that the increased expression of Notch1, NICD, *HES1*, and *HEY1* is due to the overexpression of ZFP64 (Figure 3A), whereas the expression of these essential molecules diminished when ZFP64 was knocked down (Figure 3B). NICD is a product of Notch hydrolysis by γ-secretase. The entry of NICD into the nucleus and the regulation of downstream gene transcription are two key events in the activation of Notch1 signaling pathways [22]. Therefore, we performed immunofluorescence to detect the protein level of NICD in the nucleus. The results showed that the level of NICD in the nucleus was significantly increased in the ZFP64 overexpression group (Figure 3C), while it decreased in the ZFP64 knockdown group (Figure 3D). Thus, ZFP64 is capable of activating the Notch1 signaling pathway.

### 3.4. ZFP64 Promotes Gallbladder Cancer Proliferation, Migration, and Invasion In Vitro via Activating Notch1 Signaling Pathway

**Figure 4:**
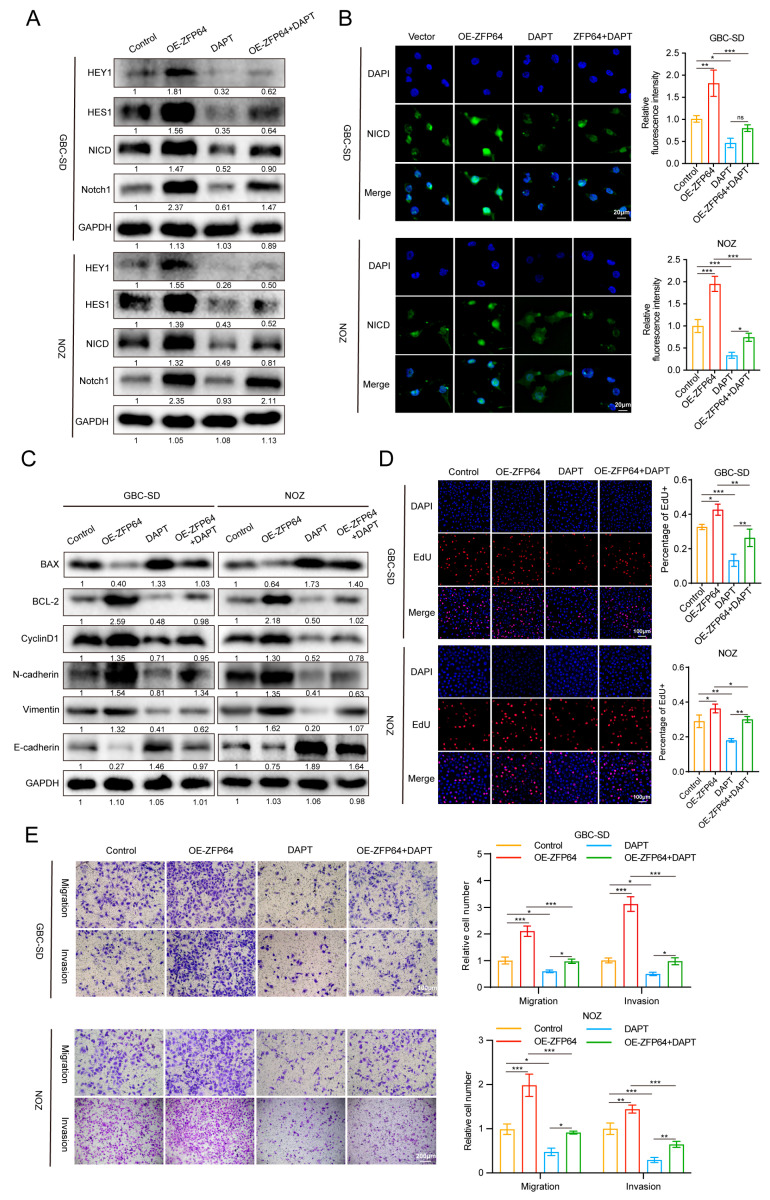
ZFP64 promotes gallbladder cancer proliferation, migration, and invasion in vitro via activating Notch1 signaling pathway. Control or ZFP64 overexpressing GBC-SD and NOZ cells were treated with or without DAPT at 100 μM for 24 h. (**A**) Western blot assays were used to analyze protein levels related to Notch1 signaling pathway. (**B**) Immunofluorescence assays were conducted to analyze the expression level of NICD. NICD: green fluorescent; DAPI-stained nuclei: blue fluorescent. (**C**) The ability of anti-apoptosis, proliferation, and EMT of GBC cells were detected using Western blot assays. (**D**) The proliferation capacity of GBC cells was determined using EdU assays. EdU positive cell: red fluorescent; DAPI-stained nuclei: blue fluorescent. (**E**) The migration and invasion were detected and counted using transwell assays. All data were assessed using one-way ANOVA, as mean ± SD, * *p* < 0.05, ** *p* < 0.01, *** *p* < 0.001. See Figures S6 and S7 for original western blots.

To further validate that ZFP64-promoted GBC procession through activating the Notch1 signaling pathway, we treated normal GBC cells and ZFP64-overexpressing GBC cells with or without the Notch1 signaling pathway inhibitor DAPT. We then used Western blot and immunofluorescence to detect the expression of key proteins in the Notch1 pathway. The results showed that, compared with the normal GBC cells, the protein levels of Notch1, NICD, HES1, and HEY1 in the ZFP64-overexpressing GBC cells were markedly increased, while the corresponding protein levels of the DAPT-treated GBC cells were substantially decreased. The protein levels of the combined treatment group of ZFP64 overexpression and DAPT were between those in the separate ZFP64 overexpression or DAPT groups (Figure 4A,B). Meanwhile, Western blot analysis also showed that with ZFP64 overexpressing, the expression levels of BCL-2, cyclinD1, N-cadherin, and vimentin were significantly elevated, while BAX and E-cadherin were downregulated. Within the treatment of DAPT, the expression levels of the above proteins were opposite to that of the ZFP64-overexpressing GBC cells. In addition, combined treatment with ZFP64 overexpression and DAPT led to the middle expression level of these critical proteins between the ZFP64-overexpressing GBC cells and the DAPT-treated GBC cells (Figure 4C). We also conducted EdU assays to examine the proportion of proliferating tumor cells in each group. The proportion of EdU-positive cells in the ZFP64-overexpressing GBC cells appeared to be higher than that in normal GBC cells, while the DAPT group had fewer proliferating cells. Consistent with the protein expression results, the EdU result of the DAPT and ZFP64 combined treatment group was between the two (Figure 4D). Furthermore, transwell assays demonstrated that the overexpression of ZFP64 significantly promoted the proliferation and invasion of GBC cells, while the use of DAPT obviously blocked GBC cell proliferation and invasion. The combined treatment of ZFP64 overexpression and DAPT demonstrated that DAPT obviously weakened the promotive effect of ZFP64 on GBC cells (Figure 4E). In summary, ZFP64 promotes the progression of gallbladder cancer through activating the Notch1 signaling pathway.

### 3.5. ZFP64 Activates the Notch1 Signaling Pathway by Recruiting HDAC1 to Inhibit NUMB Expression

**Figure 5:**
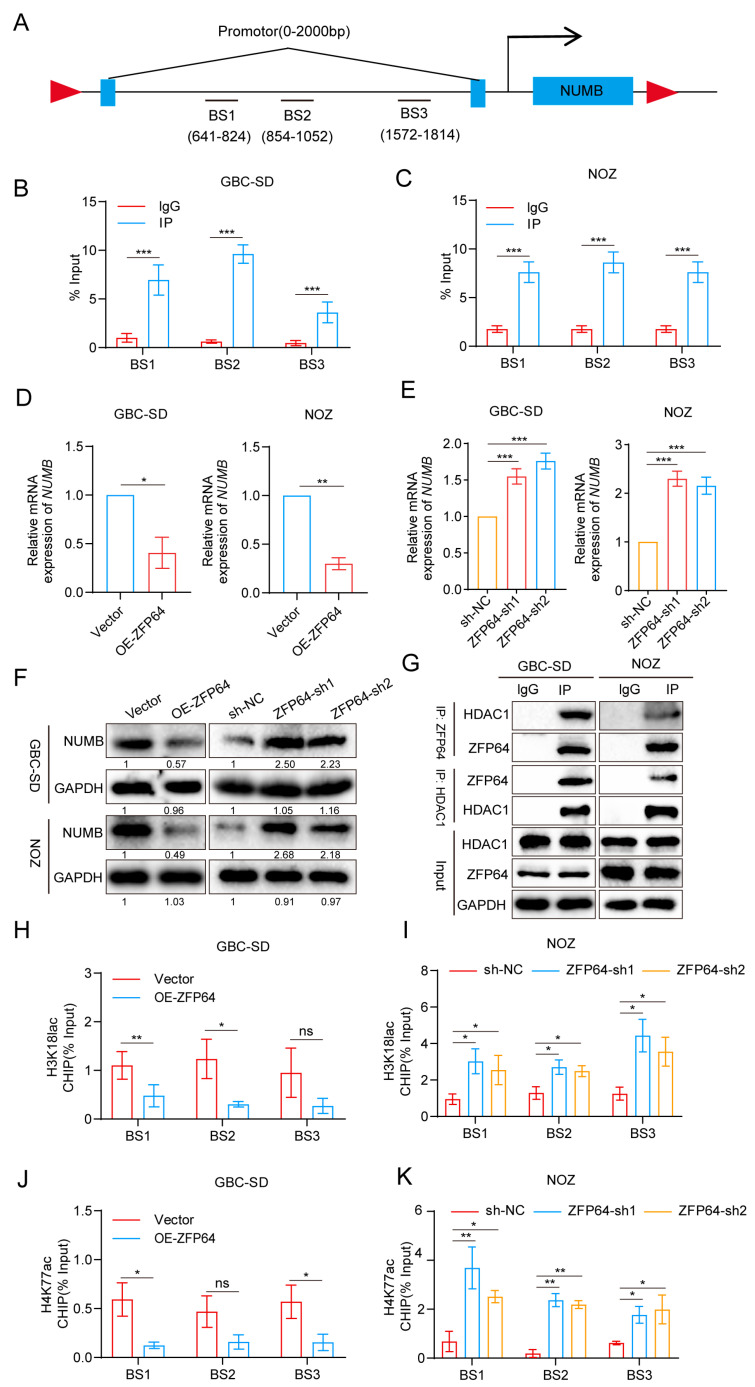
ZFP64 recruits HDAC1 to deacetylate and delactylate the promoter region of *NUMB* to suppress it. (**A**) Model diagram of the predicted ZFP64 binding *NUMB* promoter region. Arrow: the direction of transcription. (**B**,**C**) The direct interaction between ZFP64 and *NUMB* promoter region was detected using ChIP-qPCR in GBC-SD and NOZ cells. (**D**,**E**) The mRNA expression levels of *NUMB* in GBC cells overexpressing or knocking down ZFP64 was detected using RT-qPCR assays. (**F**) The protein expression levels of NUMB in GBC cells overexpressing or knocking down ZFP64 was detected using Western blot assays. (**G**) The direct interaction between ZFP64 and HDAC1 was confirmed using Co-IP assays and Western blot analysis. (**H**–**K**) The H3K18lac levels (**H**,**I**) and the H4K77ac level (**J**,**K**) in the *NUMB* promoter region of GBC-SD and NOZ cells with ZFP64 overexpression or knockdown were detected using ChIP-qPCR assays. Ns: no statistically significance. All data were assessed using unpaired Student’s *t* test or one-way ANOVA, as mean ± SD, * *p* < 0.05, ** *p* < 0.01, *** *p* < 0.001. See Figures S8 and S9 for original western blots.

To further investigate how ZFP64 activated the Notch1 signaling pathway, we speculated about the possible promoter region of the gene to which ZFP64 might bind and affect via JASPAR (http://jaspar.genereg.net/, accessed on 22 February 2023). As expected, we found that the promoter region of *NUMB*, a known inhibitor of the Notch1 signaling pathway, had the modification to which ZFP64 could bond (Figure 5A). Then, we designed three pairs of primers of the *NUMB* promoter region for further qPCR analysis according to the sites to which ZFP64 might bind. ChIP-qPCR assay further confirmed that ZFP64 directly interacted with the *NUMB* promoter region (Figure 5B,C). Thus, ZFP64 might influence the transcription of *NUMB* to subsequently affect the Notch1 signaling pathway. RT-qPCR and Western blot assays showed that the overexpression of ZFP64 down-regulated the mRNA and protein level of NUMB, whereas the knockdown of ZFP64 elevated NUMB expression (Figure 5D–F). ZFP64 acted as an inhibitory transcription factor to control NUMB expression.

Moreover, the mechanism of how ZFP64 inhibited NUMB expression was still unclear, so we combined co-immunoprecipitation (Co-IP) with mass spectrometry analysis to define the proteins that interacted with ZFP64 and might play the crucial role in the ZFP64 inhibition of NUMB expression. The 20 proteins with the highest abundance among the pull-down proteins were listed in Table S4. The results showed that, among these high abundance proteins, HDAC1 was identified as one of the potential ZFP64-interacting proteins because it has been widely recognized that HDAC1 is a key component of the histone deacetylase complex and is able to regulate gene expression using deacetylating histone [23] and delactylation [24]. The direct interaction of ZFP64 with HDAC1 probably meant that ZFP64 recruited HDAC1 to the promoter region of *NUMB*, where HDAC1 catalyzed deacetylation and delactylation to prevent *NUMB* from proper transcription and expression. Co-IP and Western blot assay further confirmed the interaction between ZFP64 and HDAC1(Figure 5G). Furthermore, we conducted ChIP assays and evaluated the influence of ZFP64 expression on the level of histone acetylation and the lactylation of the *NUMB* promoter region. Consistent with our speculation, the overexpression of ZFP64 apparently impaired the histone lactylation level at the H3K18 site and the histone acetylation level at the H4K77 site, while the results performed on ZFP64 knockdown GBC cells exhibited the reverse results (Figure 5H–K). These data indicated that the repressive effect of ZFP64 on NUMB was facilitated by the deacetylation and delactylation of the *NUMB* promoter region, which was due to the recruitment of ZFP64 to HDAC1.

**Figure 6:**
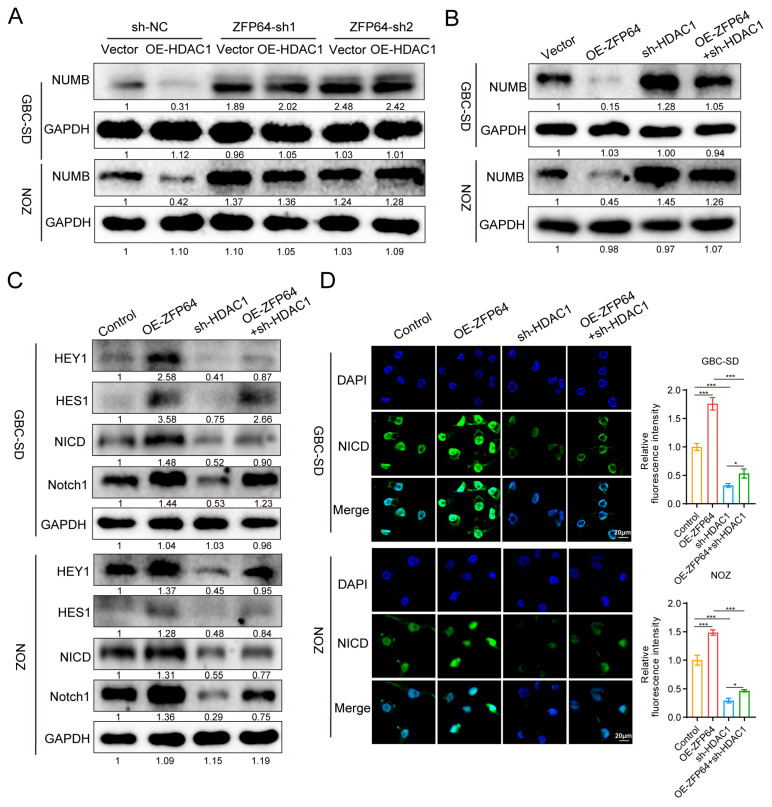
ZFP64 activates the Notch1 signaling pathway by recruiting HDAC1 to inhibit NUMB expression. (**A**) Control or ZFP64 knocking down GBC-SD and NOZ cells were transfected with vector or HDAC1 overexpressing plasmid for 24 h, and the NUMB protein expression levels were determined using Western blot assays. (**B**,**C**) Control or ZFP64 overexpressing GBC-SD and NOZ cells were transfected with shRNA plasmid of HDAC1 or corresponding empty plasmid. Western blot was used to detect the expression of NUMB (**B**) and the key molecules of Notch1 signaling pathway (**C**). (**D**) Immunofluorescence was performed to examine the expression level of NICD in nucleus. NICD: green fluorescent; DAPI-stained nuclei: blue fluorescent. All data were assessed using one-way ANOVA, as mean ± SD, * *p* < 0.05, *** *p* < 0.001. See Figures S10–S12 for original western blots.

In order to further elucidate the effect of the ZFP64–HDAC1 axis on the NUMB–Notch1 signaling pathway, HDAC1 was overexpressed in gallbladder cancer cell lines with ZFP64 knockdown. Western blot results showed that the overexpression of HDAC1 suppressed NUMB expression only in the control group. The inhibitory effect of HDAC1 on NUMB expression disappeared when ZFP64 was knocked down (Figure 6A), indicating that this inhibitory effect was dependent on the expression level of ZFP64. On the other hand, Western blot assays also demonstrated that, in GBC cells simultaneously overexpressing ZFP64 and knocking down HDAC1, the prohibitive effect of ZFP64 on NUMB and the promotive effect on the Notch1 signaling pathway were significantly reversed by HDAC1 knockdown (Figure 6B,C). The immunofluorescence of NICD further drew the same conclusion (Figure 6D).

## 4. Discussion

The lack of early markers and the poor treatment outcomes in the intermediate and advanced stage settings remain the most considerable obstacles to cure in GBC. Our study focused on the vital role of ZFP64 in GBC for the very first time. Through the RT-qPCR and the western blot assays, we demonstrated that ZFP64 expressed evidently higher in gallbladder carcinoma tissues from GBC patients than in normal tissues, and that GBC patients with high ZFP64 expression appeared to have an extremely worse prognosis. The expression level of ZFP64 was directly correlated with the prognosis of GBC patients and might positively serve as a reference indicator to aid in the prognosis of GBC and a biomarker for the early diagnosis of clinical GBC. In our exploration of more detailed mechanisms, we found that the overexpression of ZFP64 remarkably enhanced the proliferation, anti-apoptosis, invasion, migration, and EMT in both GBC-SD and NOZ cells in vitro and in vivo, which in turn promoted the metastasis and malignant development of GBC tumors. In addition, GBC can invade adjacent tissues through liver metastases, lymph node metastases, and vascular metastases, which has become another severe difficulty in the treatment of GBC [25,26]. Patients with high expression level of ZFP64 showed higher rates of lymph node metastasis, liver invasion, nerve invasion, and lower tumor differentiation, which might also be related to the facilitative effect of ZFP64 on the migration and invasion of GBC cells.

Histone acetylation, one of the most common histone epigenetic modifications, is in connection with multiple physiological and pathological diseases, in particular cancer. Two prime proteins engage in mediating this histone modification: histone acetyltransferases (HATs) add acetyl groups to histone lysine residues for acetylation modification, while histone deacetylases (HDACs) remove acetyl groups from histone lysines [27]. Belonging to the class I Rpd3-like family of HDACs, HDAC1 widely distributes in cells and is Zn^2+^-dependent for its catalytic function. HDAC1 is considered to be primary to mediate the regulation of epigenetic inheritance [28]. As the research progresses, HDAC1 has also been demonstrated to be apparently upregulated in a variety of cancers and the loss of HDAC1 interdicts tumor progression and induces apoptosis [29,30]. In addition, HDAC1 strengthens the repressive effect of some transcription factors on oncoprotein promoters by deacetylating these transcription factors. For example, in breast cancer, HDAC1 deacetylated *SREBP1* and inhibited *E-calmodulin* transcription, thereby suppressing the EMT [31]. In hepatoblastoma (HBL), HDAC1 delivered to the promoter of the *p21* gene by another transcription factor, sp5, and activated p21 expression, thereby promoting the proliferation and metastasis of HBL cells [32]. In addition, inhibitors of HDAC1 have been developed and placed in more well-established pre-clinical and clinical systems to treat cancers, including colorectal cancer, hepatocellular carcinoma, and neuroblastoma [33,34]. In GBC, HDAC1 was first shown to interact with the transcription factor TCF-12, driving GBC tumor invasion and leading to poor prognosis [35]. Combined treatment with the HDAC inhibitor SAHA and the BRD4 inhibitor JQ1 could resist GBC cell proliferation and viability and induced apoptosis, thereby reducing the tumorigenic capacity of GBC cells in vivo [36].

Histone lactylation is another epigenetic modification present in histones, and like histone acetylation modifications, it imposes gene transcription with analogous molecular mechanisms [37]. As the Warburg effect describes, tumor cells produce large amounts of lactic acid through aerobic glycolysis [38]. The degree of lactylation is dependent on the production of endogenous lactic acid, and the accumulation of endogenous lactic acid provides the indispensable substrate for lactylation [37,39]. Recent studies have expounded the role of histone lactylation in some cancers. In ocular melanoma, a lactylation increase in H3K18 sites at the *YTHDF2* promoter region improved *YTHDF2* expression and sustained ocular melanoma malignization [40]. In addition, two lactylation sites, H3K9 lac and H3K56 lac, were also known to contribute to HCC development. Meanwhile, the demethylzeylasteral (DML) has been shown to be equipped to control glycolysis and gluconeogenesireduce, therefore reducing lactate levels in hepatocellular carcinoma stem cells (LCSC) and preventing LCSC tumorigenesis [41]. In addition to deacetylating histones, HDAC1 also acts as a lysine delactylase to delactylate histone lysines [24]. However, the function of HDAC1 as a delactylase in tumors still remains unknown. Here, we proved that HDAC1 interacted with ZFP64 directly. In GBC cell lines overexpressing ZFP64, mass spectrometry identification confirmed lessened levels of H3K18 lactylation and H4K77 acetylation in the *NUMB* promoter region. Further Co-IP confirmed that HDAC1 principally mediated this process: ZFP64 recruited HDAC1 to locate on the promoter region of *NUMB* and modified it through deacetylation and delactylation. Subsequently, this modification suppressed NUMB expression, thereby activating the Notch1 pathway and upregulating the expressions of Notch1 pathway-related proteins HEY1, HES1, and NICD. The Notch1 signaling pathway inhibitor DAPT abrogated the promotive effects of ZFP64 on the proliferation and metastasis of GBC cells, which further demonstrated that ZFP64 regulated GBC development through the Notch1 signaling pathway.

## 5. Conclusions

Motivated by the current poor therapeutic efficacy of GBC, our study aims at developing new methods for GBC treatment. In conclusion, we provide evidence to support that ZFP64 is a provital regulatory protein in GBC progression. Furthermore, we also demonstrated that ZFP64-HDAC1-NUMB-Notch1 is an essential oncogenic axis in GBC that promotes multiple malignant biological behaviors of GBC cells. We highlight that targeting ZFP64 may be an effective avenue for future GBC therapy.

## Figures and Tables

**Table 1:** The primers of *NUMB* promoter for ChIP-qPCR assay.

Primer Names	Sequences
NUMB BS1 forward	TGGCGTATTGAGAGTTCTCC
NUMB BS1 reverse	AACCTGGGAGGCGTAGGTTGC
NUMB BS2 forward	TAGCTGGGATTATAGGCATGA
NUMB BS2 reverse	GCAGAATTCTCATTTCCAG
NUMB BS3 forward	CATGCCTGTTATCCCAGCACT
NUMB BS3 reverse	CTTTGTCTCTCTTTCTTCTTTCT

**Table 2:** Correlation between the expression of ZFP64 and the clinicopathological features in 50 cases of gallbladder carcinoma.

Clinicopathological Features		ZFP64 Expression			
		Cases	Low	High	*p* Value
Age	<60	22	12	10	0.569
	≥60	28	13	15	
Gender	Male	20	11	9	0.564
	Female	30	14	15	
CA19-9 level	≤37 U/mL	31	21	10	0.001
	>37 U/mL	19	4	15	
Tumor size	≤3 cm	30	18	12	0.083
	>3 cm	20	7	13	
Hepatic invasion	No	26	19	7	0.002
	Yes	24	6	18	
Lymph node metastasis	No	26	19	7	0.002
	Yes	24	6	18	
Neuro invasion	No	38	24	14	0.001
	Yes	12	1	11	
Vascular invasion	No	45	25	0	0.059
	Yes	5	0	5	
Tumor differentiation	High moderate	29	18	11	0.045
	Low moderate	21	7	14	

## Data Availability

All data are contained within the article or Supplementary Materials.

## Supplementary Materials

The following supporting information can be downloaded at: https://www.mdpi.com/article/10.3390/cancers15184508/s1, Figure S1. GBC-SD and NOZ cells were generated to stably overexpress or knockdown ZFP64 via lentivirus transfection for 24 h, and then 10 μg/mL of puromycin was added to screen successfully infected cells; Figure S2. Uncropped Western Blot images for Figure 1D; Figure S3. Uncropped Western Blot images for Figure 2E; Figure S4. Uncropped Western Blot images for Figure 3A; Figure S5. Uncropped Western Blot images for Figure 3B; Figure S6. Uncropped Western Blot images for Figure 4A; Figure S7. Uncropped Western Blot images for Figure 4C; Figure S8. Uncropped Western Blot images for Figure 5F; Figure S9. Uncropped Western Blot images for Figure 5G; Figure S10. Uncropped Western Blot images for Figure 6A; Figure S11. Uncropped Western Blot images for Figure 6B; Figure S12. Uncropped Western Blot images for Figure 6C; Figure S13. Uncropped Western Blot images for Figure S1C; Figure S14. Uncropped Western Blot images for Figure 5E; Table S1. Prognostic factors for overall survival by the univariate and multivariate cox proportional hazards regression model; Table S2. Prognostic factors for disease-free survival by the univariate and multivariate cox proportional hazards regression model; Table S3. Summary of primary antibody information used in this article; Table S4. Top twenty ZFP64 pull-down proteins analyzed by mass spectrum.
