# Supplementary material for: ZFP64 Promotes Gallbladder Cancer Progression through Recruiting HDAC1 to Activate NOTCH1 Signaling Pathway
Source: Cancers (Basel). 2023 Sep 11;15(18):4508. doi: 10.3390/cancers15184508 (PMC10527061; doi:10.3390/cancers15184508)
Supplement: Supplementary file 1 [file cancers-15-04508-s001.zip › cancers-2573702-supplementary/Table S1.pdf]

Clinicopathological features		Univariate Cox			Multivariate Cox	
		Cases	Survival (Month)	*P* value	HR (95% CI)	*P* value
Age	< 60	22	33	0.559		
	≥ 60	28	25.5			
Gender	Male	20	24	0.132		
	Female	30	33			
CA19-9 level	≤ 37 U/ml	31	42	<0.001	3.741 (1.661-8.425)	0.001
	> 37 U/ml	19	19			
Tumor size	≤ 3 cm	30	37	0.028	1.055 (0.436-2.549)	0.906
	> 3 cm	20	20			
Hepatic invasion	No	26	54	<0.001	2.587 (0.730-9.173)	0.141
	Yes	24	20			
Lymph node metastasis	No	26	54	<0.001	1.602 (0.442-5.813)	0.473
	Yes	24	20			
Neuro invasion	No	38	33	0.004	3.142 (0.997-9.906)	0.051
	Yes	12	20			
Vascular invasion	No	45	33	<0.001	2.715 (0.619-11.919)	0.186
	Yes	5	13			
Tumor differentiation	No	29	36	0.051	1.748 (0.734-4.163)	0.207
	Yes	21	22			
	Low	25	54	<0.001	2.980	0.031

ZFP64 expression level	High	25	20		(1.105-8.039)	

Table S1. Prognostic factors for overall survival by the univariate and multivariate cox proportional hazards regression model.
