# Supplementary material for: ZFP64 Promotes Gallbladder Cancer Progression through Recruiting HDAC1 to Activate NOTCH1 Signaling Pathway
Source: Cancers (Basel). 2023 Sep 11;15(18):4508. doi: 10.3390/cancers15184508 (PMC10527061; doi:10.3390/cancers15184508)
Supplement: Supplementary file 1 [file cancers-15-04508-s001.zip › cancers-2573702-supplementary/Table S2.pdf]

Clinicopathological features		Univariate Cox			Multivariate Cox	
		Cases	Survival (Month)	*P* value	HR (95% CI)	*P* value
Age	< 60	22	22	0.661		
	≥ 60	28	21			
Gender	Male	20	20	0.205		
	Female	30	22			
CA19-9 level	≤ 37 U/ml	31	29	0.046	1.357	0.455
	> 37 U/ml	19	19		(0.610-3.019)	
Tumor size	≤ 3 cm	30	30	0.030	1.131	0.781
	> 3 cm	20	17		(0.473-2.704)	
Hepatic invasion	No	26	40	<0.001	2.650	0.106
	Yes	24	17		(0.813-8.636)	
Lymph node metastasis	No	26	40	<0.001	1.497	0.507
	Yes	24	17		(0.454-4.940)	
Neuro invasion	No	38	29	<0.001	1.546	0.490
	Yes	12	13		(0.449-5.326)	
Vascular invasion	No	45	25	<0.001	2.469	0.237
	Yes	5	10		(0.552-11.047)	
Tumor differentiation	No	29	29	0.041	1.532	0.328
	Yes	21	19		(0.652-3.596)	
	Low	25	45	<0.001	3.191	0.014

ZFP64 expression level	High	25	15	(1.270-8.019)

Table S2. Prognostic factors for disease-free survival by the univariate and multivariate cox proportional hazards regression model.
