# Supplementary material for: ZFP64 Promotes Gallbladder Cancer Progression through Recruiting HDAC1 to Activate NOTCH1 Signaling Pathway
Source: Cancers (Basel). 2023 Sep 11;15(18):4508. doi: 10.3390/cancers15184508 (PMC10527061; doi:10.3390/cancers15184508)
Supplement: Supplementary file 1 [file cancers-15-04508-s001.zip › cancers-2573702-supplementary/Table S3.pdf]

Antibody	Company	Cat. No.
Notch1	Cell Signaling	3608
NICD	Cell Signaling	4147
HES1	HUABIO	ET1610-97
HEY1	Proteintech	19929-1-AP
NUMB	HUABIO	ET1703-02
BAX	HUABIO	ET1603-34
BCL-2	HUABIO	ER0602
CyclinD1	Proteintech	60186-1-Ig
N-cadherin	Proteintech	66219-1-Ig
E-cadherin	Proteintech	60335-1-Ig
Vimentin	Proteintech	60330-1-Ig
ZFP64	Proteintech	17187-1-AP
H3K18lac	PTMBIO	PTM-1427RM
H4K77ac	PTMBIO	PTM-127RM
HDAC1	Proteintech	66085-1-Ig

Table S3. Summary of primary antibody information used in this article.
