# Supplementary material for: ZFP64 Promotes Gallbladder Cancer Progression through Recruiting HDAC1 to Activate NOTCH1 Signaling Pathway
Source: Cancers (Basel). 2023 Sep 11;15(18):4508. doi: 10.3390/cancers15184508 (PMC10527061; doi:10.3390/cancers15184508)
Supplement: Supplementary file 1 [file cancers-15-04508-s001.zip › cancers-2573702-supplementary/Table S4.pdf]

Accession	Gene Symbol	Description	Score Sequest HT: Sequest HT
Q15149	PLEC	Plectin OS=Homo sapiens OX=9606 GN=PLEC PE=1 SV=3	409.03
P35579	MYH9	Myosin-9 OS=Homo sapiens OX=9606 GN=MYH9 PE=1 SV=4	111.43
O00159	MYO1C	Unconventional myosin-Ic OS=Homo sapiens OX=9606 GN=MYO1C PE=1 SV=4	39.77
Q09666	AHNAK	Neuroblast differentiation-associated protein AHNAK OS=Homo sapiens OX=9606 GN=AHNAK PE=1 SV=2	27.29
P11142	HSPA8	Heat shock cognate 71 kDa protein OS=Homo sapiens OX=9606 GN=HSPA8 PE=1 SV=1	32.67
Q13547	HDAC1	Histone deacetylase 1 OS=Homo sapiens OX=9606 GN=HDAC1 PE=1 SV=1	32.47
P08729	KRT7	Keratin, type II cytoskeletal 7 OS=Homo sapiens OX=9606 GN=KRT7 PE=1 SV=5	29.08
O75369	FLNB	Filamin-B OS=Homo sapiens OX=9606 GN=FLNB PE=1 SV=2	24.37
Q9P0K7	RAI14	Ankycorbin OS=Homo sapiens OX=9606 GN=RAI14 PE=1 SV=2	28.11
P21333	FLNA	Filamin-A OS=Homo sapiens OX=9606 GN=FLNA PE=1 SV=4	25.9
Q6WCQ1	MPRIP	Myosin phosphatase Rho-interacting protein OS=Homo sapiens OX=9606 GN=MPRIP PE=1 SV=3	26.87
P02545	LMNA	Prelamin-A/C OS=Homo sapiens OX=9606 GN=LMNA PE=1 SV=1	25.53
P07355	ANXA2	Annexin A2 OS=Homo sapiens OX=9606 GN=ANXA2 PE=1 SV=2	22.83
Q9BZF9	UACA	Uveal autoantigen with coiled-coil domains and ankyrin repeats OS=Homo sapiens OX=9606 GN=UACA PE=1 SV=2	29.54
P08727	KRT19	Keratin, type I cytoskeletal 19 OS=Homo sapiens OX=9606 GN=KRT19 PE=1 SV=4	21.18
P13645	KRT10	Keratin, type I cytoskeletal 10 OS=Homo sapiens OX=9606 GN=KRT10 PE=1 SV=6	19.15

Q8WWI1	LMO7	LIM domain only protein 7 OS=Homo sapiens OX=9606 GN=LMO7 PE=1 SV=3	18.3
P14618	PKM	Pyruvate kinase PKM OS=Homo sapiens OX=9606 GN=PKM PE=1 SV=4	19.09
Q9UHB6	LIMA1	LIM domain and actin-binding protein 1 OS=Homo sapiens OX=9606 GN=LIMA1 PE=1 SV=1	17.02
P11940	PABPC1	Polyadenylate-binding protein 1 OS=Homo sapiens OX=9606 GN=PABPC1 PE=1 SV=2	6.94

Table S4. Top twenty ZFP64 pull-down proteins analyzed by mass spectrum.
