# Supplementary figures and images for: ZFP64 Promotes Gallbladder Cancer Progression through Recruiting HDAC1 to Activate NOTCH1 Signaling Pathway (part 2 of 2)
Source: Cancers (Basel). 2023 Sep 11;15(18):4508. doi: 10.3390/cancers15184508 (PMC10527061; doi:10.3390/cancers15184508)

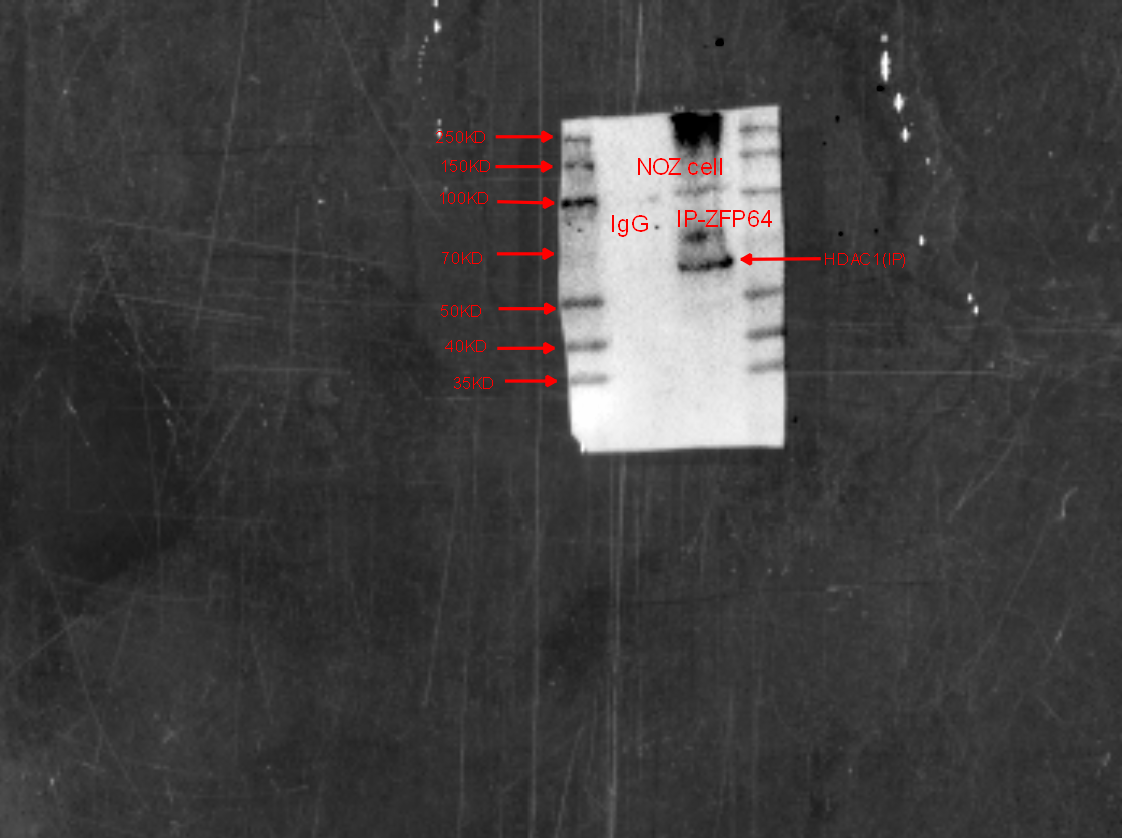

Supplement: Supplementary file 1 [file cancers-15-04508-s001.zip › cancers-2573702-supplementary/Figure S8-Figure 5F/HDAC1 (NOZ).tif]

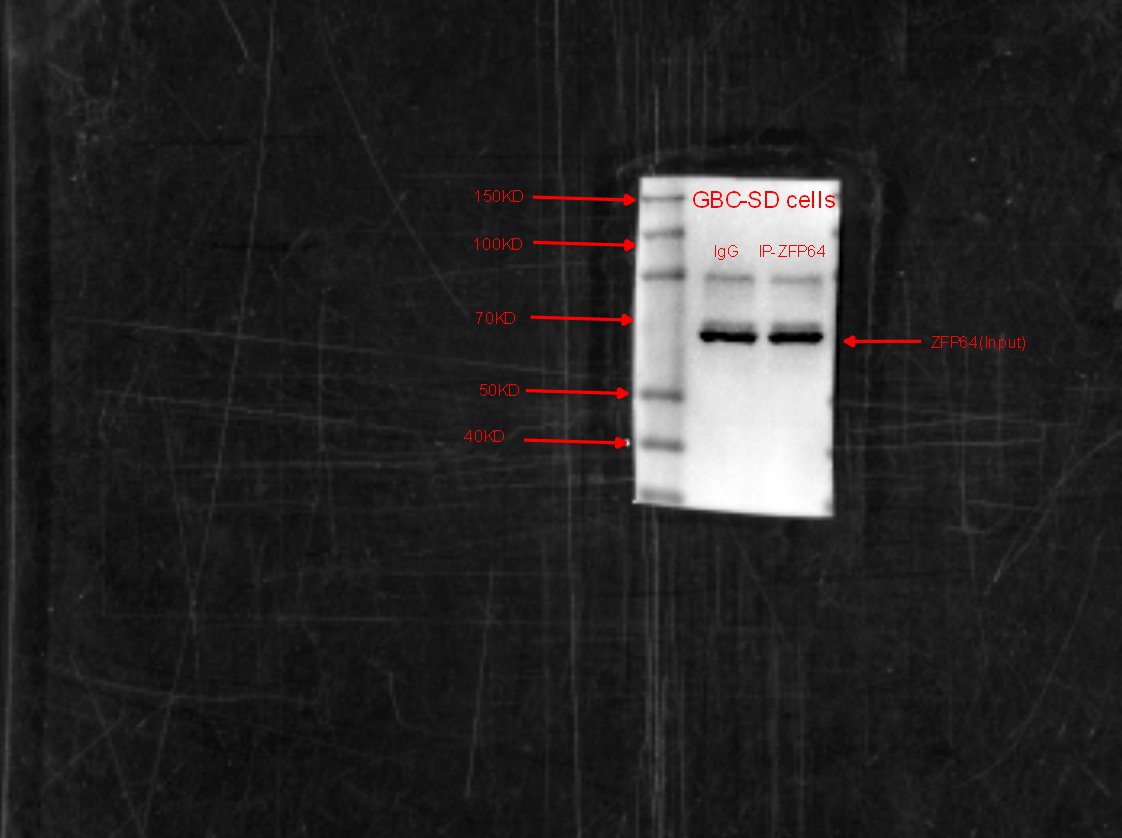

Supplement: Supplementary file 1 [file cancers-15-04508-s001.zip › cancers-2573702-supplementary/Figure S8-Figure 5F/ZFP64 (GBC-SD).tif]

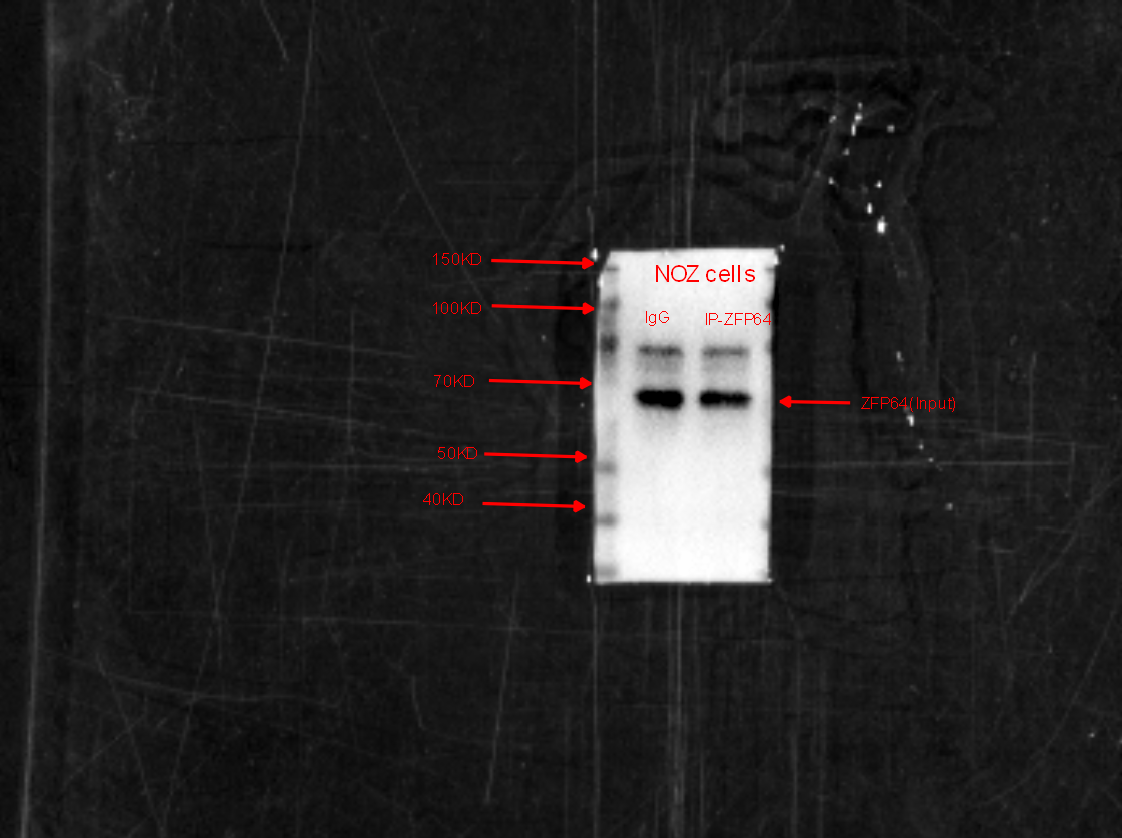

Supplement: Supplementary file 1 [file cancers-15-04508-s001.zip › cancers-2573702-supplementary/Figure S8-Figure 5F/ZFP64 (NOZ) (2).tif]

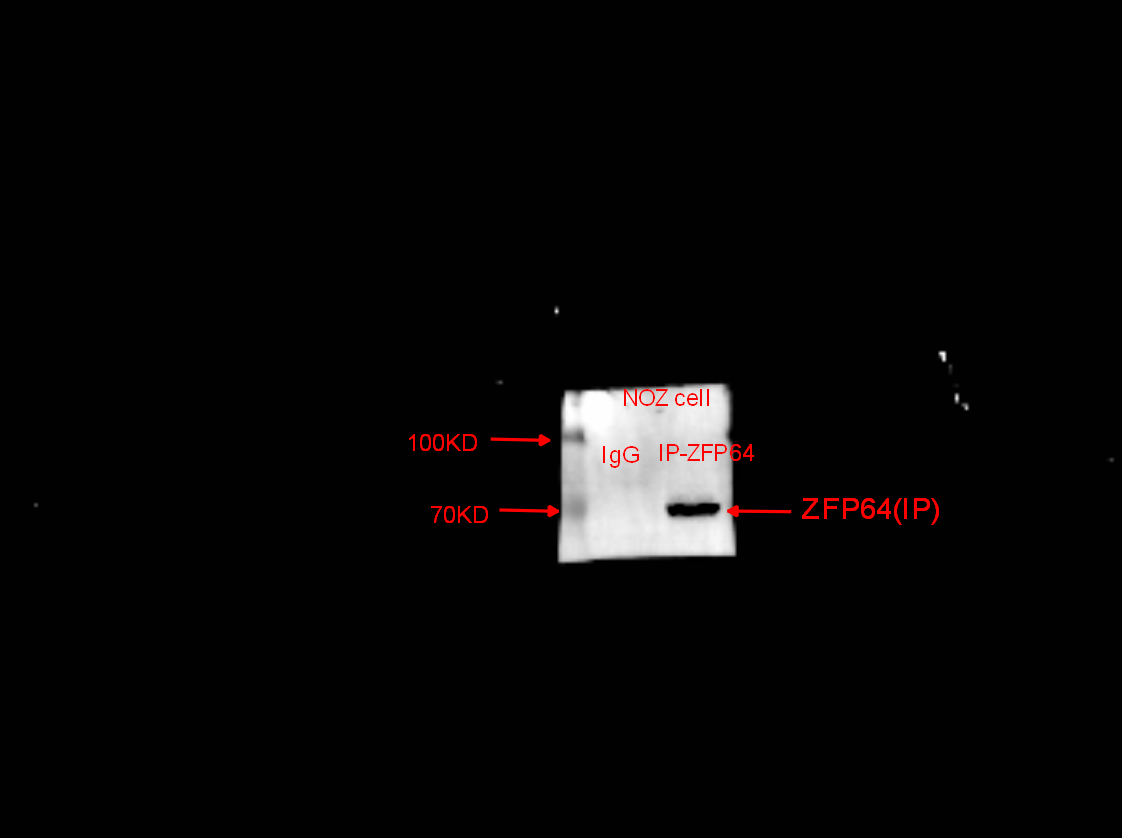

Supplement: Supplementary file 1 [file cancers-15-04508-s001.zip › cancers-2573702-supplementary/Figure S8-Figure 5F/ZFP64 (NOZ).tif]

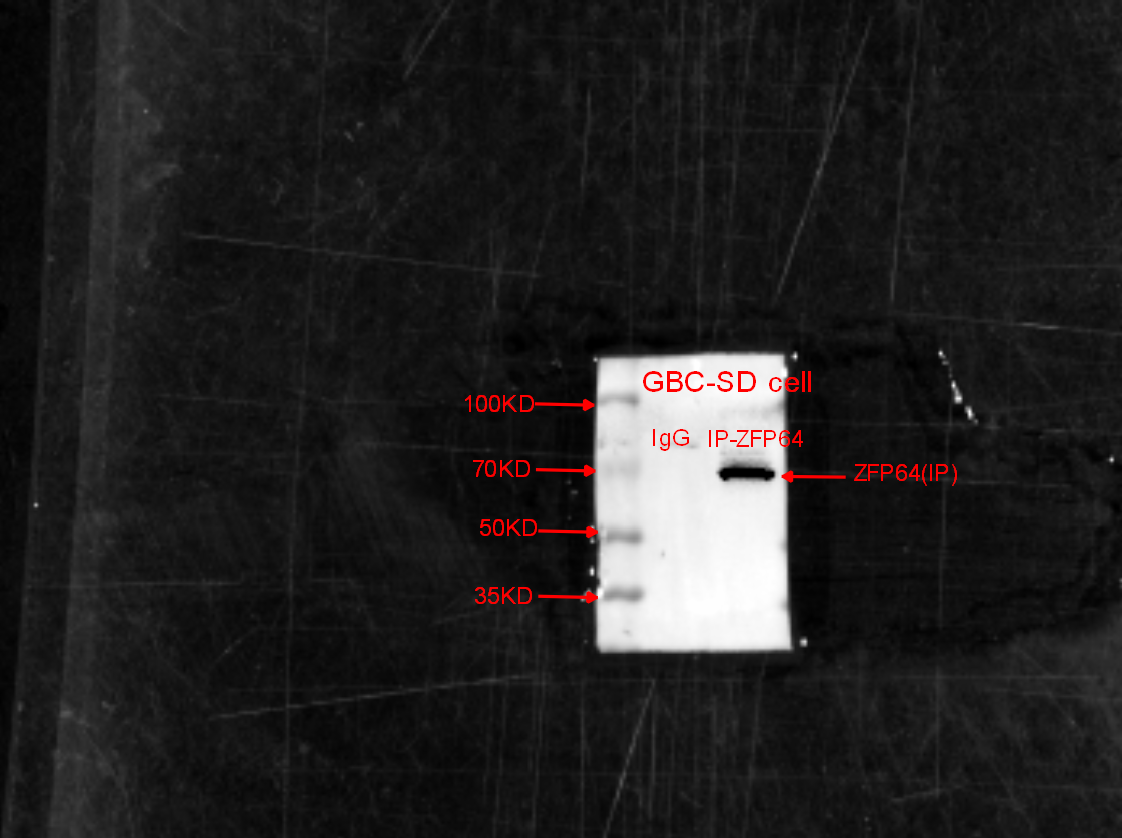

Supplement: Supplementary file 1 [file cancers-15-04508-s001.zip › cancers-2573702-supplementary/Figure S8-Figure 5F/ZFP64 (SD).tif]

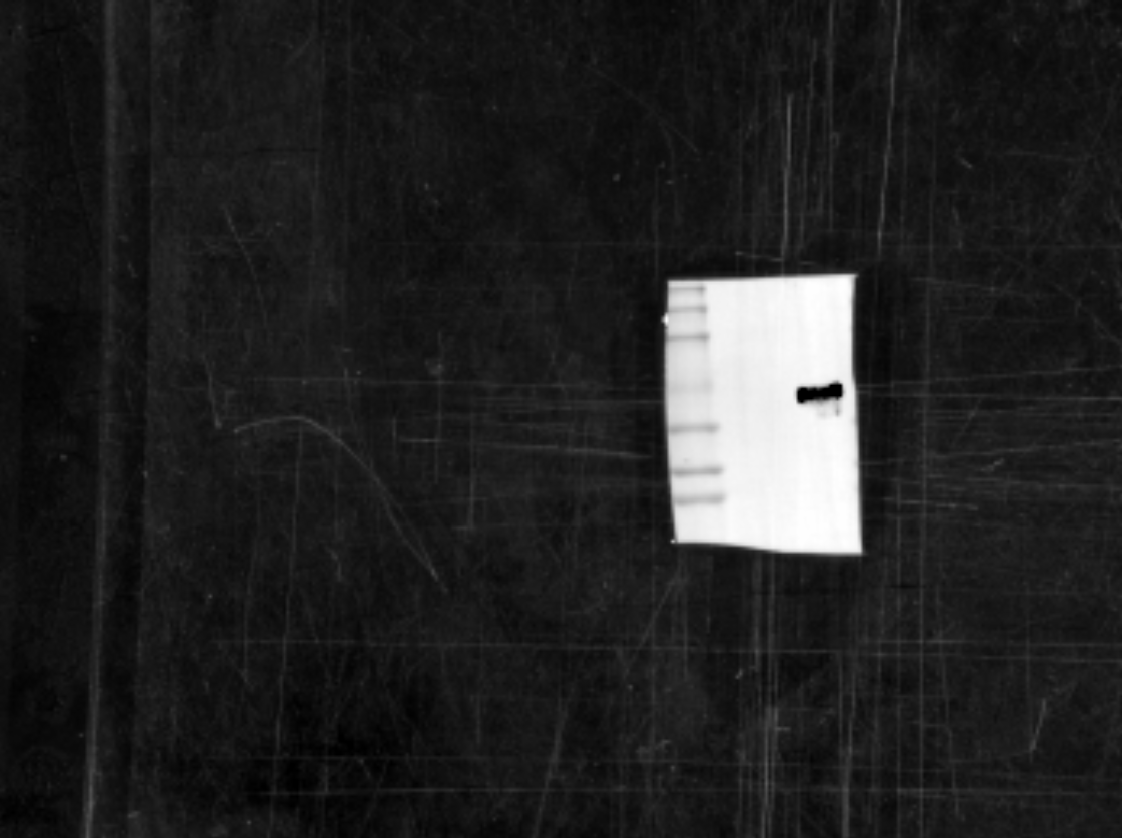

Supplement: Supplementary file 1 [file cancers-15-04508-s001.zip › cancers-2573702-supplementary/Figure S9-Figure 5G/HDAC1(GBC-SD).tif]

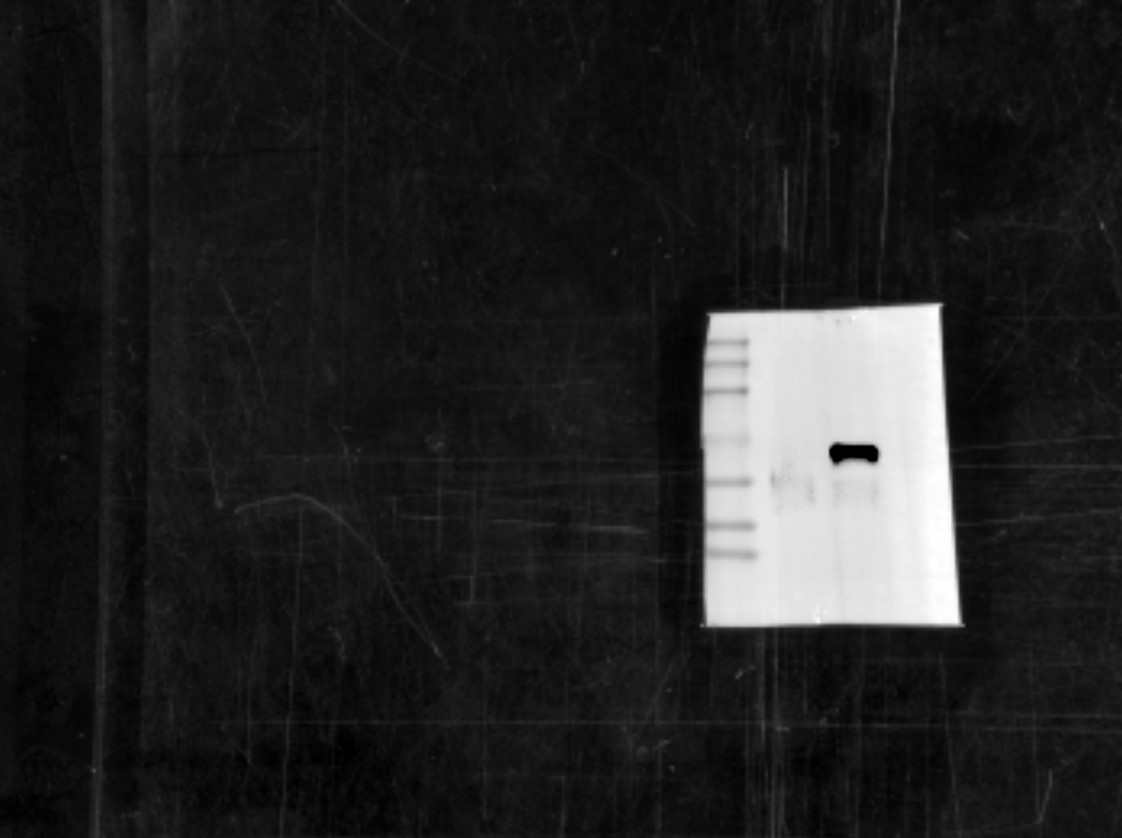

Supplement: Supplementary file 1 [file cancers-15-04508-s001.zip › cancers-2573702-supplementary/Figure S9-Figure 5G/HDAC1(NOZ).tif]

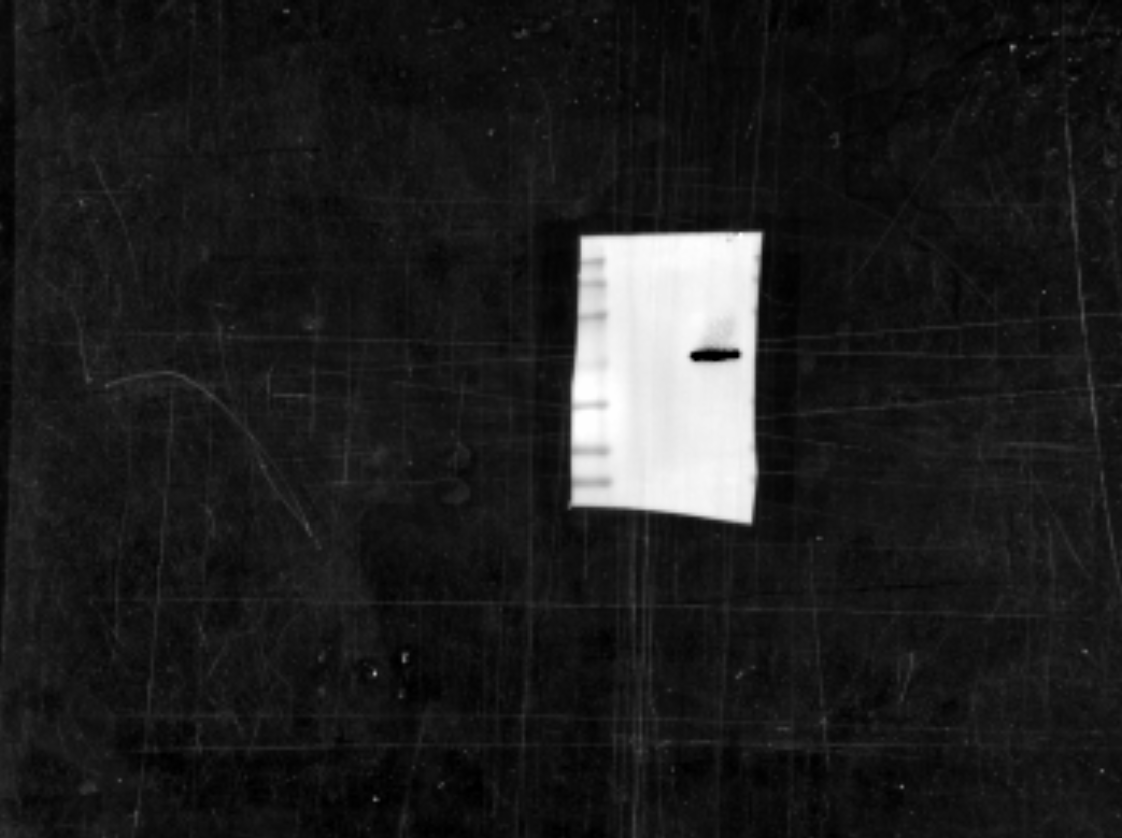

Supplement: Supplementary file 1 [file cancers-15-04508-s001.zip › cancers-2573702-supplementary/Figure S9-Figure 5G/ZFP64(GBC-SD).tif]

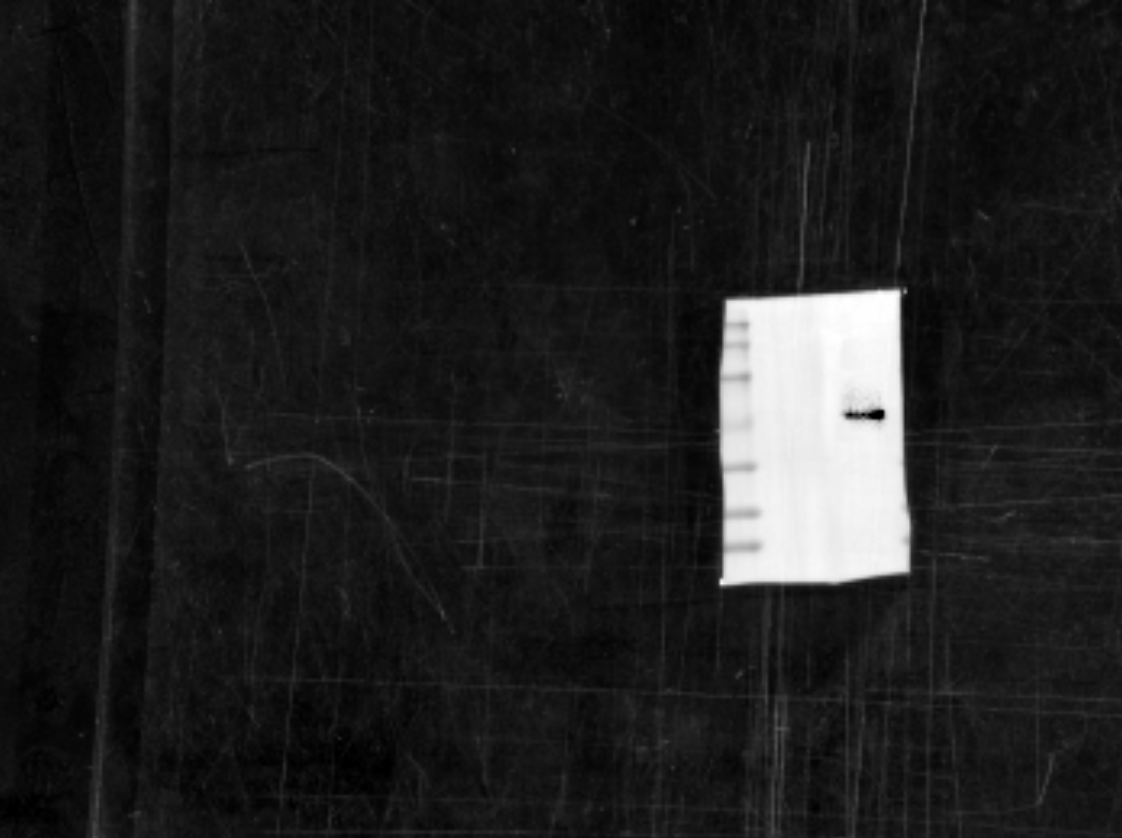

Supplement: Supplementary file 1 [file cancers-15-04508-s001.zip › cancers-2573702-supplementary/Figure S9-Figure 5G/ZFP64(NOZ).tif]
