# Supplementary figures and images for: ZFP64 Promotes Gallbladder Cancer Progression through Recruiting HDAC1 to Activate NOTCH1 Signaling Pathway (part 1 of 2)
Source: Cancers (Basel). 2023 Sep 11;15(18):4508. doi: 10.3390/cancers15184508 (PMC10527061; doi:10.3390/cancers15184508)

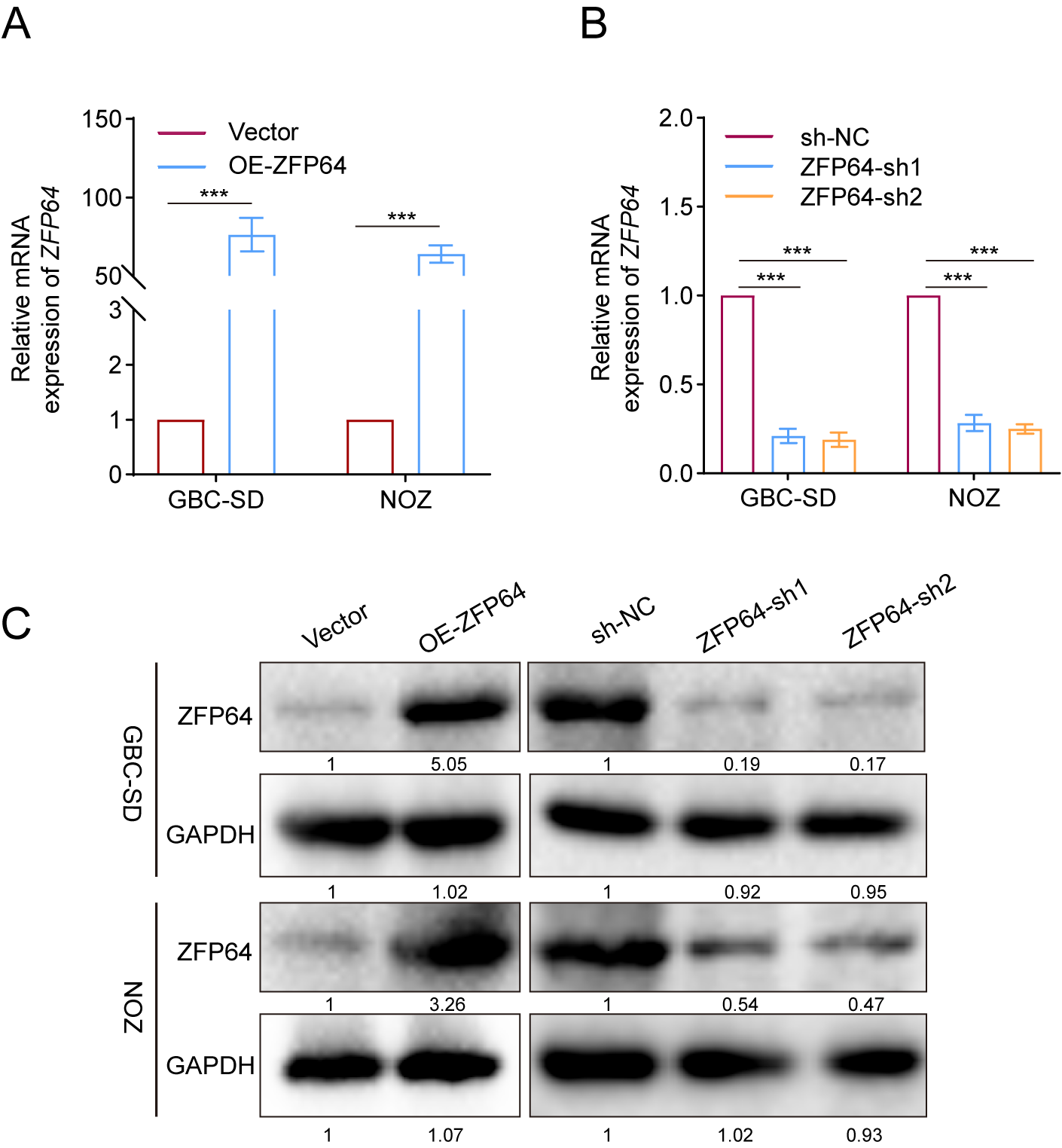

Supplement: Supplementary file 1 [file cancers-15-04508-s001.zip › cancers-2573702-supplementary/Figure S1.tif]

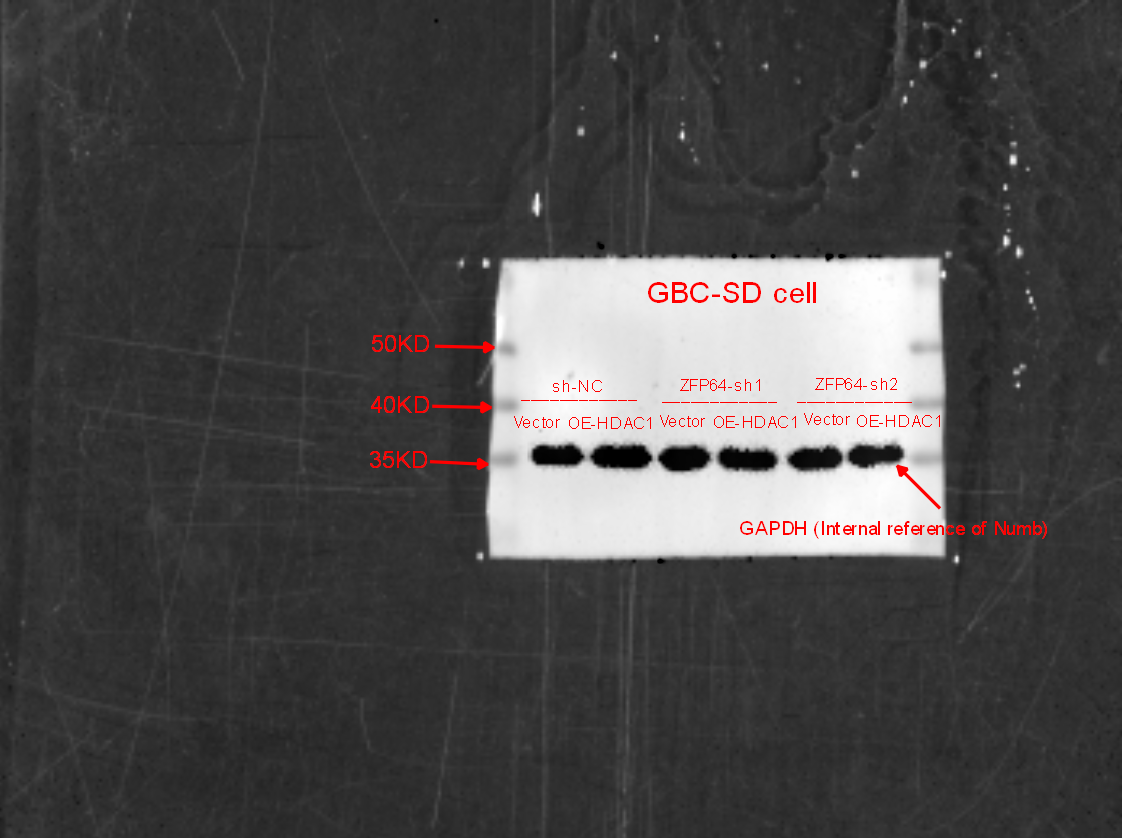

Supplement: Supplementary file 1 [file cancers-15-04508-s001.zip › cancers-2573702-supplementary/Figure S10-Figure 6A/GAPDH (GBC-SD).tif]

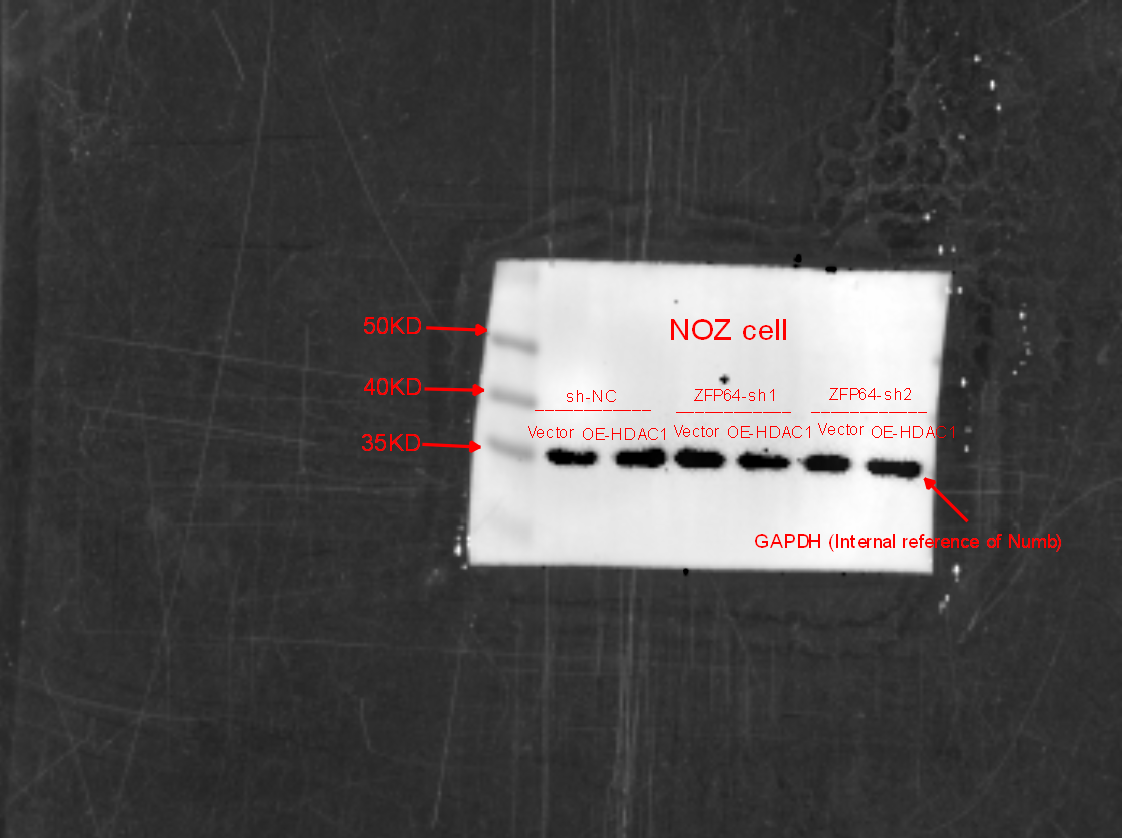

Supplement: Supplementary file 1 [file cancers-15-04508-s001.zip › cancers-2573702-supplementary/Figure S10-Figure 6A/GAPDH (NOZ).tif]

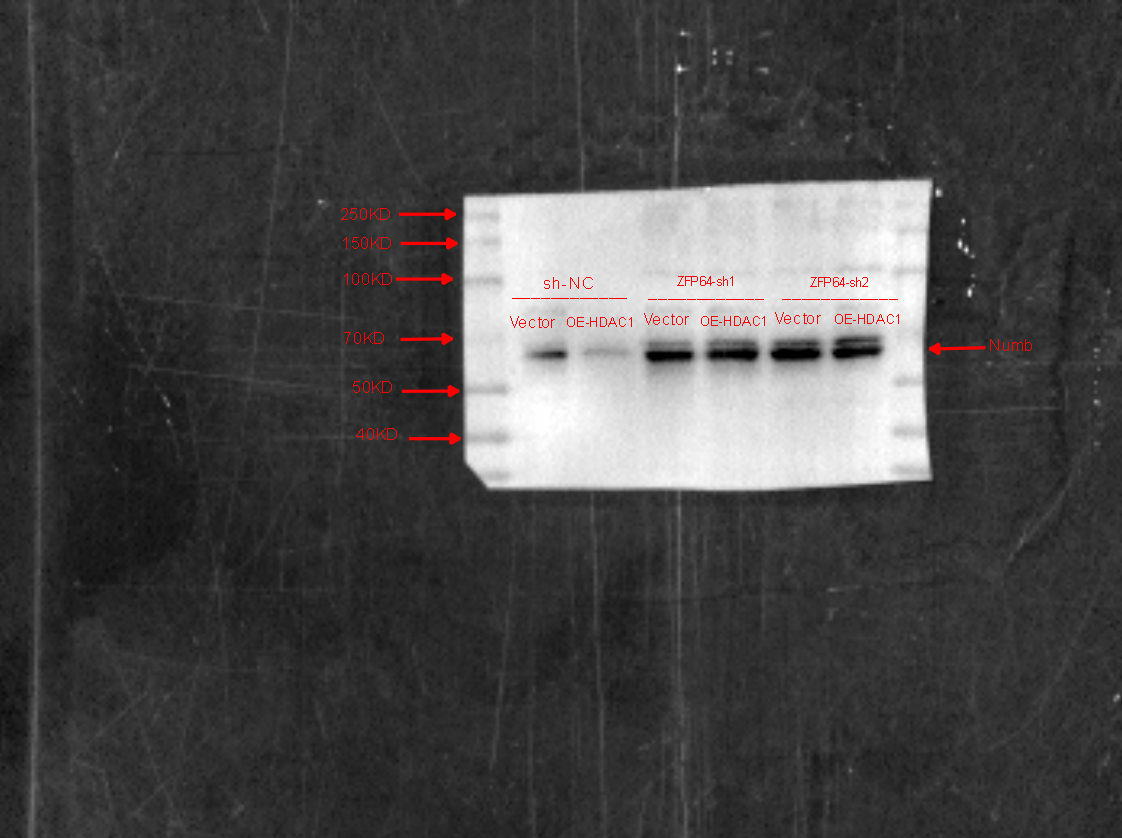

Supplement: Supplementary file 1 [file cancers-15-04508-s001.zip › cancers-2573702-supplementary/Figure S10-Figure 6A/NUMB (GBC-SD).tif]

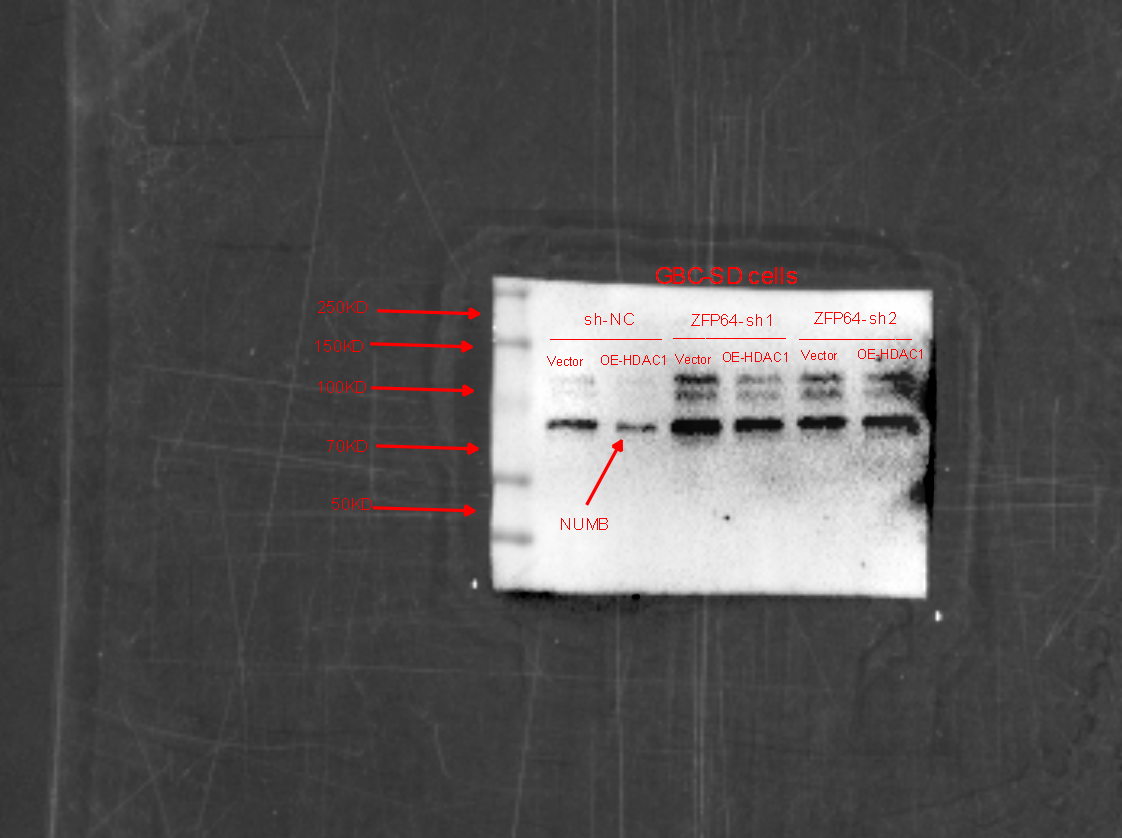

Supplement: Supplementary file 1 [file cancers-15-04508-s001.zip › cancers-2573702-supplementary/Figure S10-Figure 6A/NUMB (NOZ).tif]

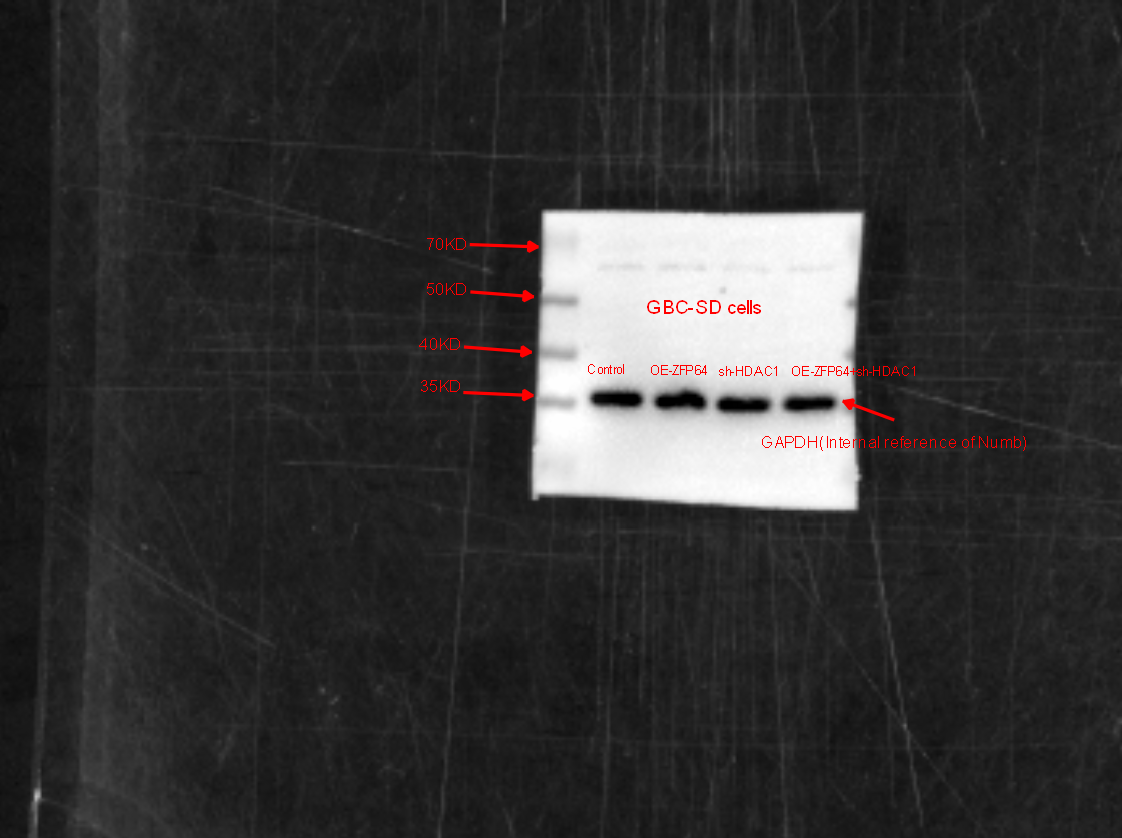

Supplement: Supplementary file 1 [file cancers-15-04508-s001.zip › cancers-2573702-supplementary/Figure S11-Figure 6B/GAPDH (GBC-SD).tif]

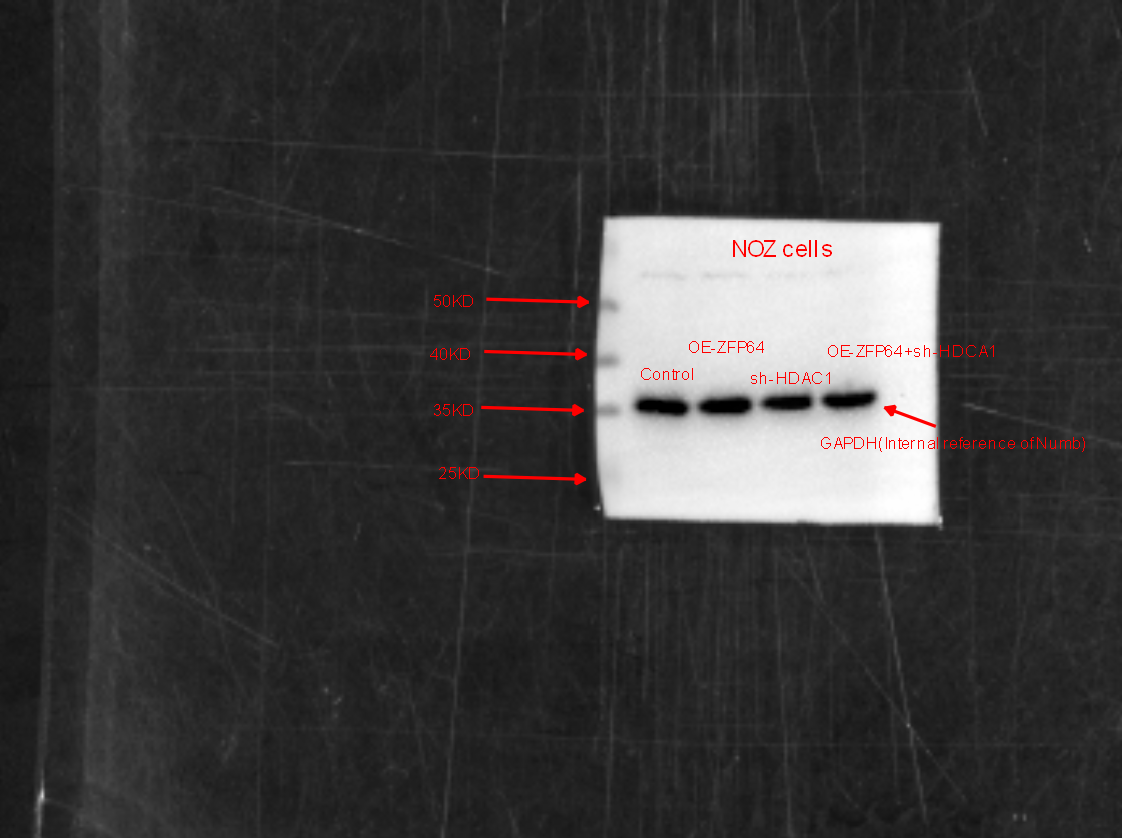

Supplement: Supplementary file 1 [file cancers-15-04508-s001.zip › cancers-2573702-supplementary/Figure S11-Figure 6B/GAPDH (NOZ).tif]

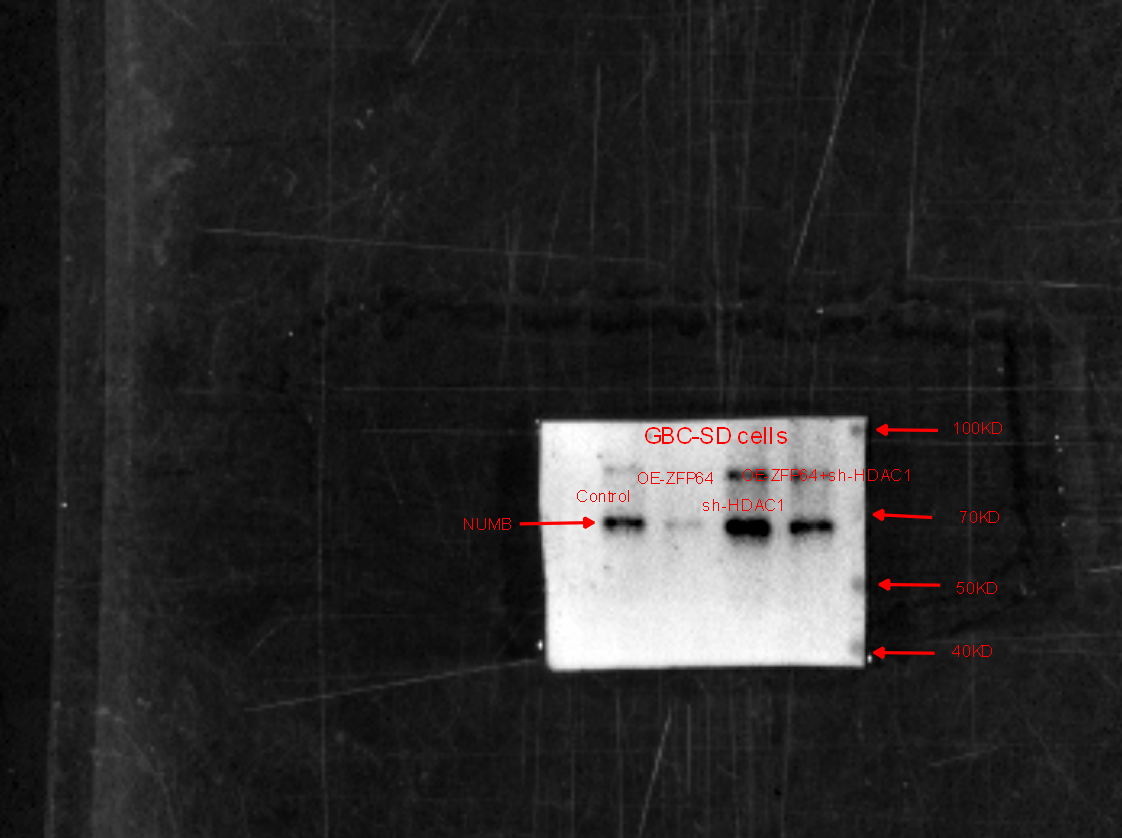

Supplement: Supplementary file 1 [file cancers-15-04508-s001.zip › cancers-2573702-supplementary/Figure S11-Figure 6B/NUMB (GBC-SD).tif]

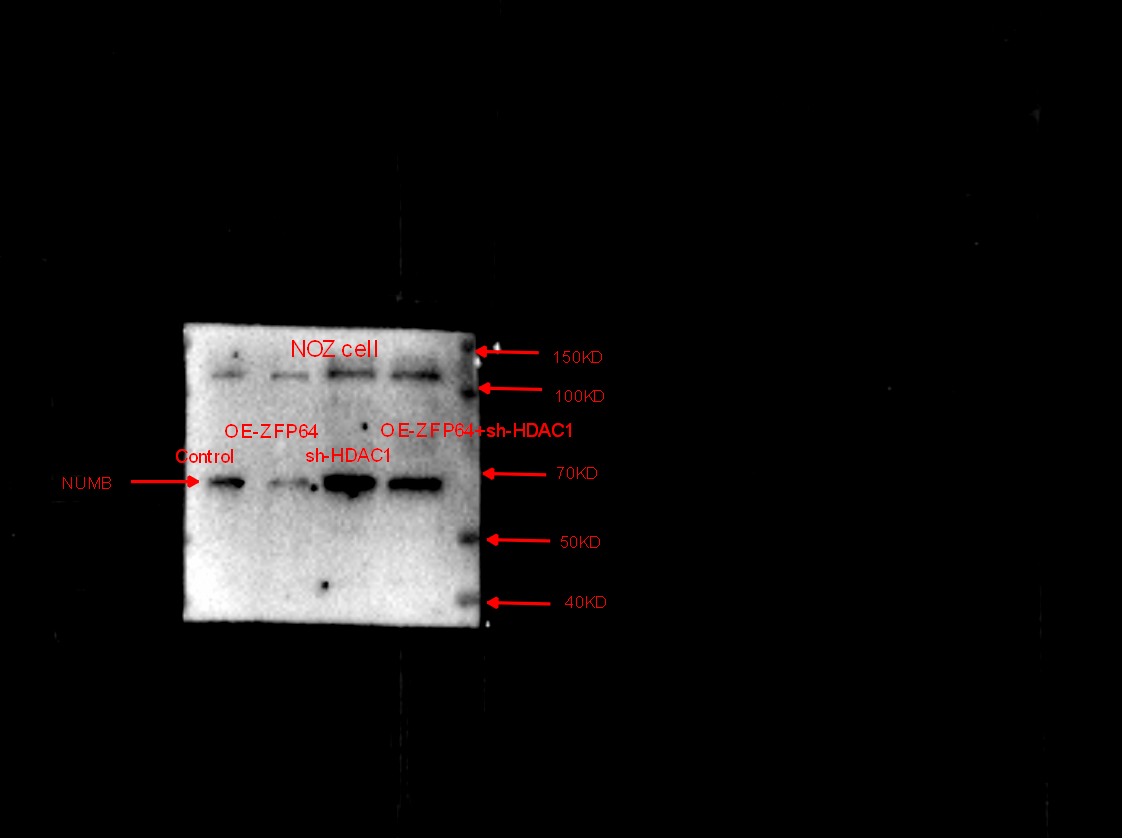

Supplement: Supplementary file 1 [file cancers-15-04508-s001.zip › cancers-2573702-supplementary/Figure S11-Figure 6B/NUMB (NOZ).tif]

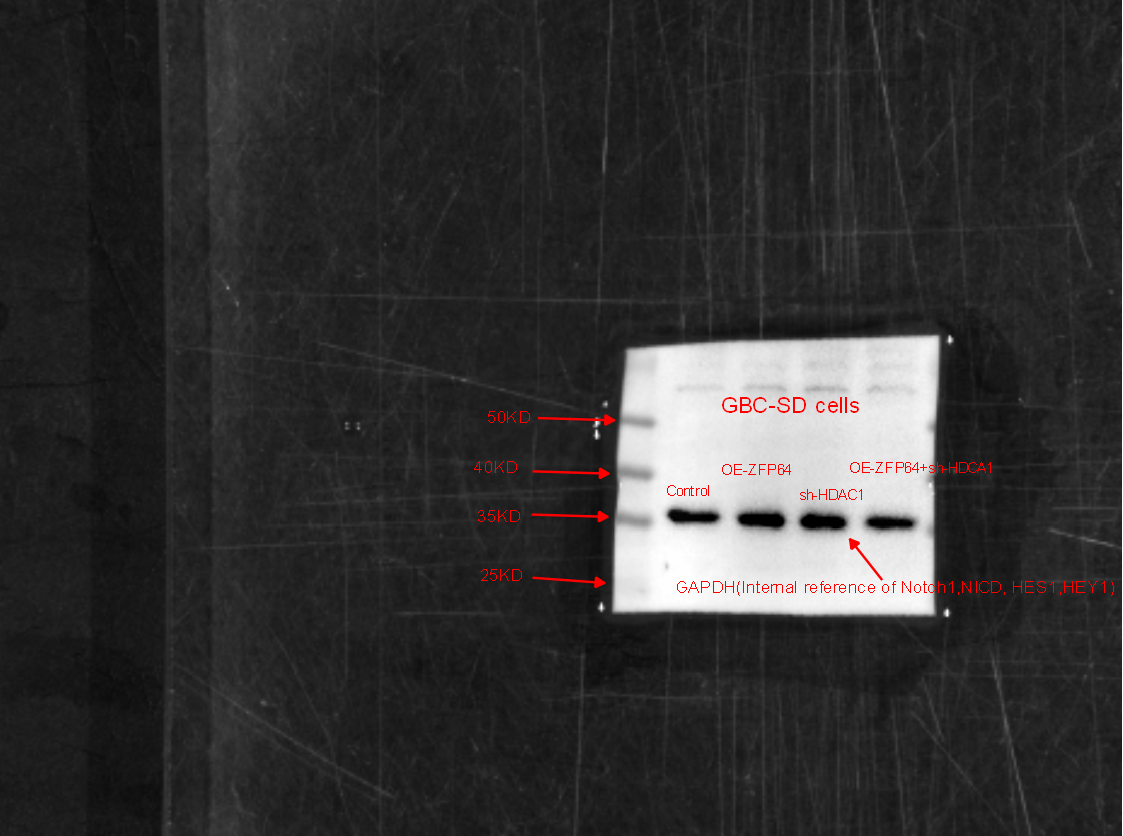

Supplement: Supplementary file 1 [file cancers-15-04508-s001.zip › cancers-2573702-supplementary/Figure S12-Figure 6C/GAPDH (GBC-SD).tif]

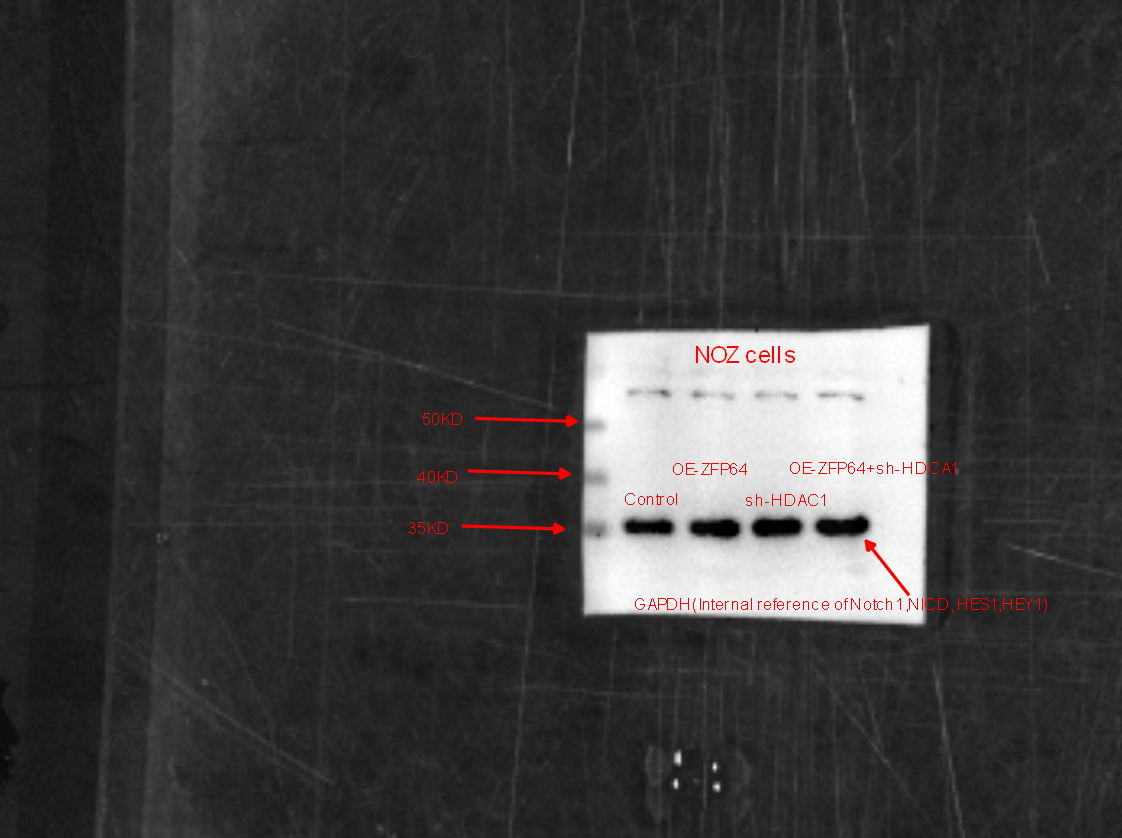

Supplement: Supplementary file 1 [file cancers-15-04508-s001.zip › cancers-2573702-supplementary/Figure S12-Figure 6C/GAPDH (NOZ).tif]

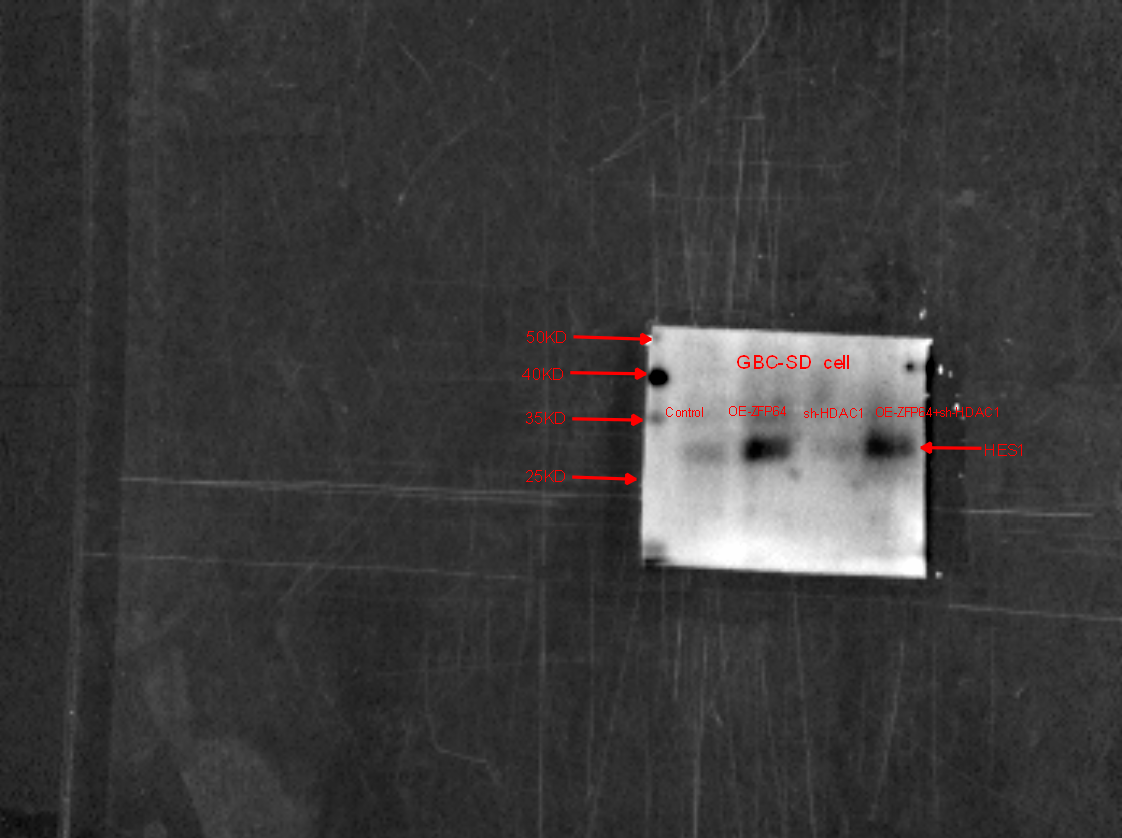

Supplement: Supplementary file 1 [file cancers-15-04508-s001.zip › cancers-2573702-supplementary/Figure S12-Figure 6C/HES1 (GBC-SD).tif]

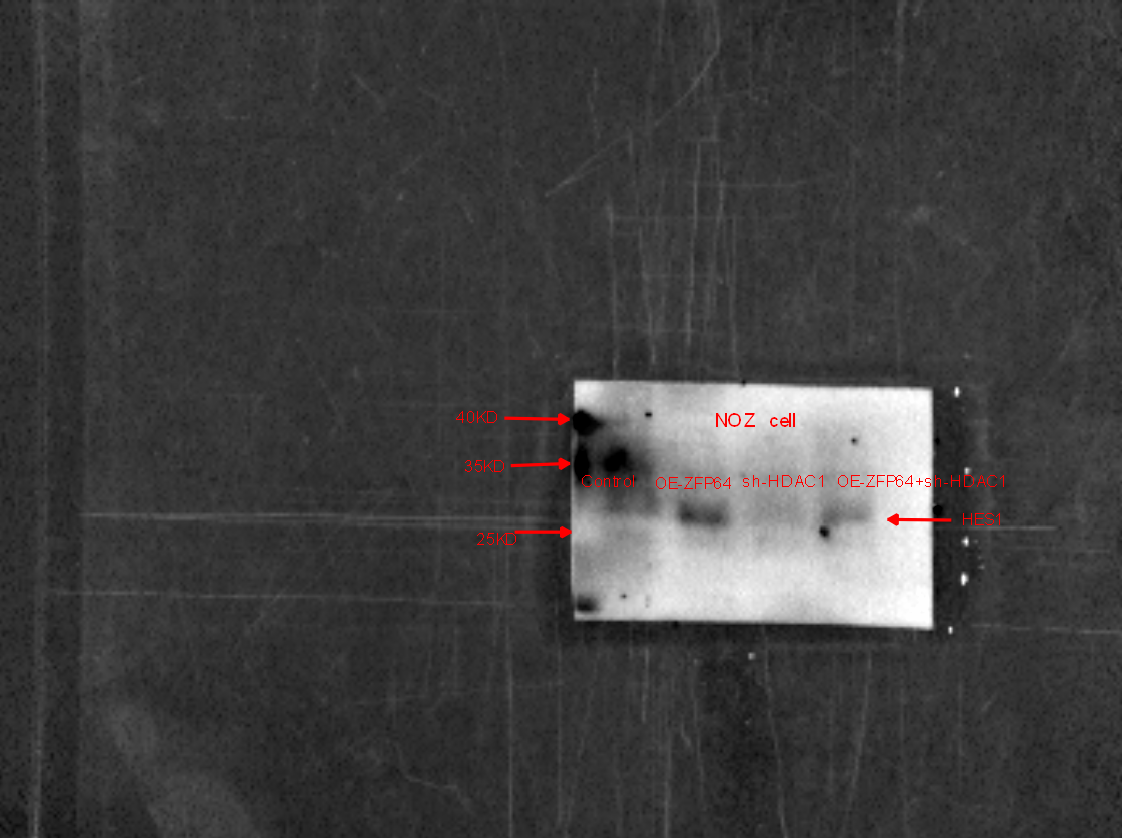

Supplement: Supplementary file 1 [file cancers-15-04508-s001.zip › cancers-2573702-supplementary/Figure S12-Figure 6C/HES1 (NOZ).tif]

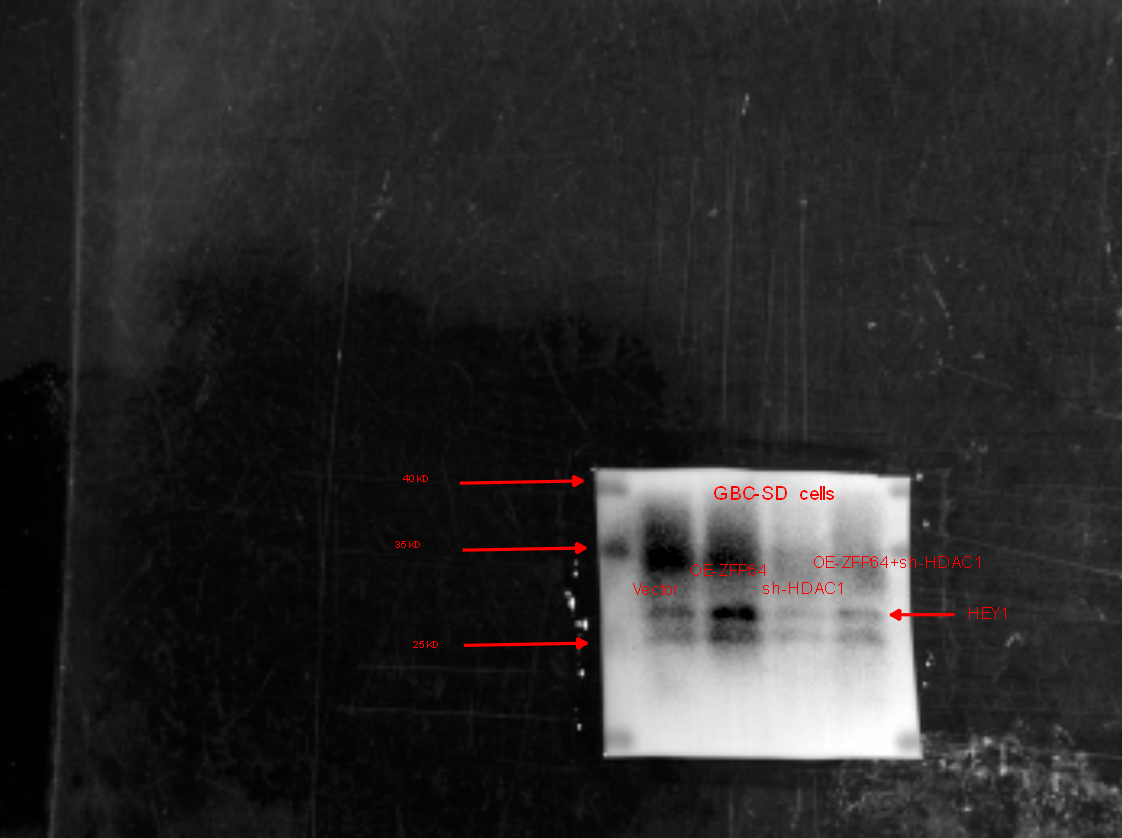

Supplement: Supplementary file 1 [file cancers-15-04508-s001.zip › cancers-2573702-supplementary/Figure S12-Figure 6C/HEY1 (GBC-SD).tif]

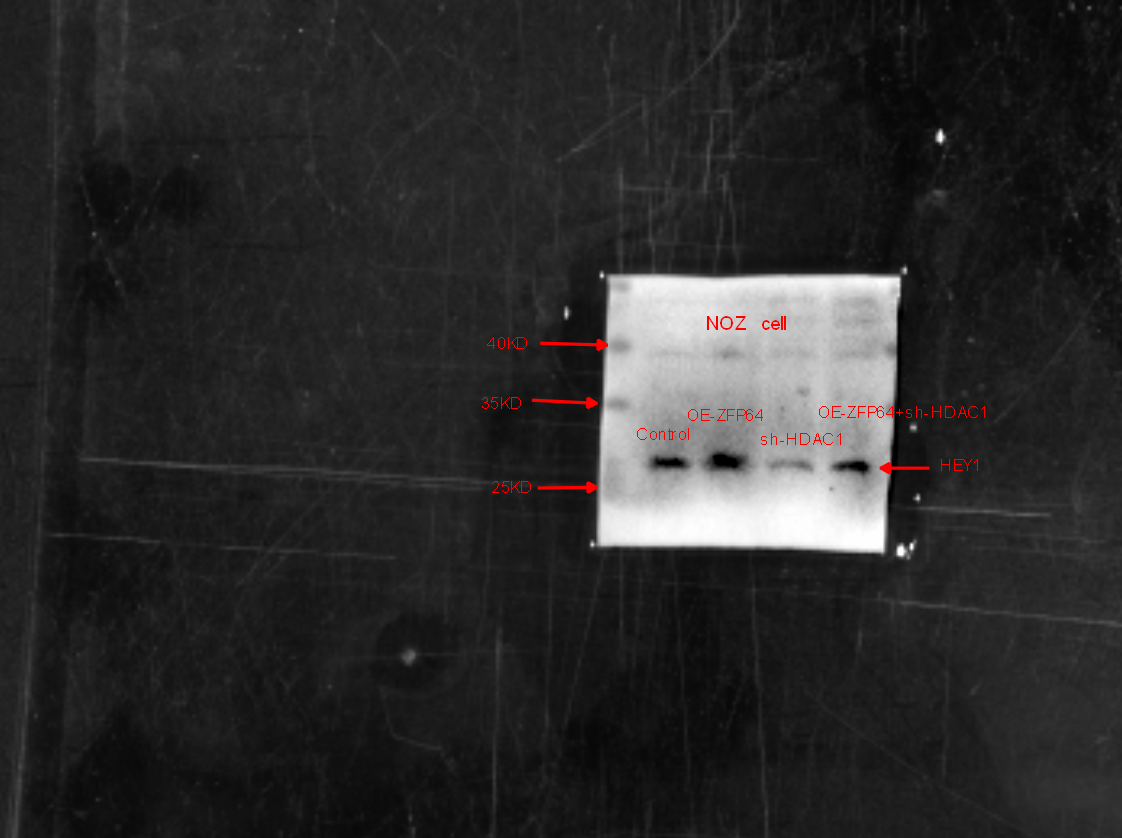

Supplement: Supplementary file 1 [file cancers-15-04508-s001.zip › cancers-2573702-supplementary/Figure S12-Figure 6C/HEY1 (NOZ).tif]

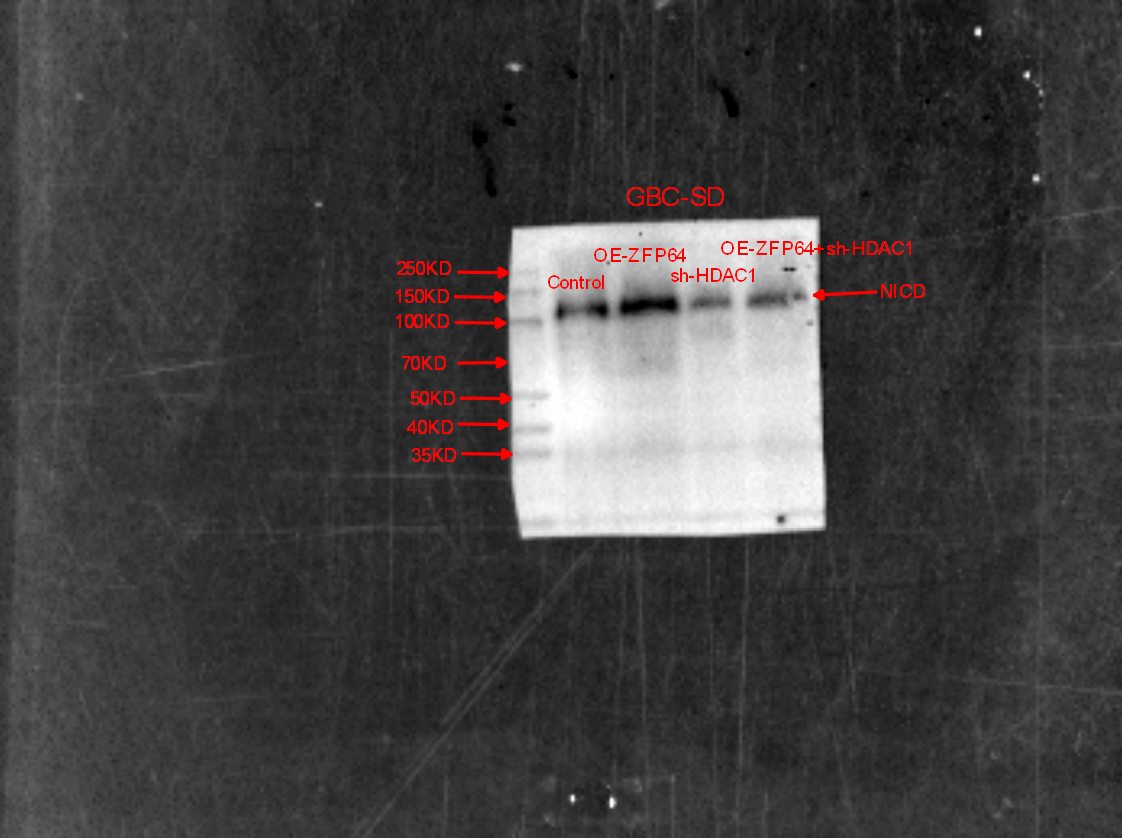

Supplement: Supplementary file 1 [file cancers-15-04508-s001.zip › cancers-2573702-supplementary/Figure S12-Figure 6C/NICD (GBC-SD).tif]

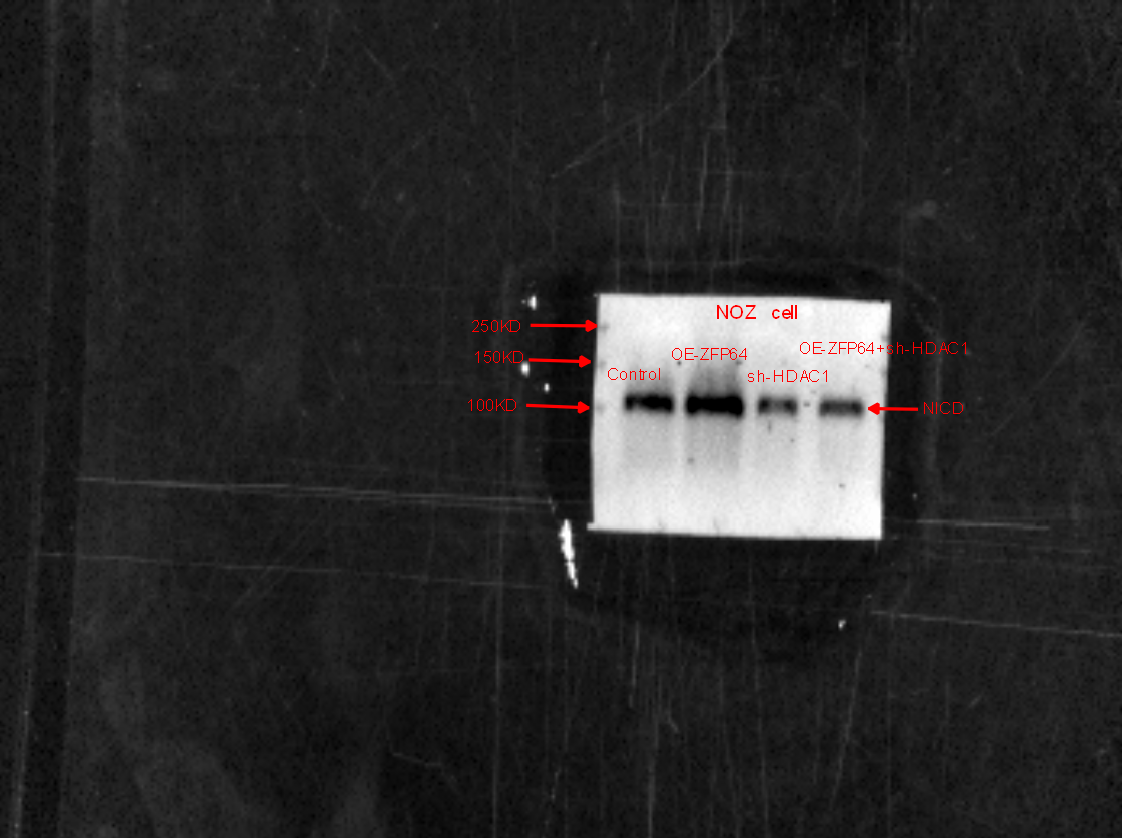

Supplement: Supplementary file 1 [file cancers-15-04508-s001.zip › cancers-2573702-supplementary/Figure S12-Figure 6C/NICD (NOZ).tif]

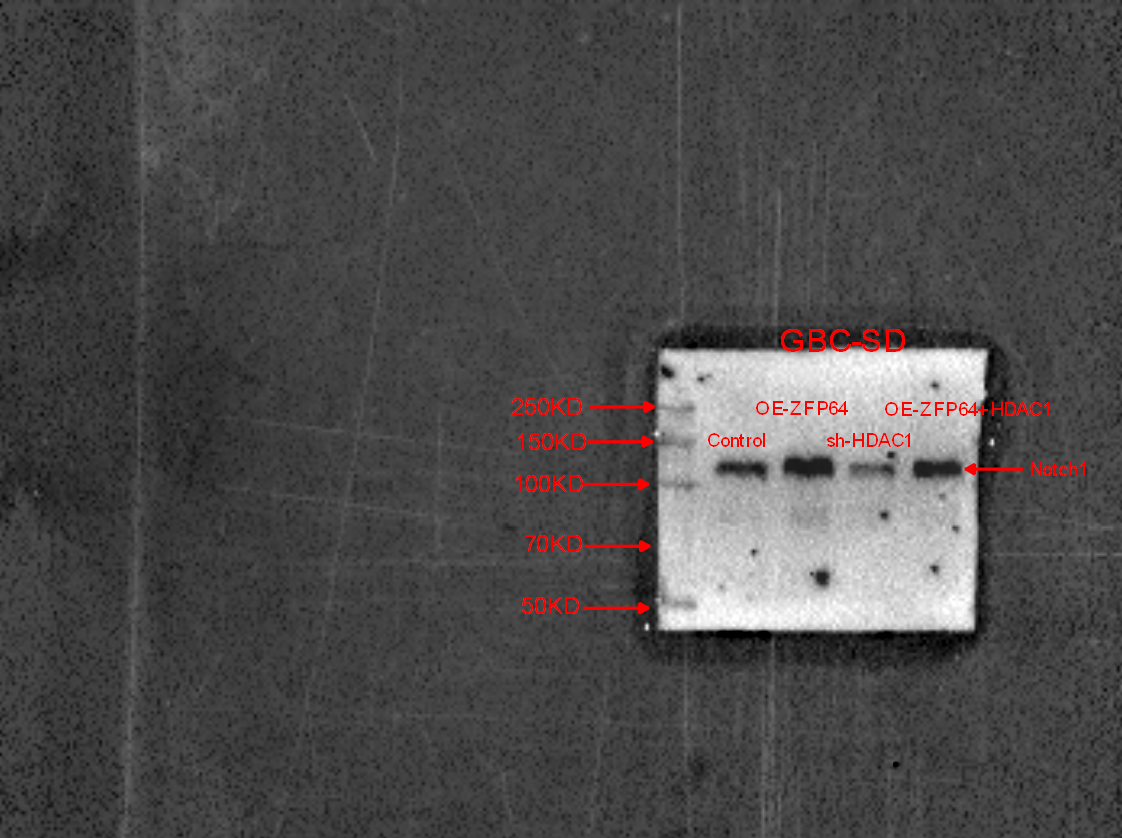

Supplement: Supplementary file 1 [file cancers-15-04508-s001.zip › cancers-2573702-supplementary/Figure S12-Figure 6C/Notch1 (GBC-SD).tif]

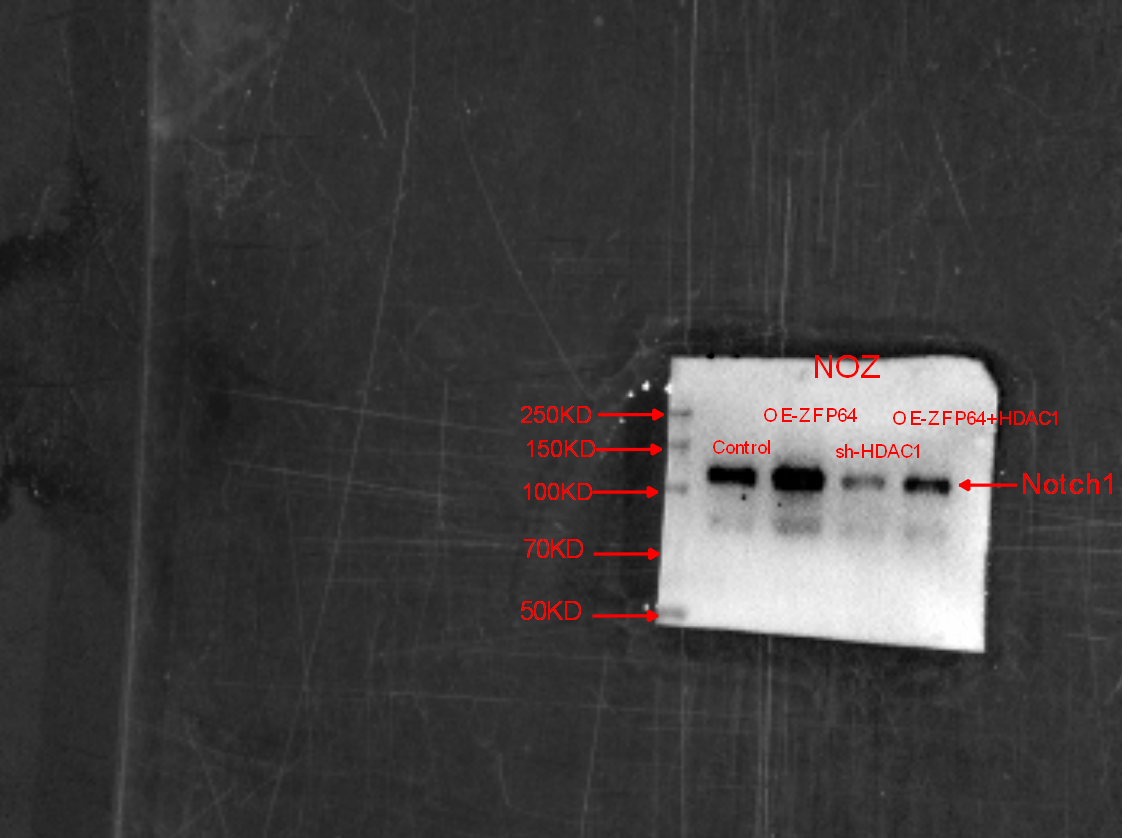

Supplement: Supplementary file 1 [file cancers-15-04508-s001.zip › cancers-2573702-supplementary/Figure S12-Figure 6C/Notch1 (NOZ).tif]

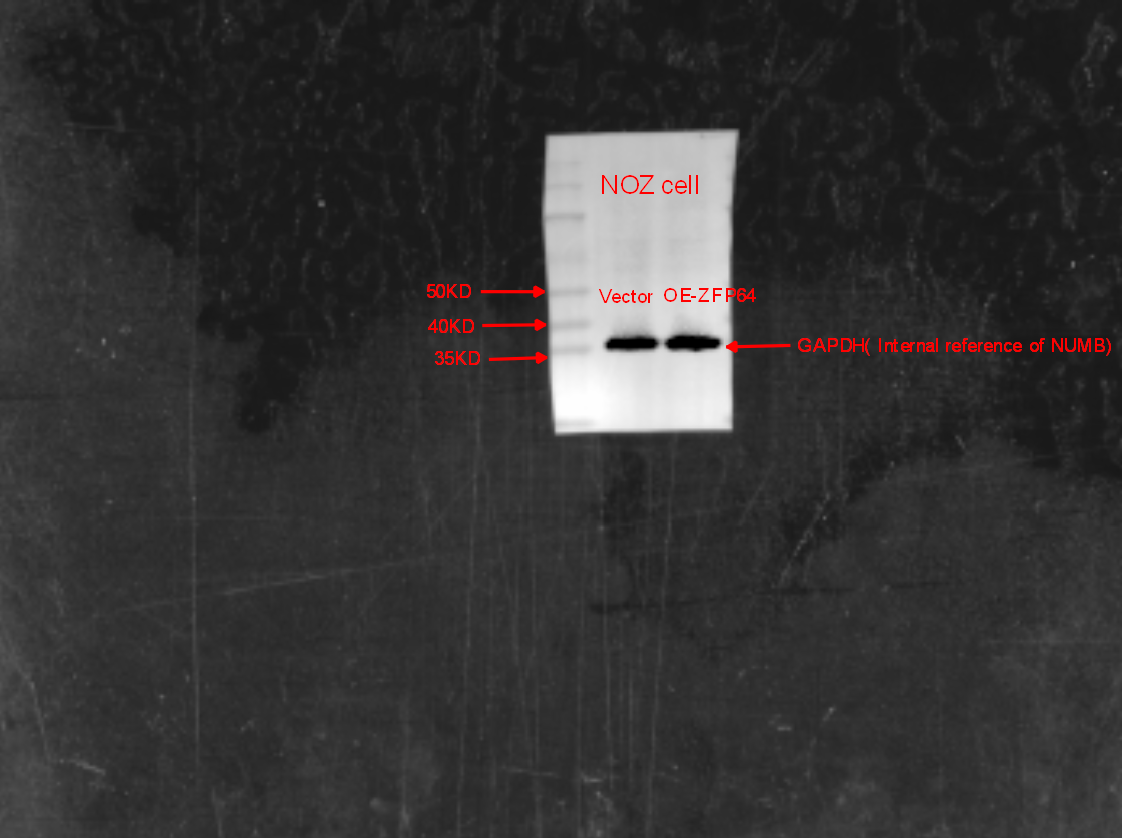

Supplement: Supplementary file 1 [file cancers-15-04508-s001.zip › cancers-2573702-supplementary/Figure S13-Figure S1C/GAPDH (NOZ).tif]

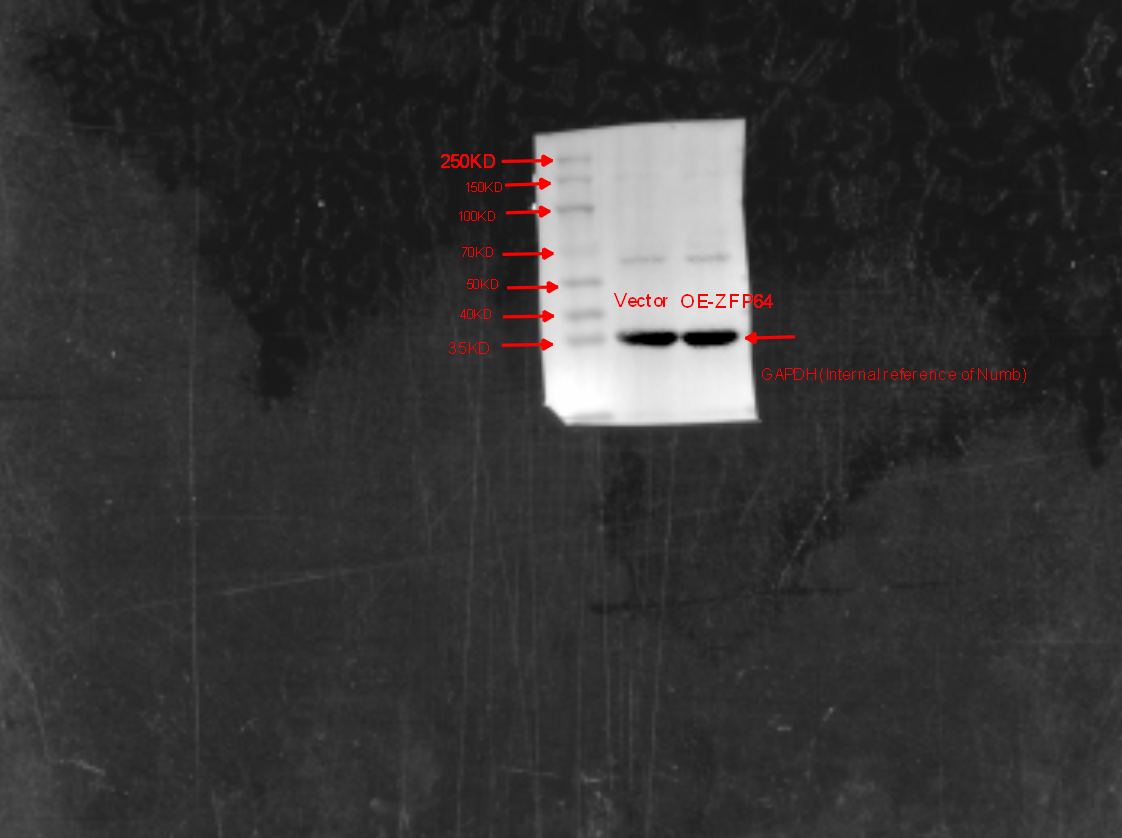

Supplement: Supplementary file 1 [file cancers-15-04508-s001.zip › cancers-2573702-supplementary/Figure S13-Figure S1C/GAPDH (GBC-SD).tif]

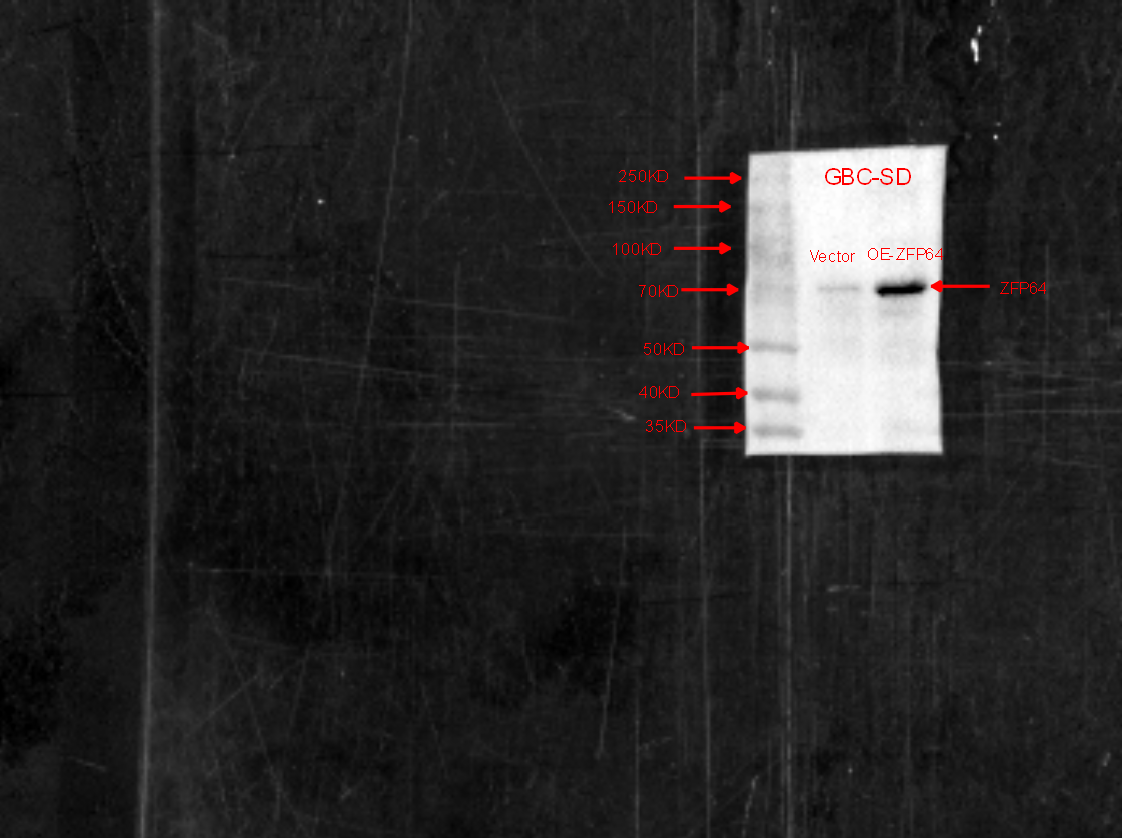

Supplement: Supplementary file 1 [file cancers-15-04508-s001.zip › cancers-2573702-supplementary/Figure S13-Figure S1C/ZFP64 (GBC-SD) (2).tif]

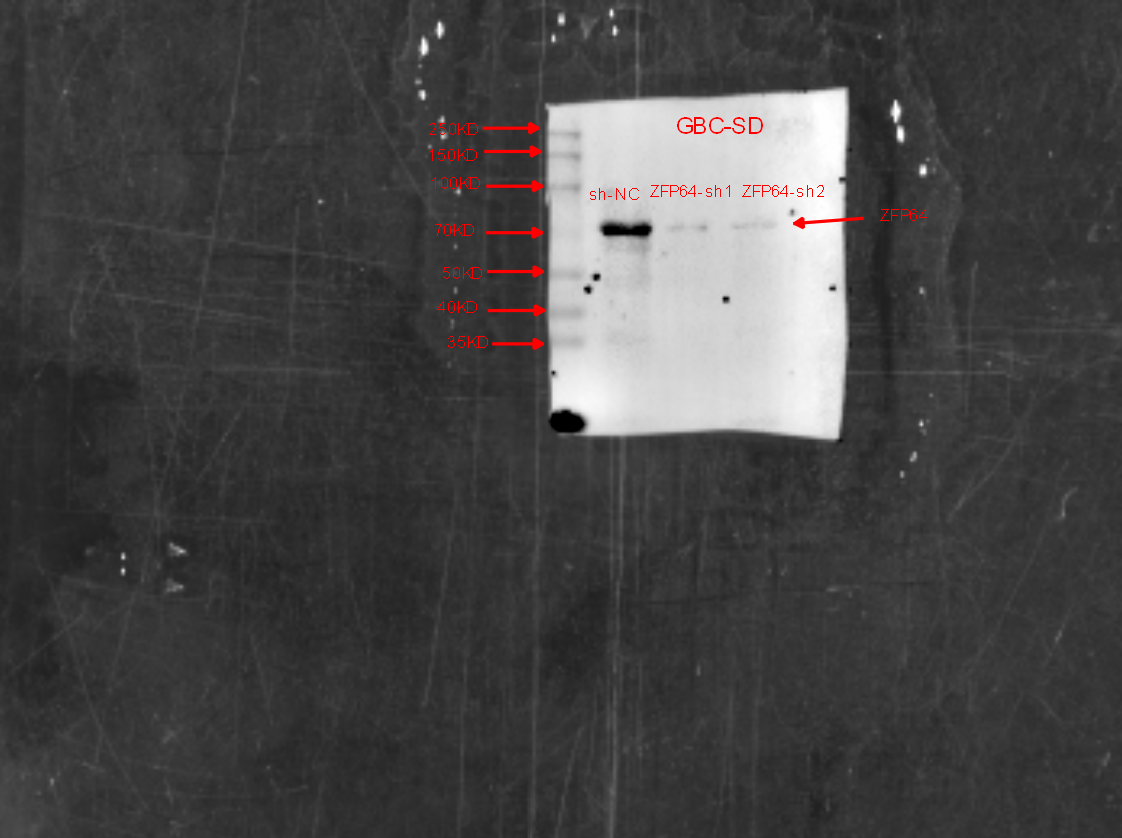

Supplement: Supplementary file 1 [file cancers-15-04508-s001.zip › cancers-2573702-supplementary/Figure S13-Figure S1C/ZFP64 (GBC-SD).tif]

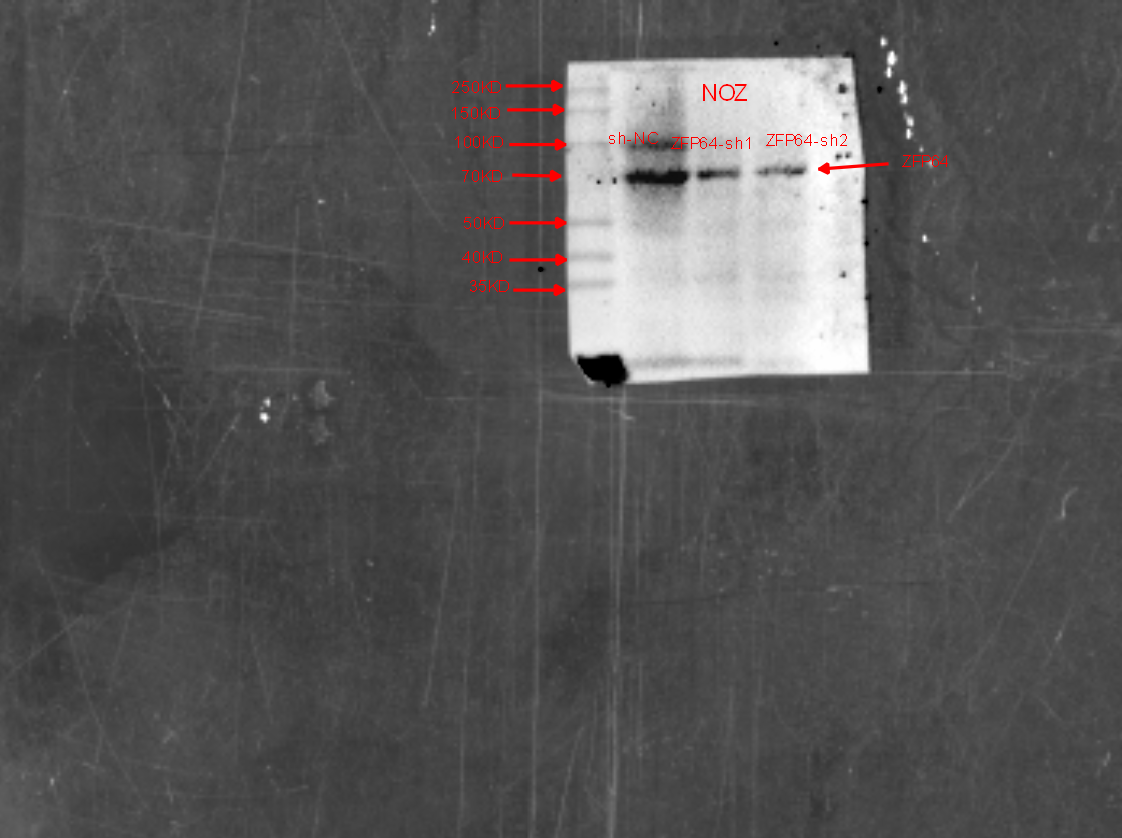

Supplement: Supplementary file 1 [file cancers-15-04508-s001.zip › cancers-2573702-supplementary/Figure S13-Figure S1C/ZFP64 (NOZ) (2).tif]

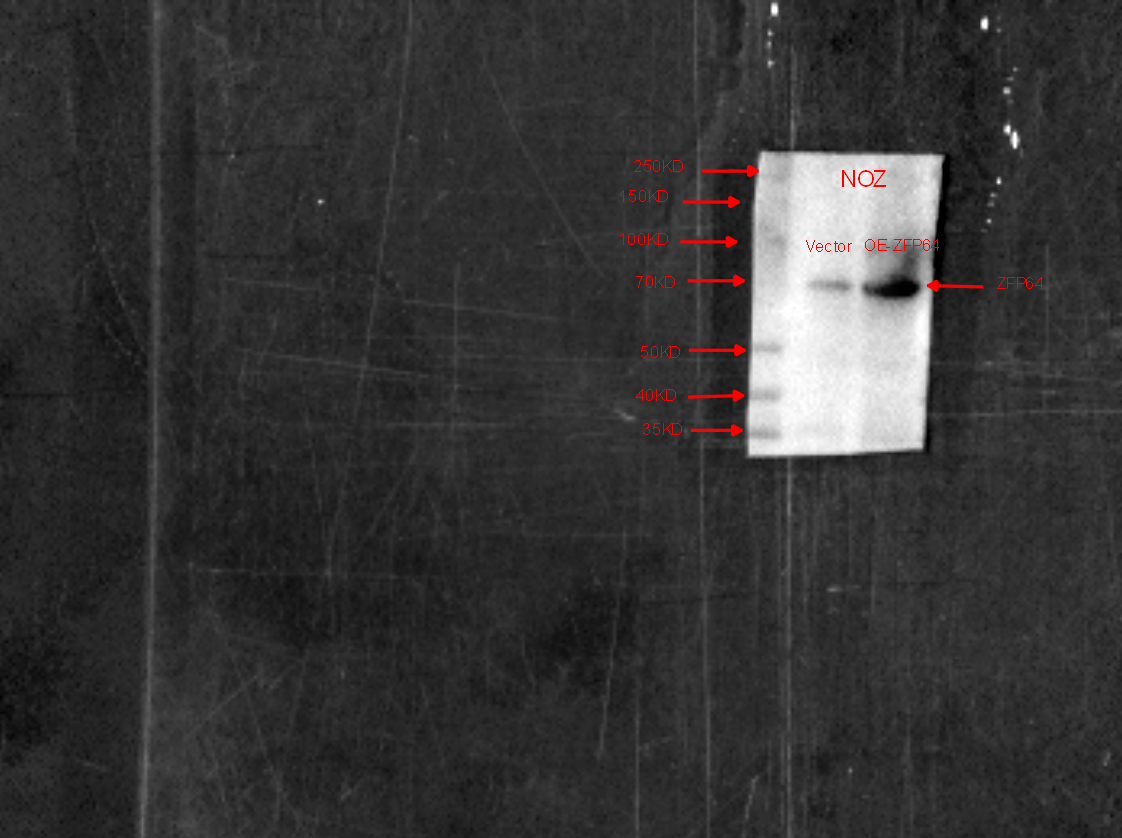

Supplement: Supplementary file 1 [file cancers-15-04508-s001.zip › cancers-2573702-supplementary/Figure S13-Figure S1C/ZFP64 (NOZ).tif]

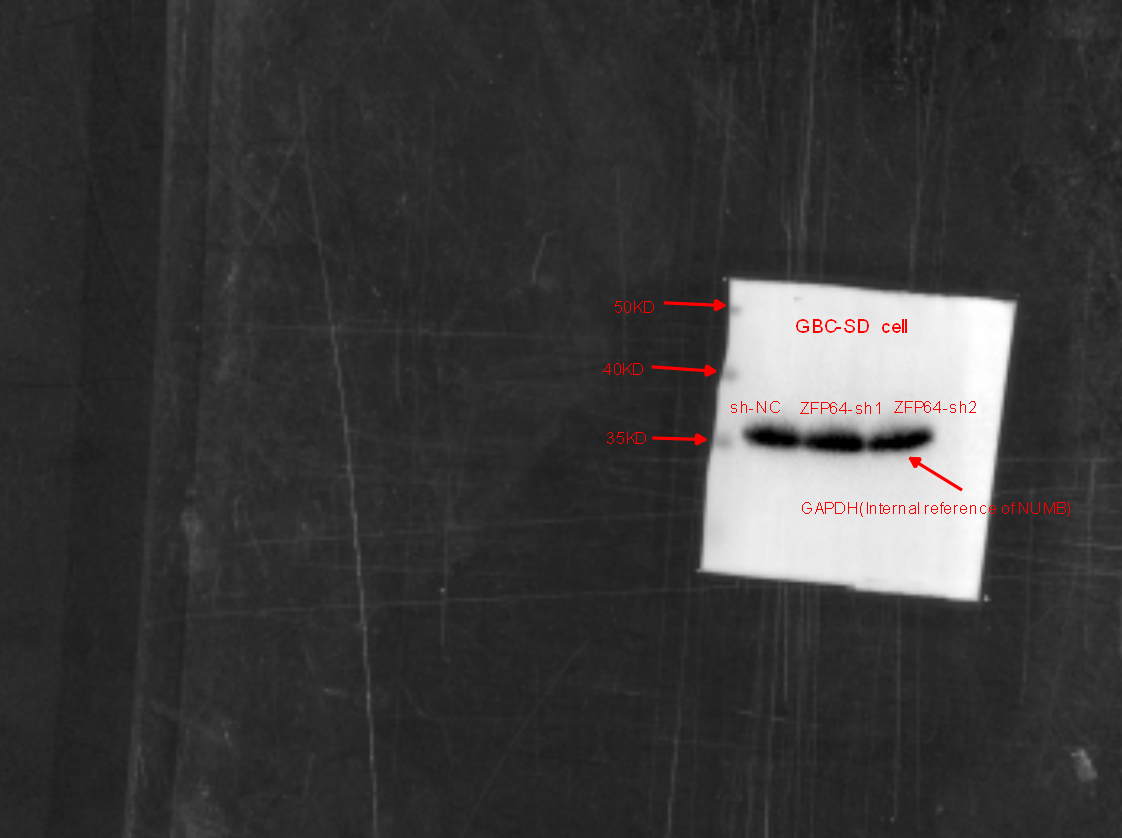

Supplement: Supplementary file 1 [file cancers-15-04508-s001.zip › cancers-2573702-supplementary/Figure S14-Figure 5E/GAPDH (GBC-SD).tif]

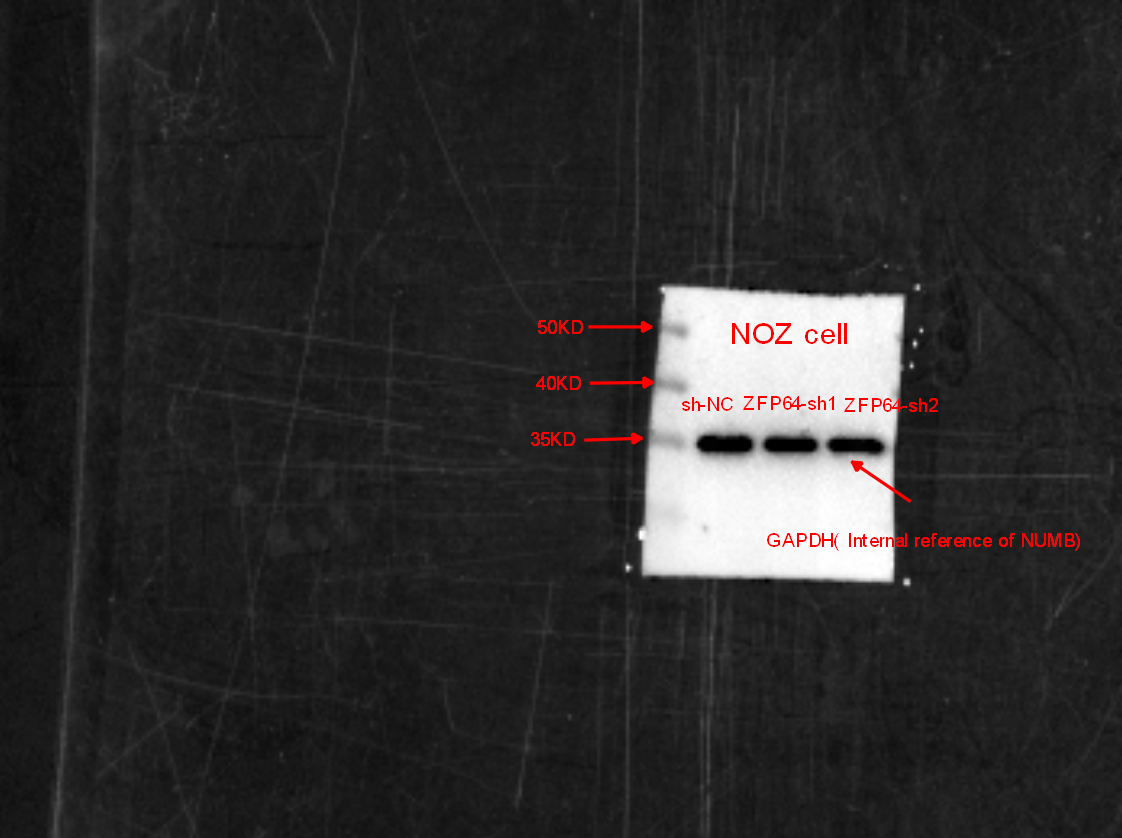

Supplement: Supplementary file 1 [file cancers-15-04508-s001.zip › cancers-2573702-supplementary/Figure S14-Figure 5E/GAPDH (NOZ).tif]

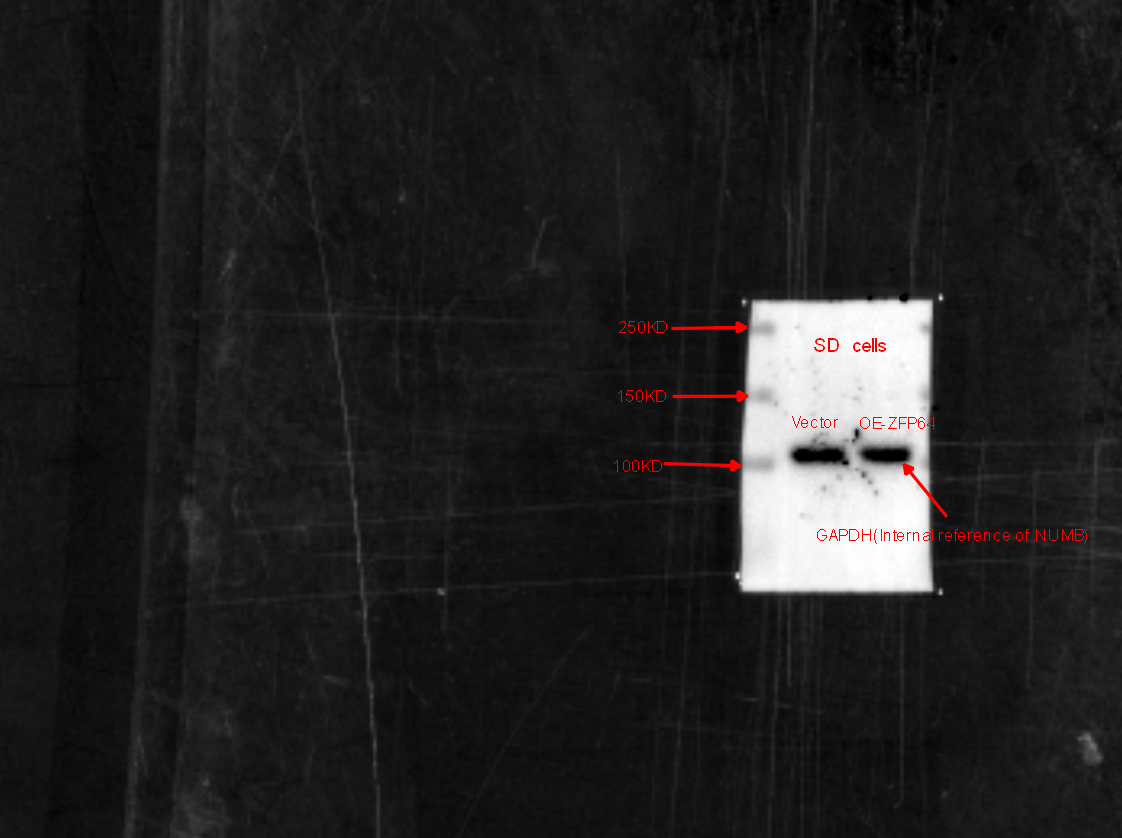

Supplement: Supplementary file 1 [file cancers-15-04508-s001.zip › cancers-2573702-supplementary/Figure S14-Figure 5E/GAPDH-2 (GBC-SD).tif]

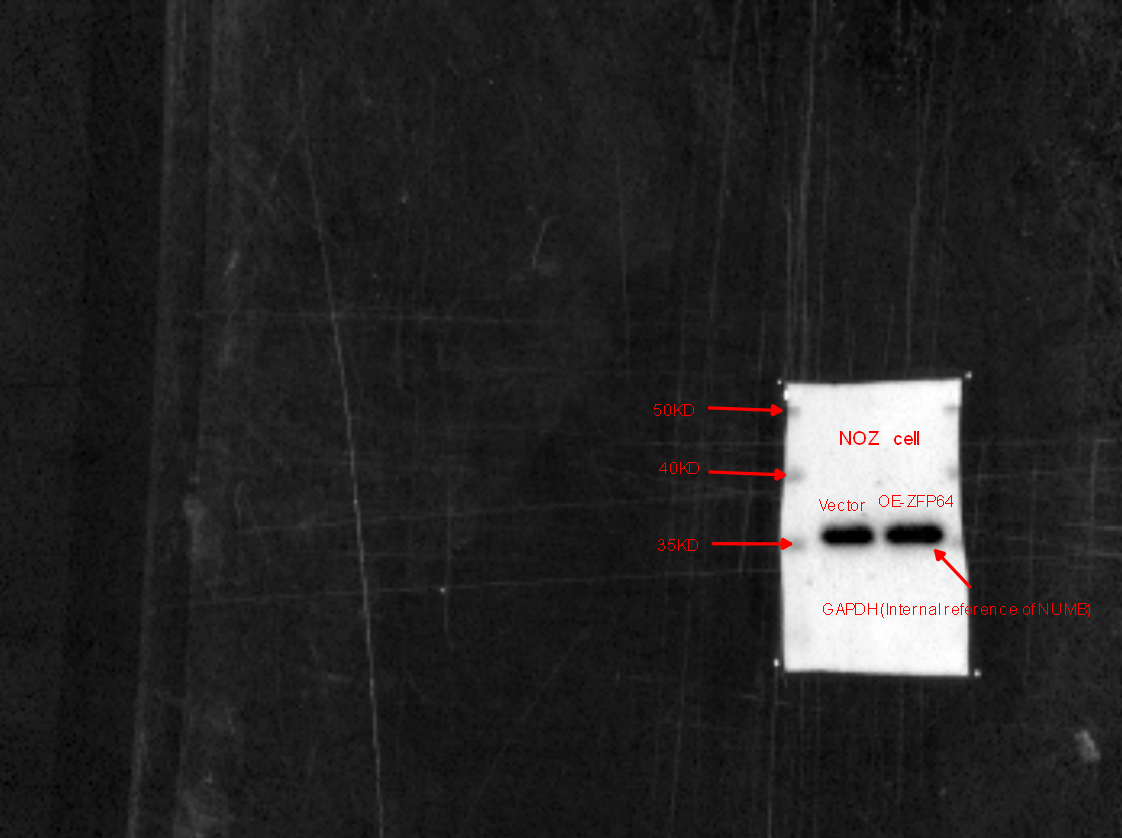

Supplement: Supplementary file 1 [file cancers-15-04508-s001.zip › cancers-2573702-supplementary/Figure S14-Figure 5E/GAPDH-2 (NOZ).tif]

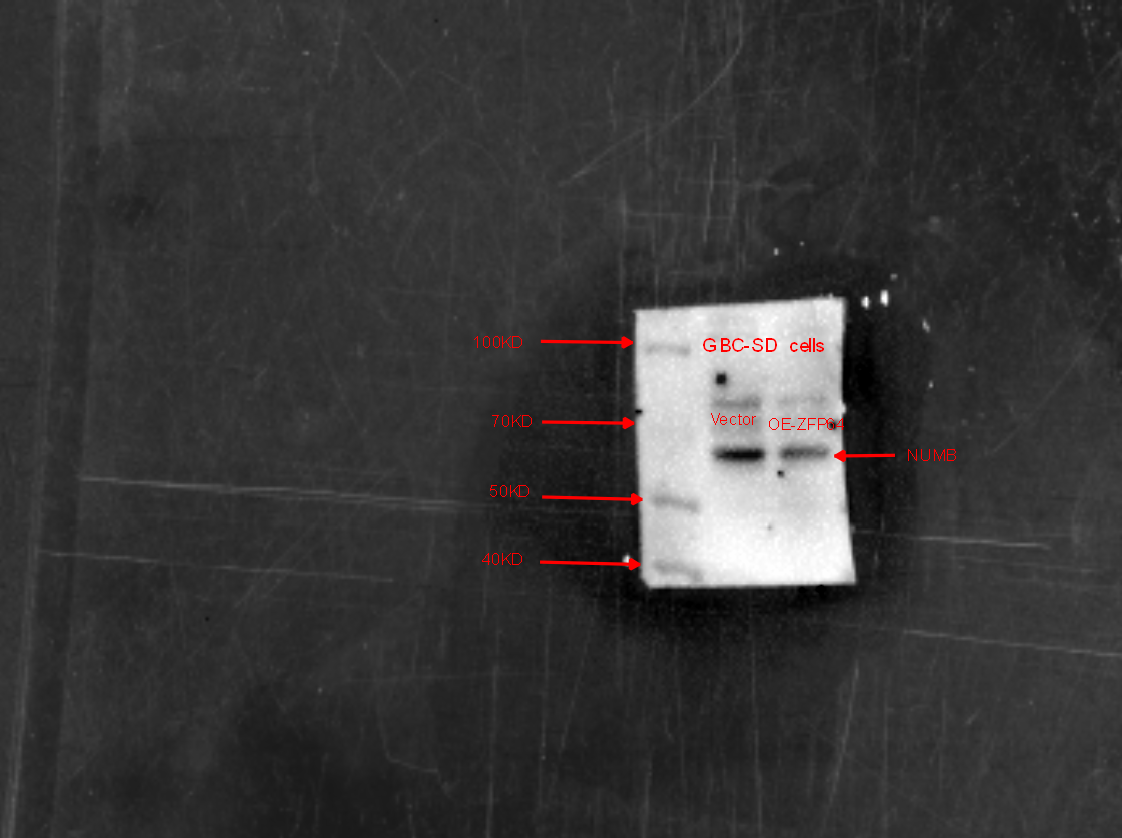

Supplement: Supplementary file 1 [file cancers-15-04508-s001.zip › cancers-2573702-supplementary/Figure S14-Figure 5E/NUMB (GBC-SD).tif]

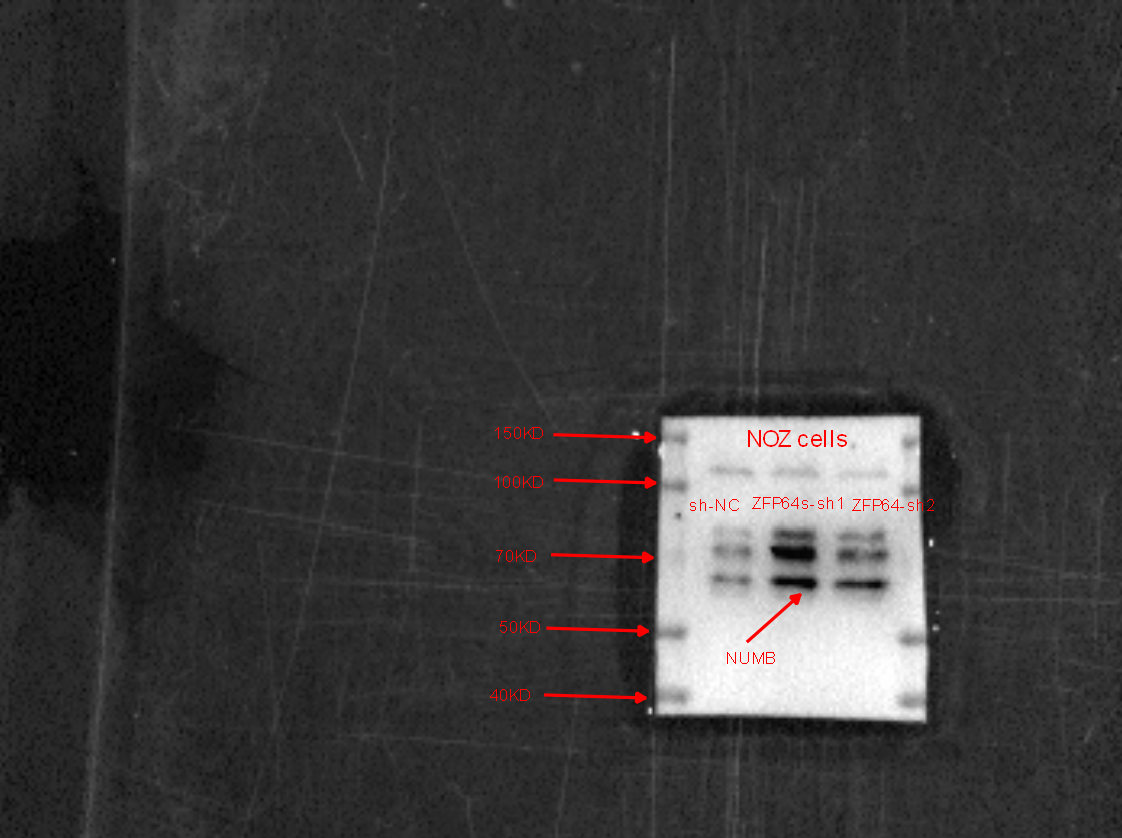

Supplement: Supplementary file 1 [file cancers-15-04508-s001.zip › cancers-2573702-supplementary/Figure S14-Figure 5E/NUMB (NOZ).tif]

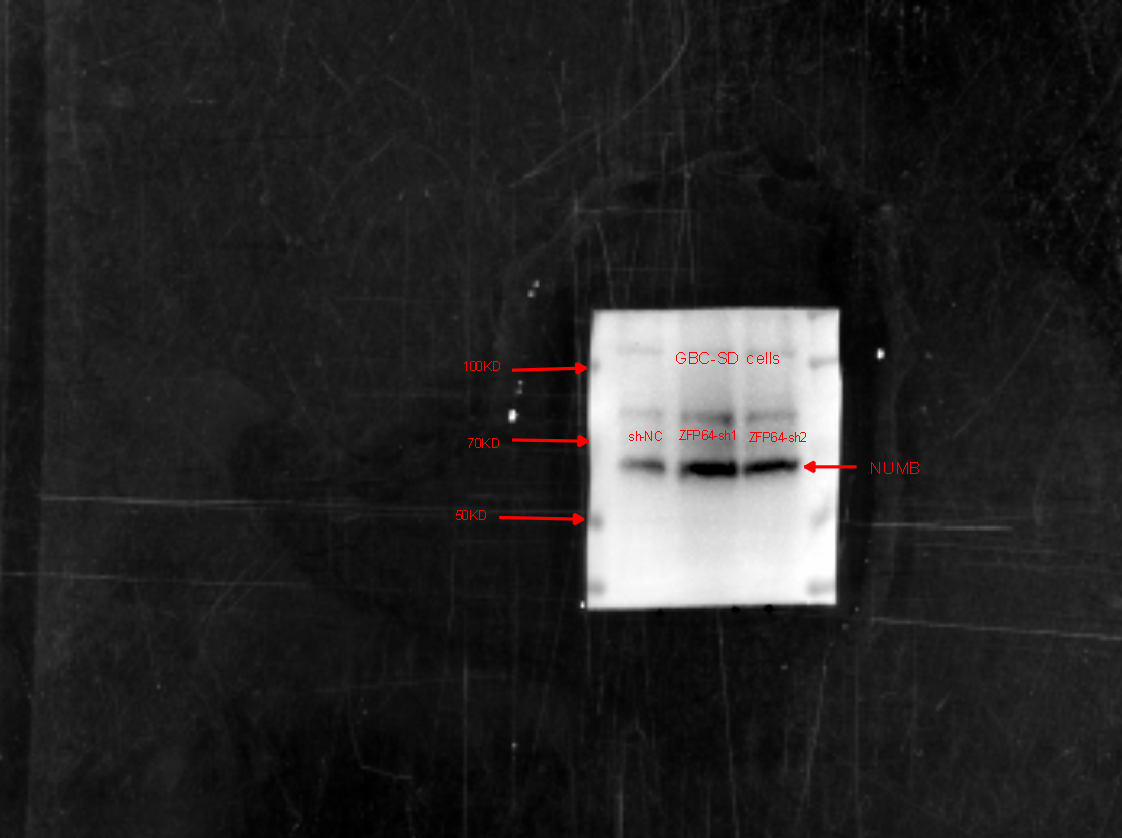

Supplement: Supplementary file 1 [file cancers-15-04508-s001.zip › cancers-2573702-supplementary/Figure S14-Figure 5E/NUMB-2 (GBC-SD).tif]

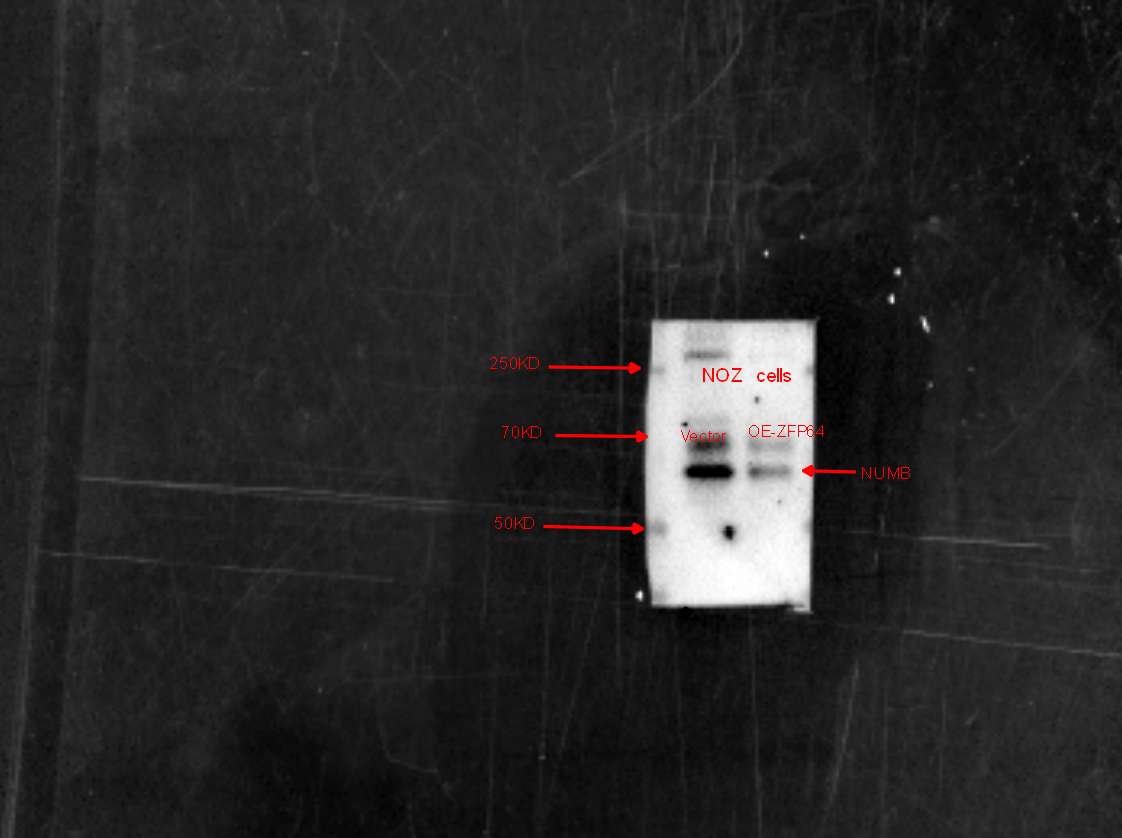

Supplement: Supplementary file 1 [file cancers-15-04508-s001.zip › cancers-2573702-supplementary/Figure S14-Figure 5E/NUMB-2 (NOZ).tif]

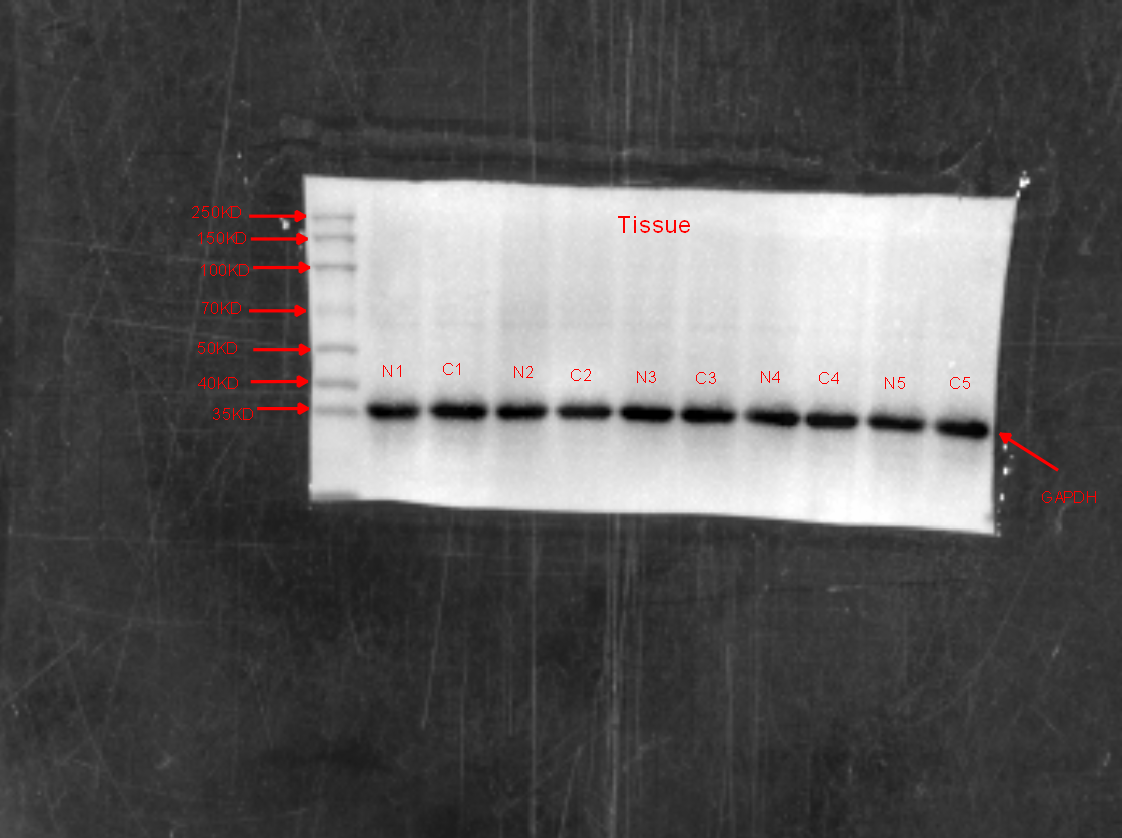

Supplement: Supplementary file 1 [file cancers-15-04508-s001.zip › cancers-2573702-supplementary/Figure S2-Figure 1D/GAPDH(1).tif]

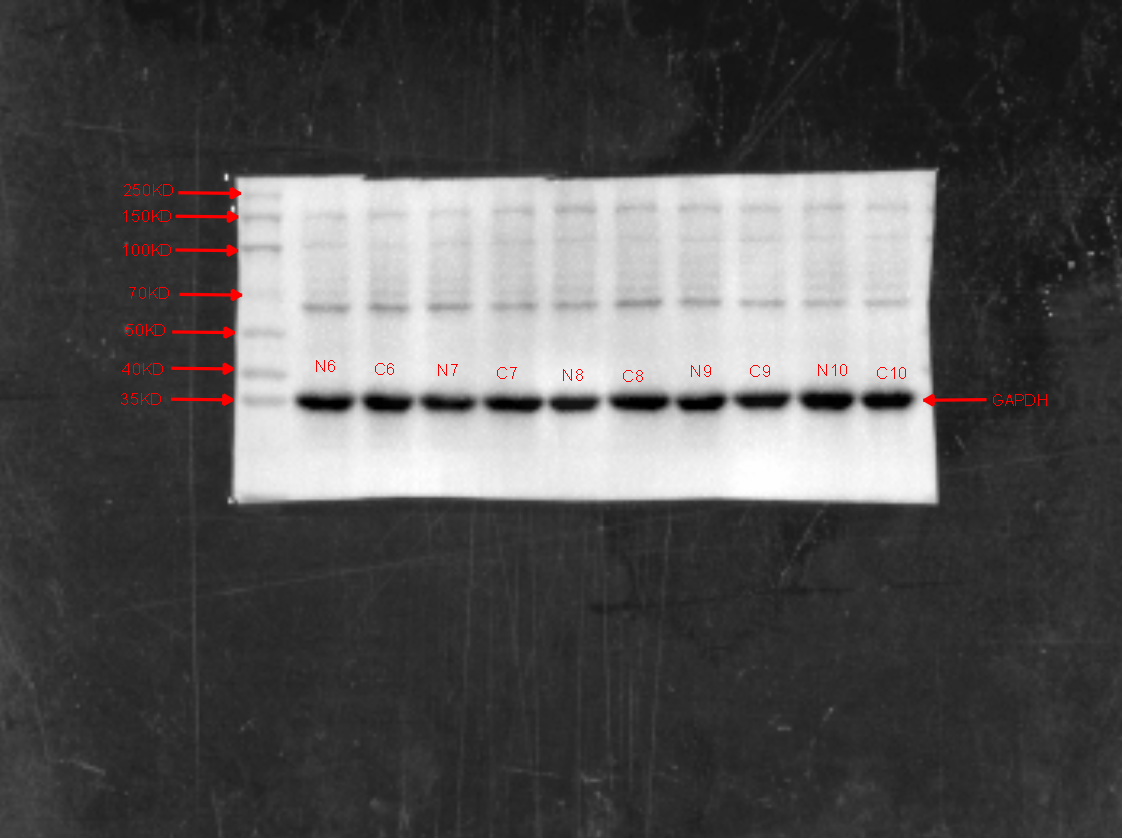

Supplement: Supplementary file 1 [file cancers-15-04508-s001.zip › cancers-2573702-supplementary/Figure S2-Figure 1D/GAPDH(2).tif]

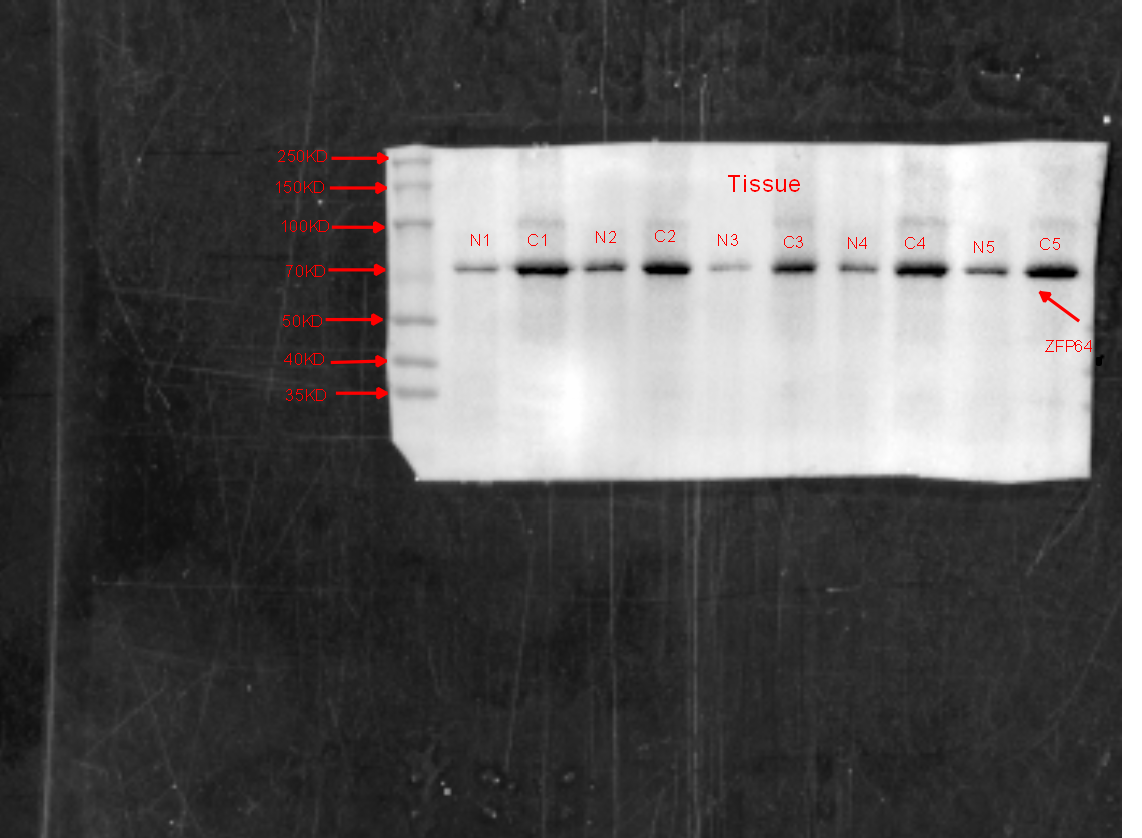

Supplement: Supplementary file 1 [file cancers-15-04508-s001.zip › cancers-2573702-supplementary/Figure S2-Figure 1D/ZFP64(1).tif]

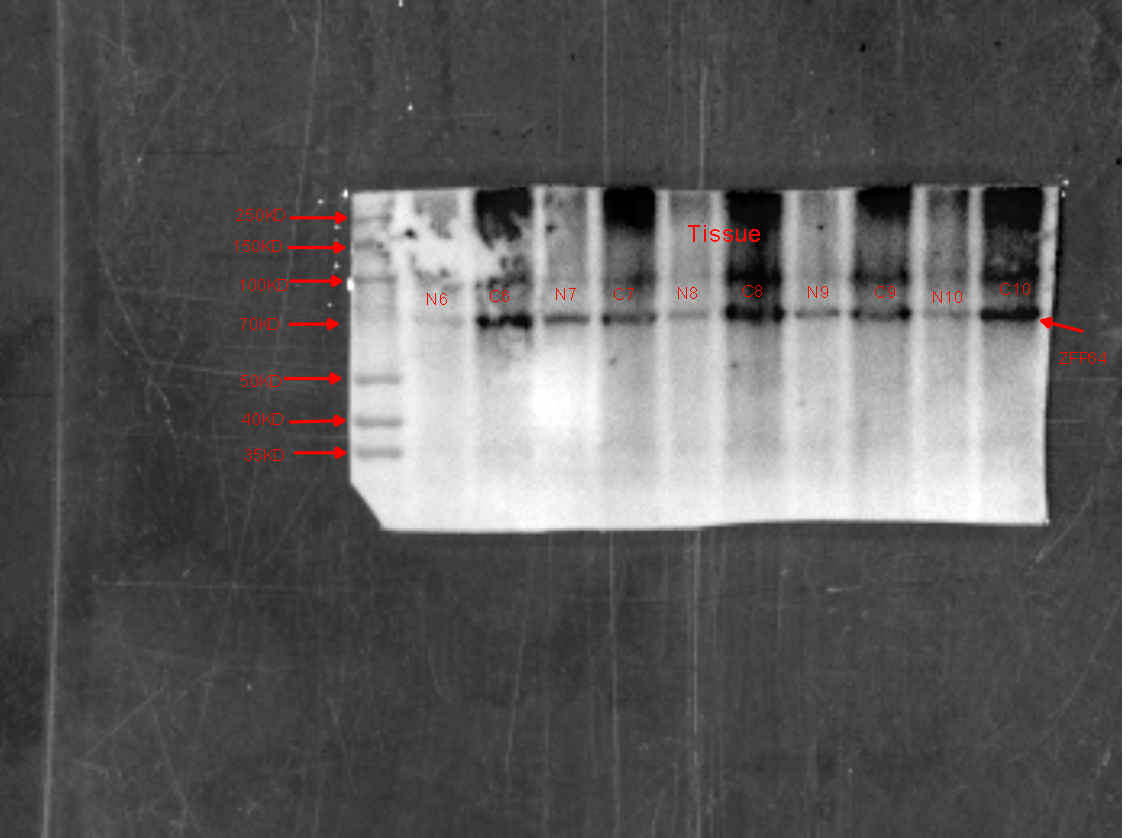

Supplement: Supplementary file 1 [file cancers-15-04508-s001.zip › cancers-2573702-supplementary/Figure S2-Figure 1D/ZFP64(2).tif]

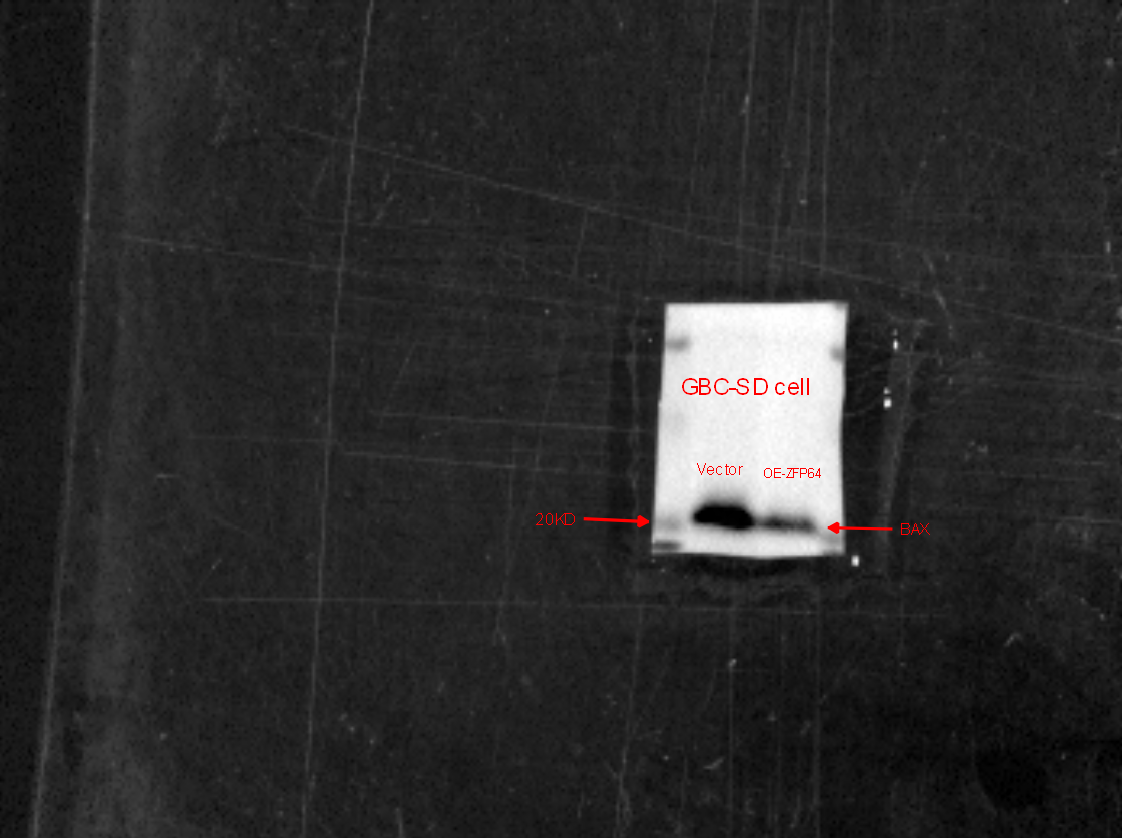

Supplement: Supplementary file 1 [file cancers-15-04508-s001.zip › cancers-2573702-supplementary/Figure S3-Figure 2E/BAX (GBC-SD).tif]

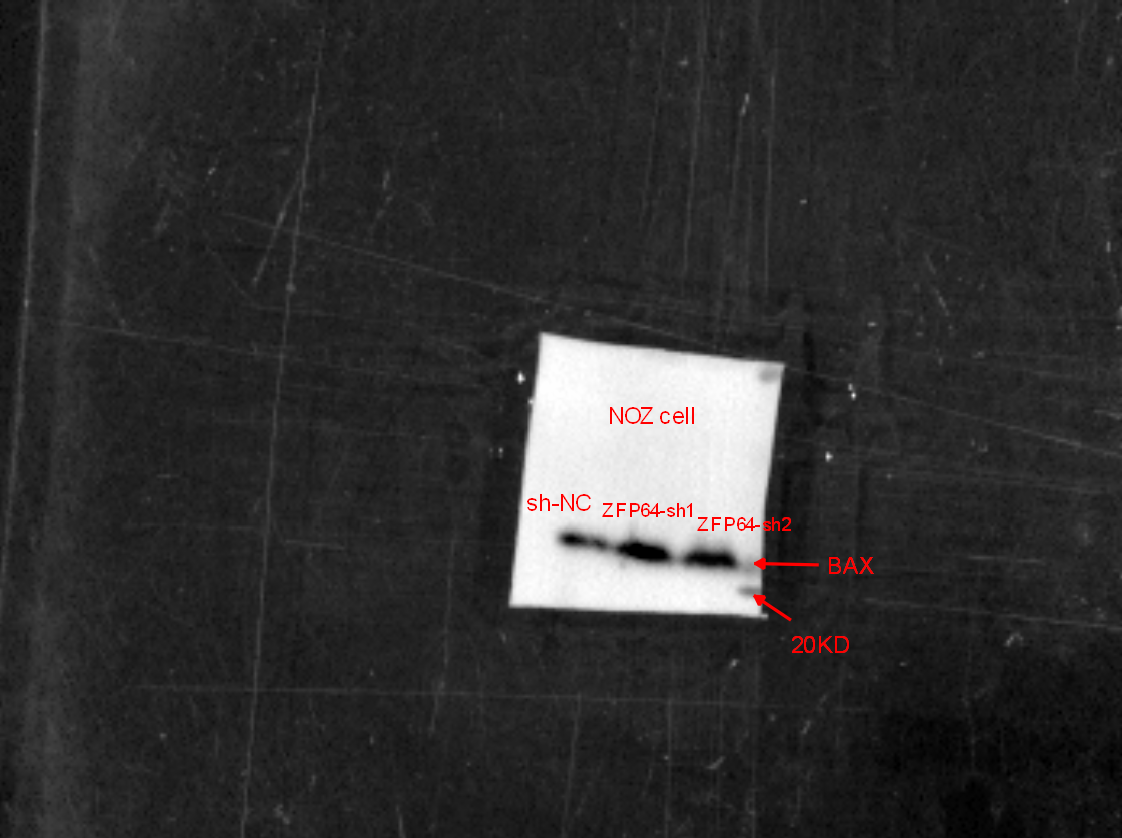

Supplement: Supplementary file 1 [file cancers-15-04508-s001.zip › cancers-2573702-supplementary/Figure S3-Figure 2E/BAX(NOZ).tif]

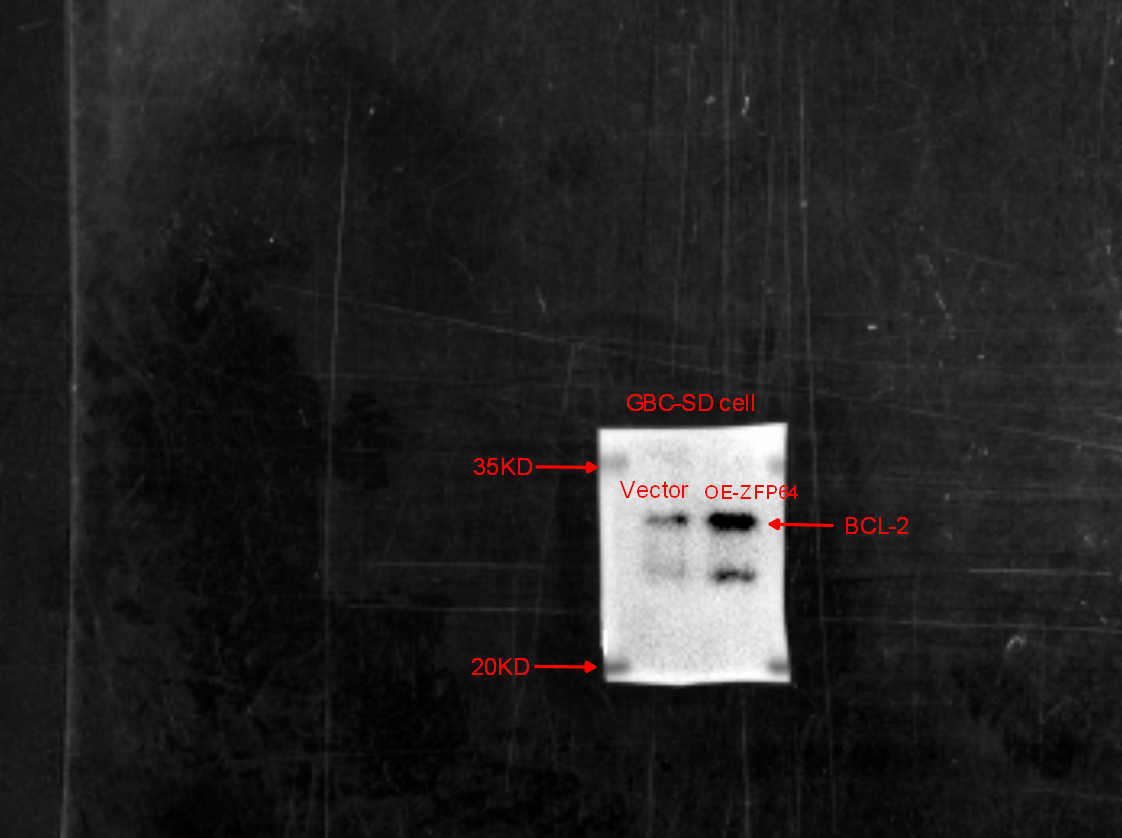

Supplement: Supplementary file 1 [file cancers-15-04508-s001.zip › cancers-2573702-supplementary/Figure S3-Figure 2E/BCL-2(GBC-SD).tif]

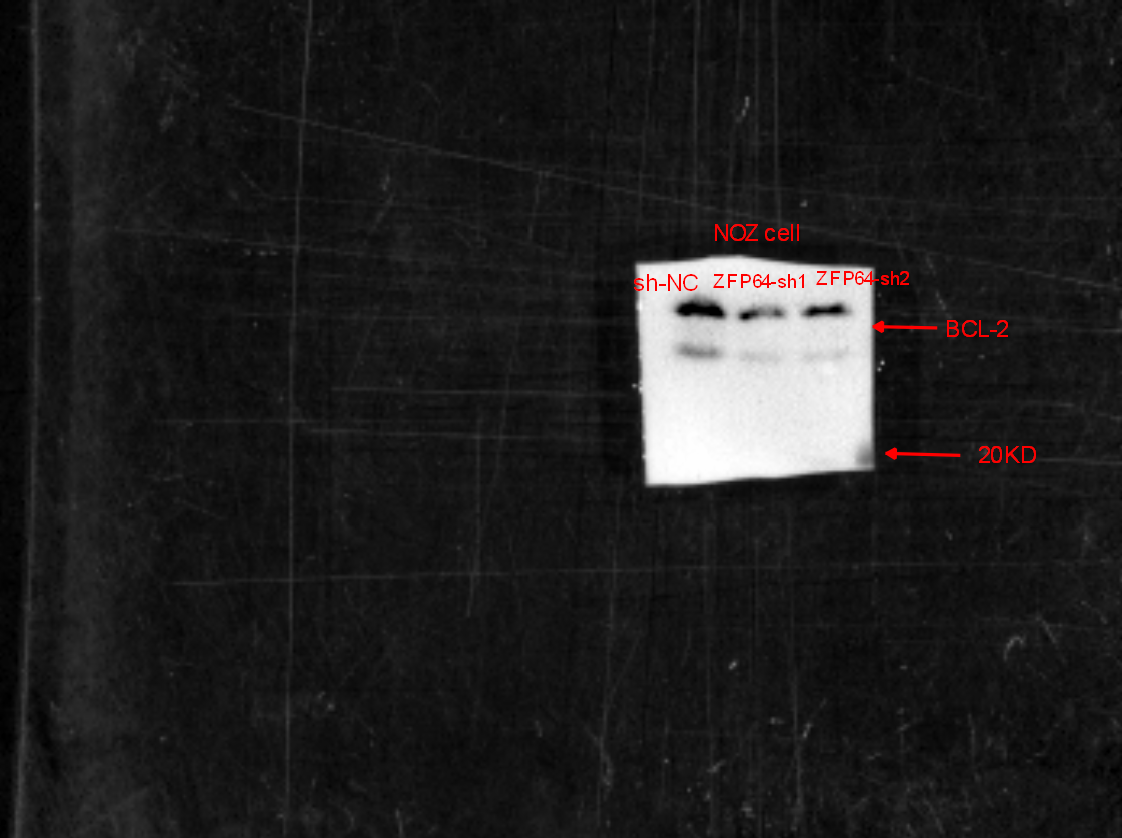

Supplement: Supplementary file 1 [file cancers-15-04508-s001.zip › cancers-2573702-supplementary/Figure S3-Figure 2E/BCL-2(NOZ).tif]

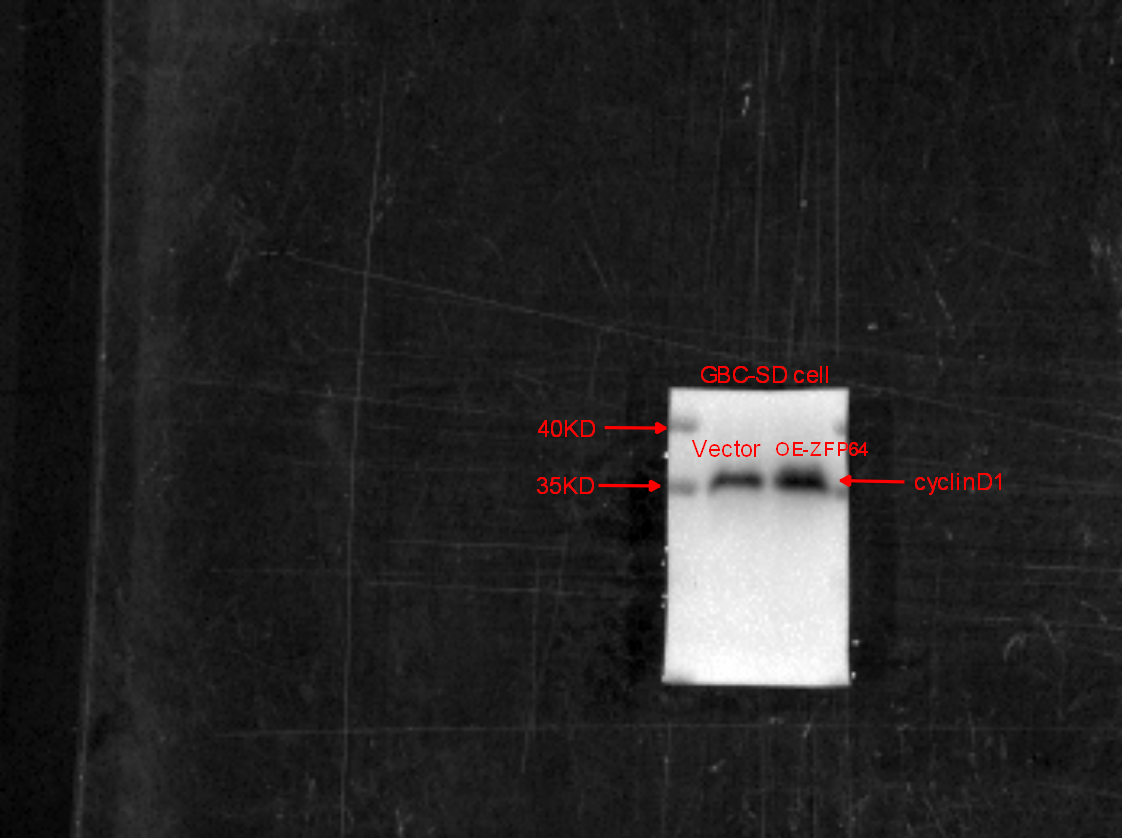

Supplement: Supplementary file 1 [file cancers-15-04508-s001.zip › cancers-2573702-supplementary/Figure S3-Figure 2E/cyclinD1(GBC-SD).tif]

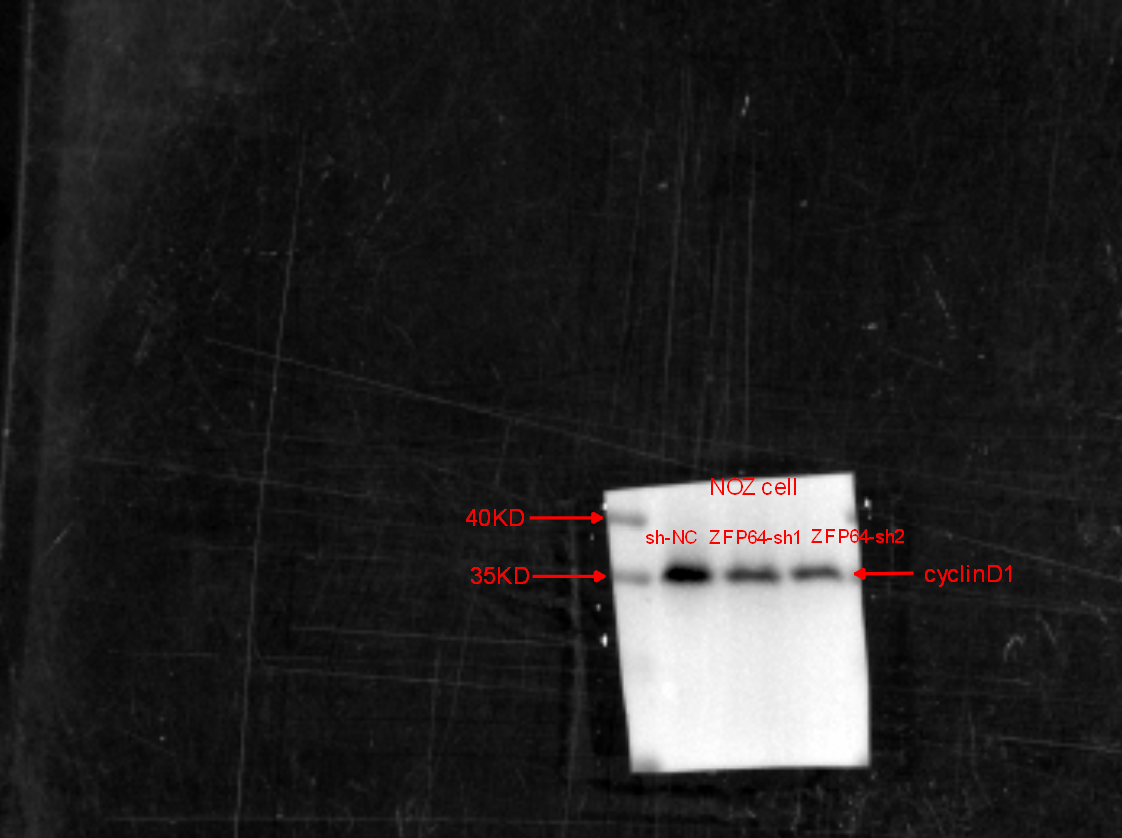

Supplement: Supplementary file 1 [file cancers-15-04508-s001.zip › cancers-2573702-supplementary/Figure S3-Figure 2E/cyclinD1(NOZ).tif]

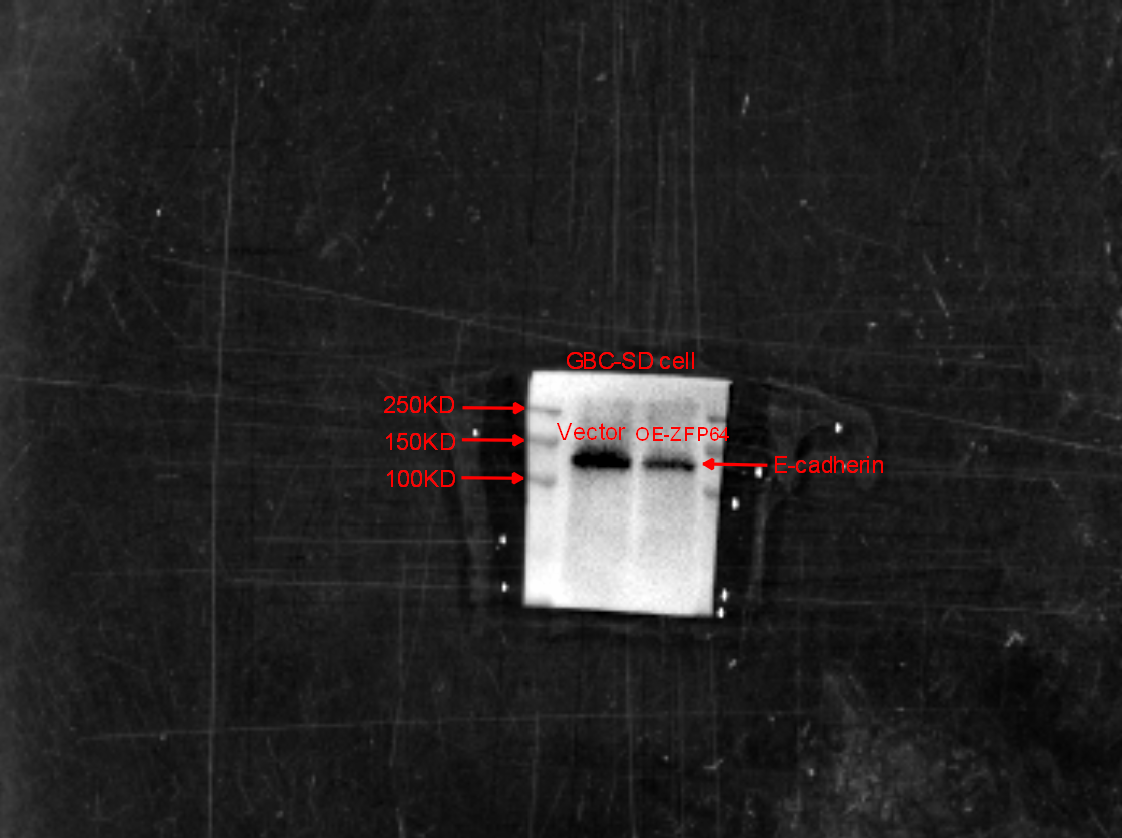

Supplement: Supplementary file 1 [file cancers-15-04508-s001.zip › cancers-2573702-supplementary/Figure S3-Figure 2E/E-cadherin (GBC-SD).tif]

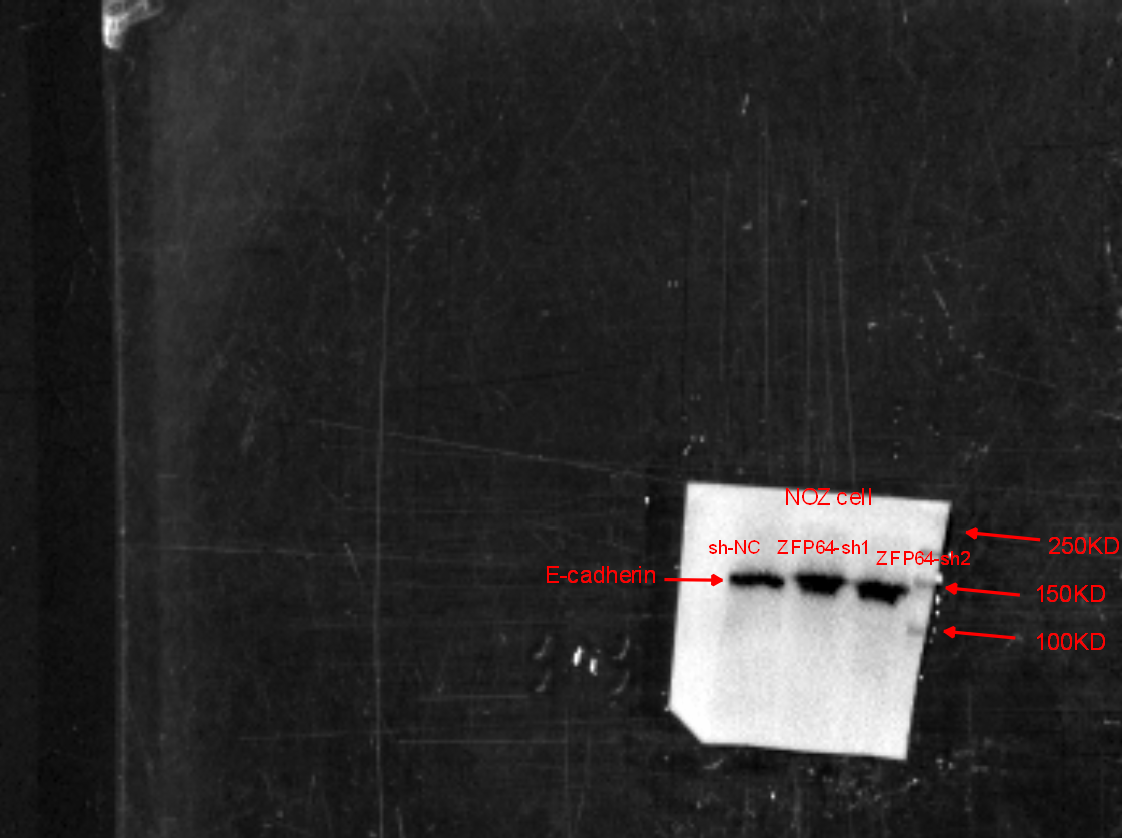

Supplement: Supplementary file 1 [file cancers-15-04508-s001.zip › cancers-2573702-supplementary/Figure S3-Figure 2E/E-cadherin (NOZ).tif]

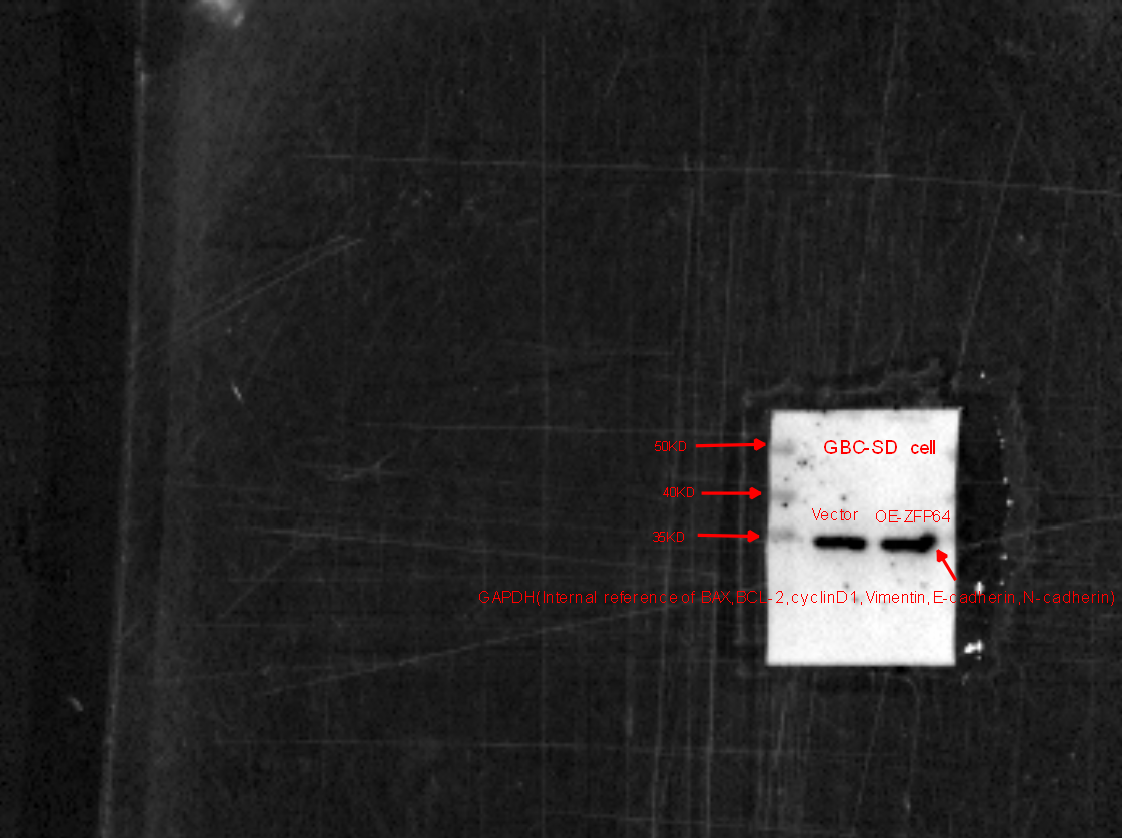

Supplement: Supplementary file 1 [file cancers-15-04508-s001.zip › cancers-2573702-supplementary/Figure S3-Figure 2E/GAPDH (GBC-SD).tif]

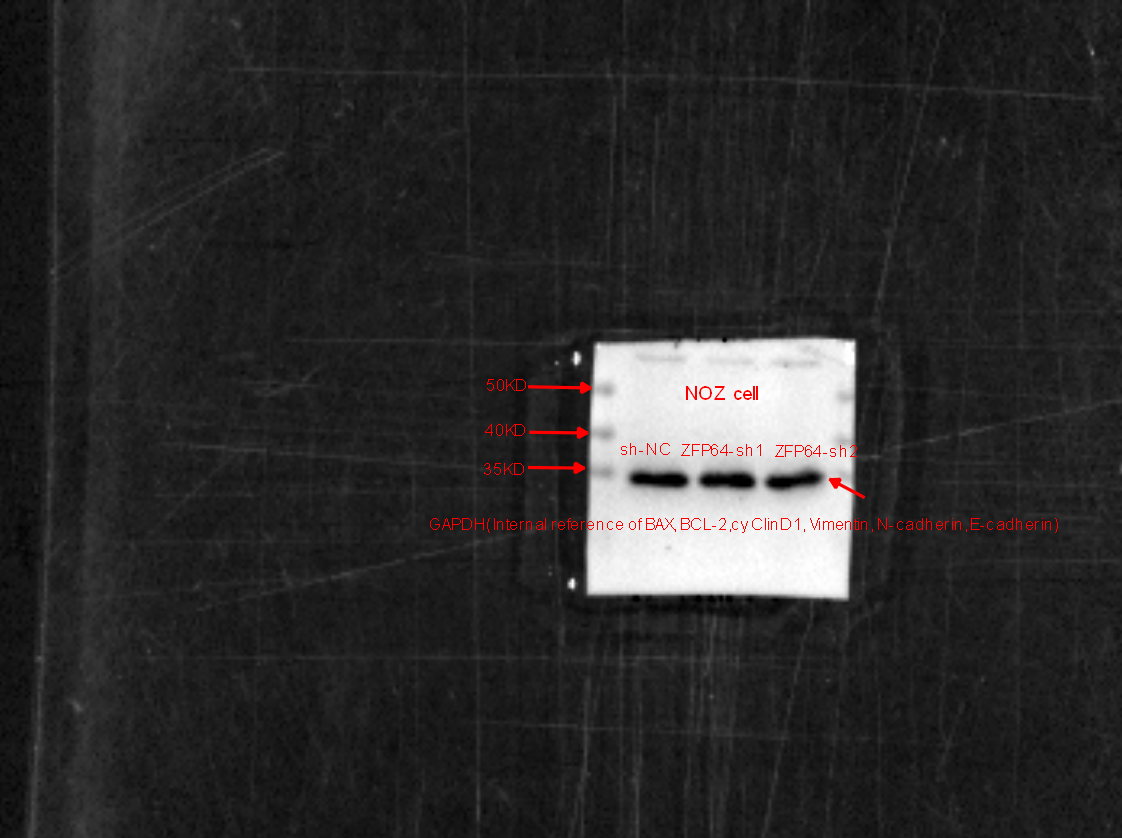

Supplement: Supplementary file 1 [file cancers-15-04508-s001.zip › cancers-2573702-supplementary/Figure S3-Figure 2E/GAPDH(NOZ).tif]

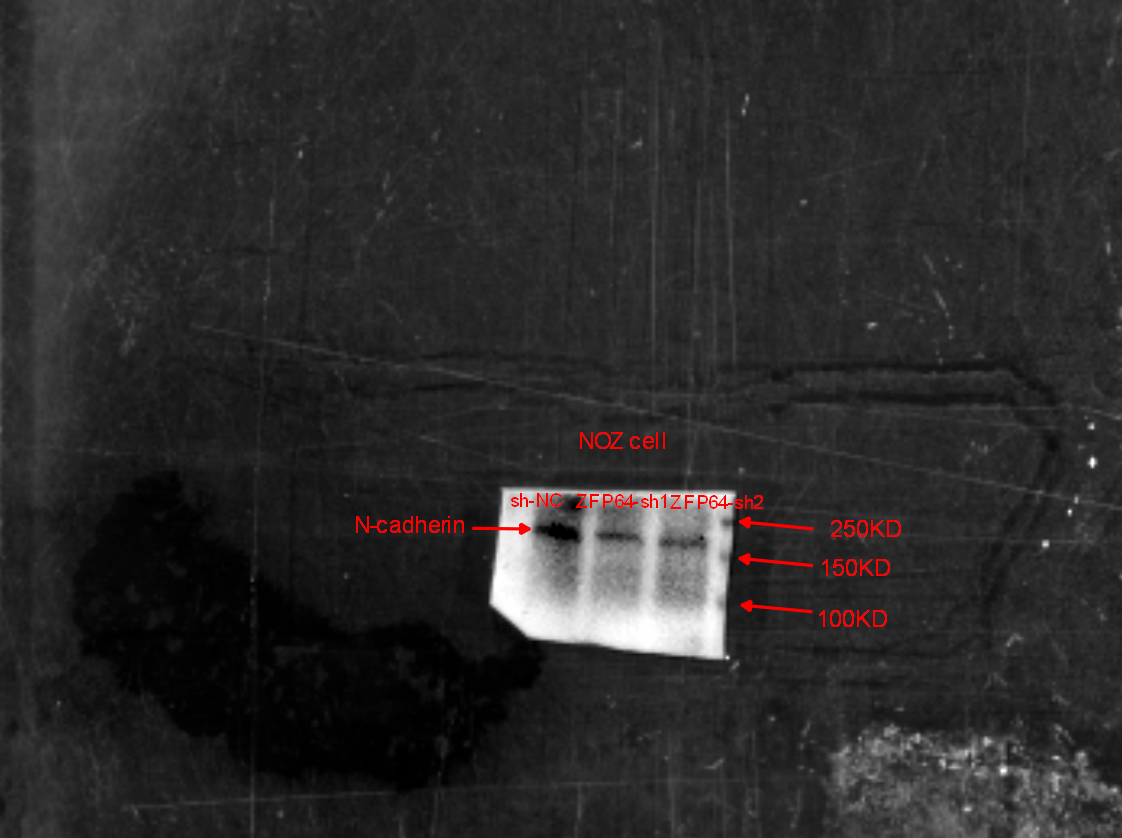

Supplement: Supplementary file 1 [file cancers-15-04508-s001.zip › cancers-2573702-supplementary/Figure S3-Figure 2E/N-cadhein (NOZ).tif]

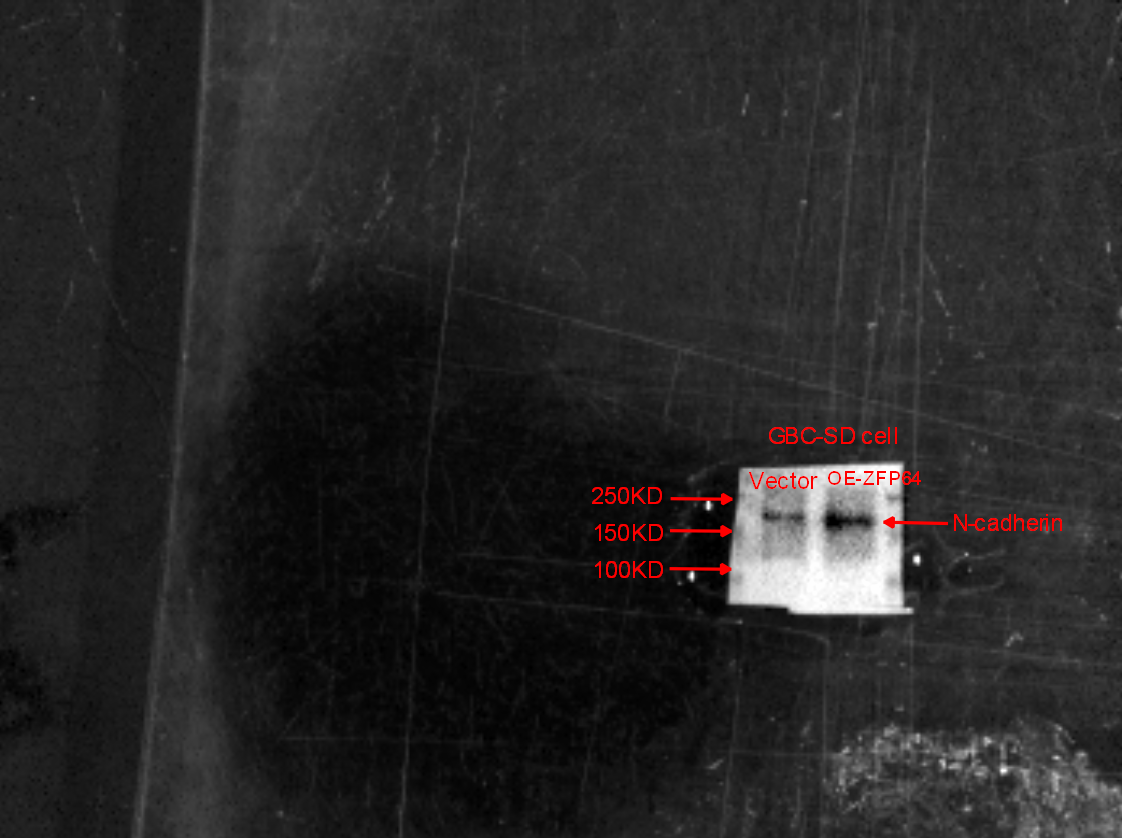

Supplement: Supplementary file 1 [file cancers-15-04508-s001.zip › cancers-2573702-supplementary/Figure S3-Figure 2E/N-cadherin (GBC-SD).tif]

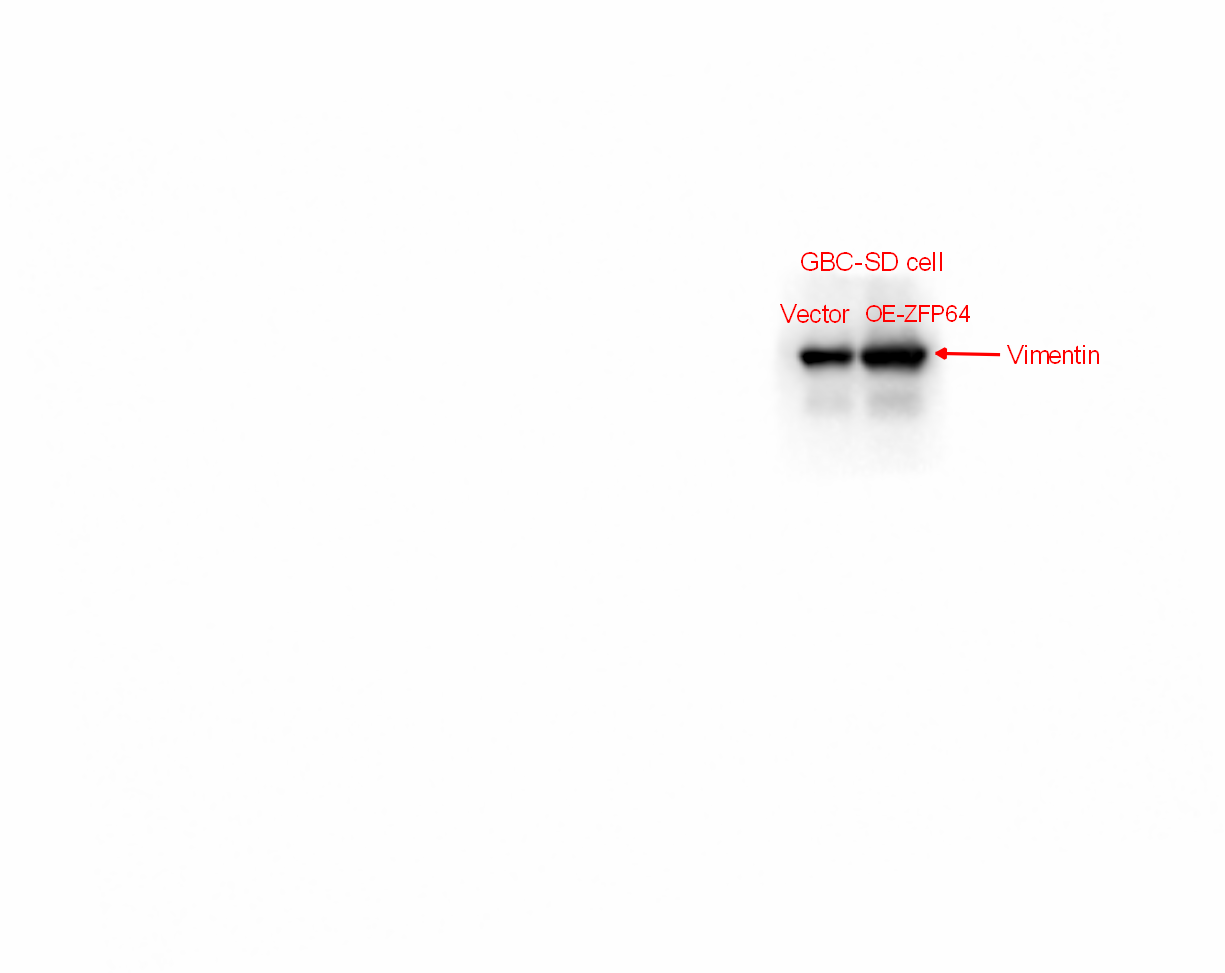

Supplement: Supplementary file 1 [file cancers-15-04508-s001.zip › cancers-2573702-supplementary/Figure S3-Figure 2E/Vimentin (GBC-SD).tif]

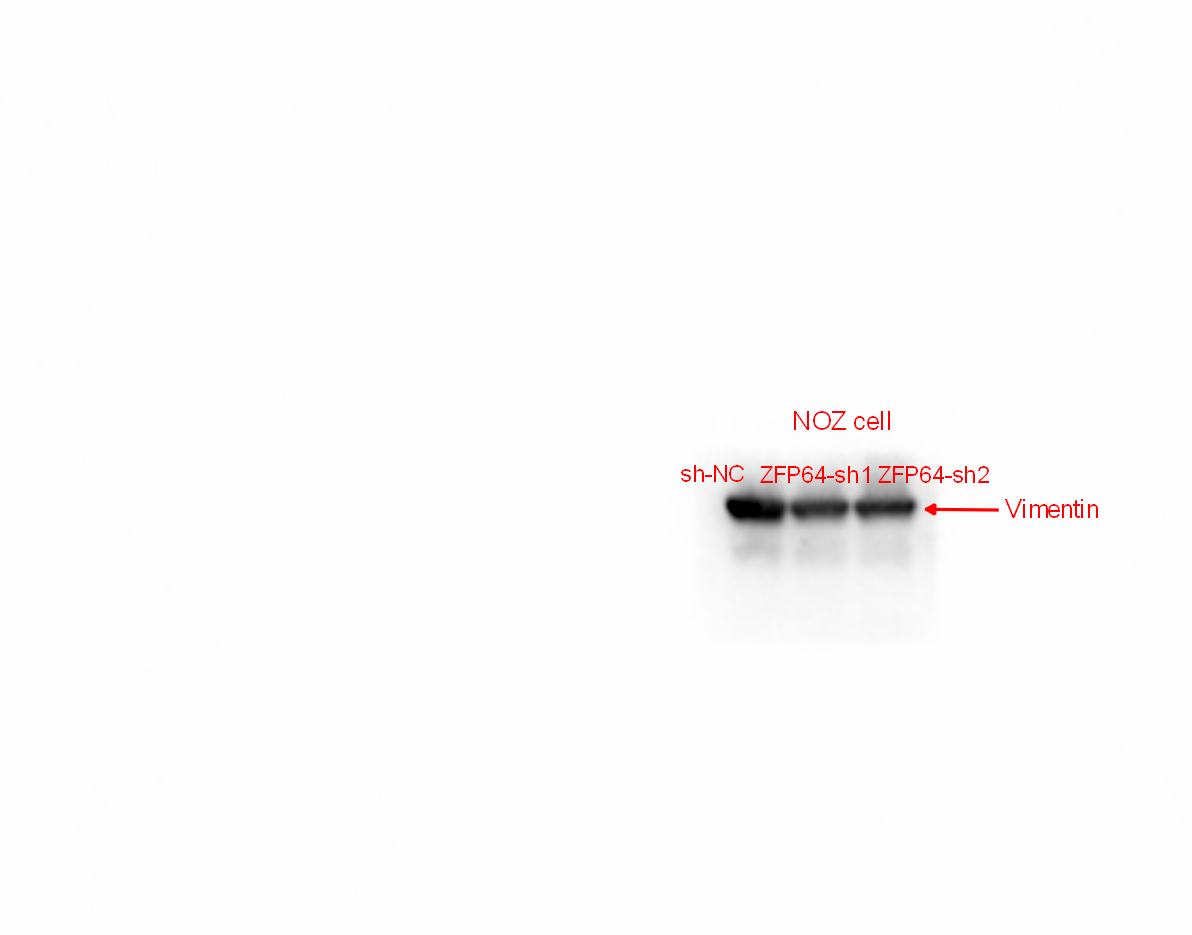

Supplement: Supplementary file 1 [file cancers-15-04508-s001.zip › cancers-2573702-supplementary/Figure S3-Figure 2E/Vimentin (NOZ).tif]

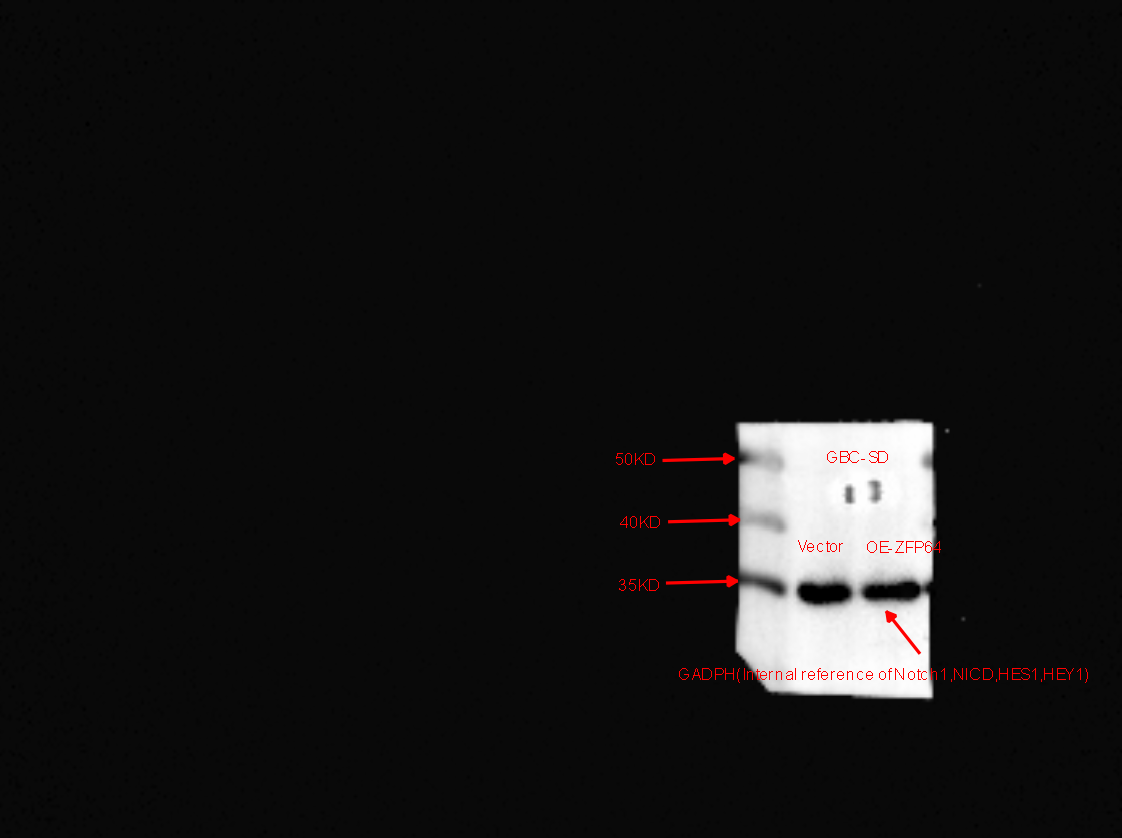

Supplement: Supplementary file 1 [file cancers-15-04508-s001.zip › cancers-2573702-supplementary/Figure S4-Figure 3A/GAPDH (GBC-SD).tif]

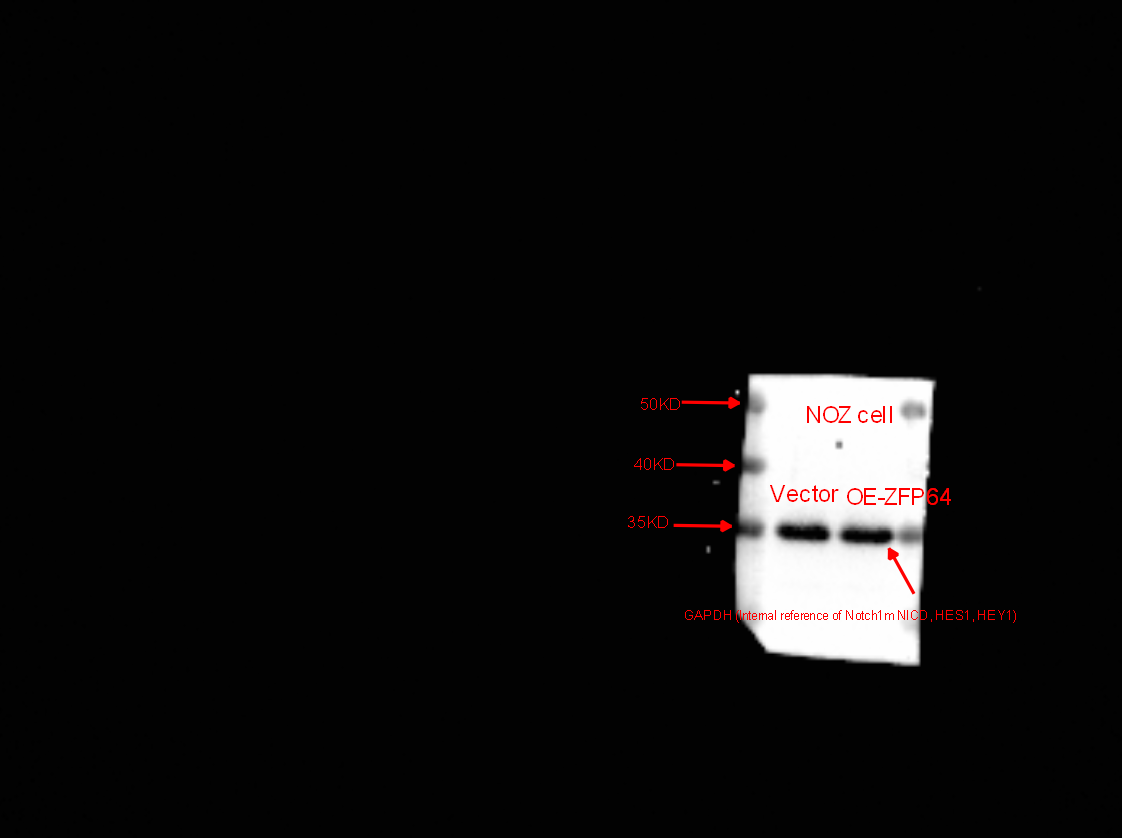

Supplement: Supplementary file 1 [file cancers-15-04508-s001.zip › cancers-2573702-supplementary/Figure S4-Figure 3A/GAPDH (NOZ).tif]

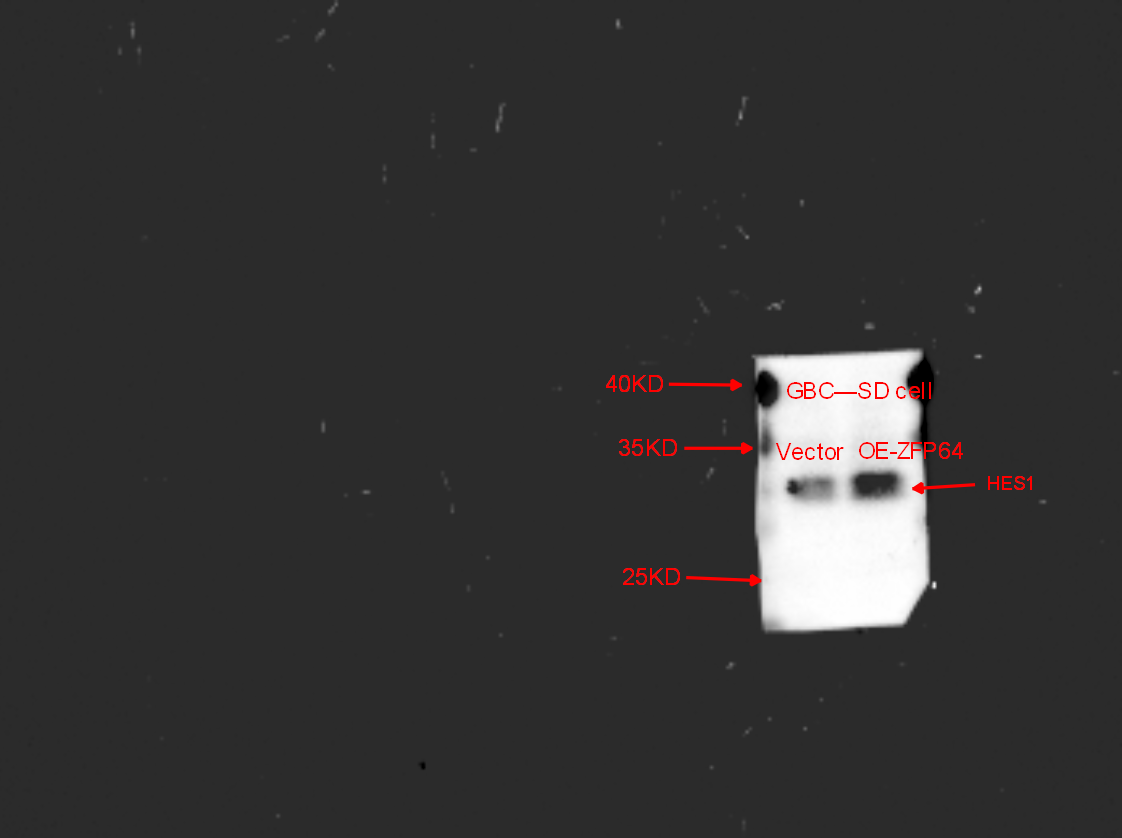

Supplement: Supplementary file 1 [file cancers-15-04508-s001.zip › cancers-2573702-supplementary/Figure S4-Figure 3A/HES1 (GBC-SD).tif]

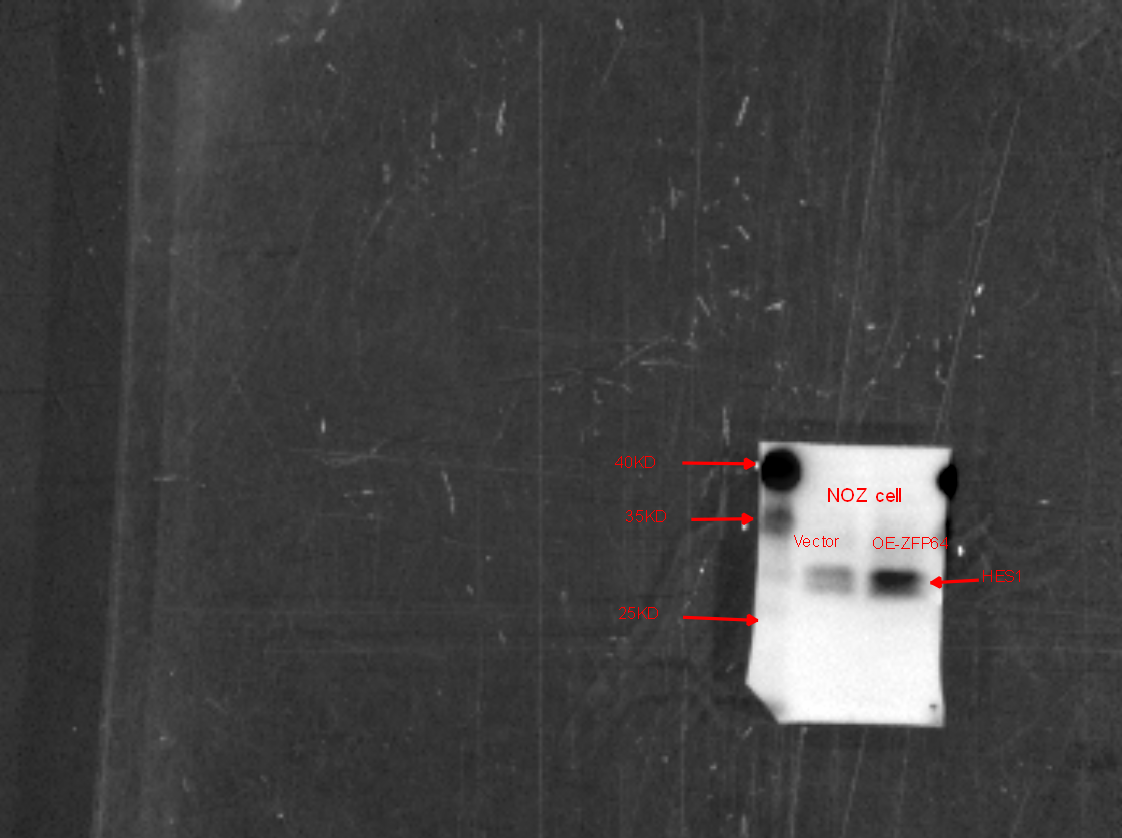

Supplement: Supplementary file 1 [file cancers-15-04508-s001.zip › cancers-2573702-supplementary/Figure S4-Figure 3A/HES1 (NOZ).tif]

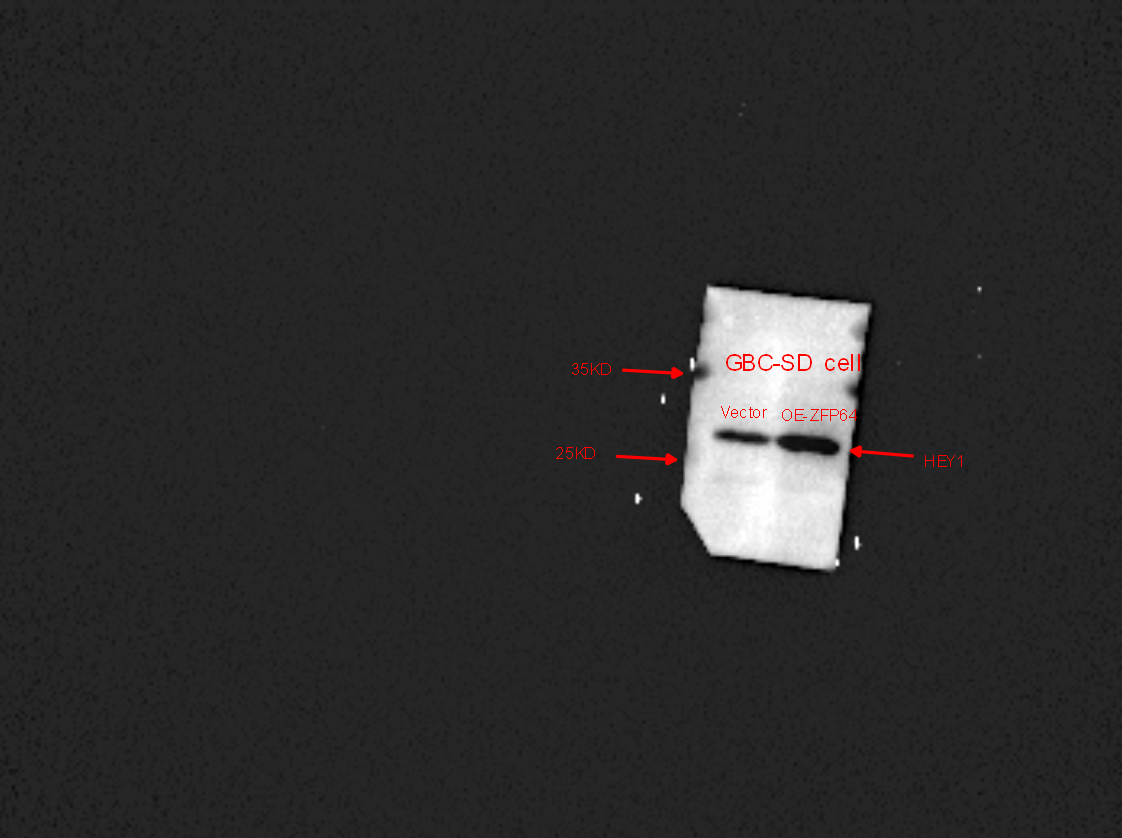

Supplement: Supplementary file 1 [file cancers-15-04508-s001.zip › cancers-2573702-supplementary/Figure S4-Figure 3A/HEY1 (GBC-SD).tif]

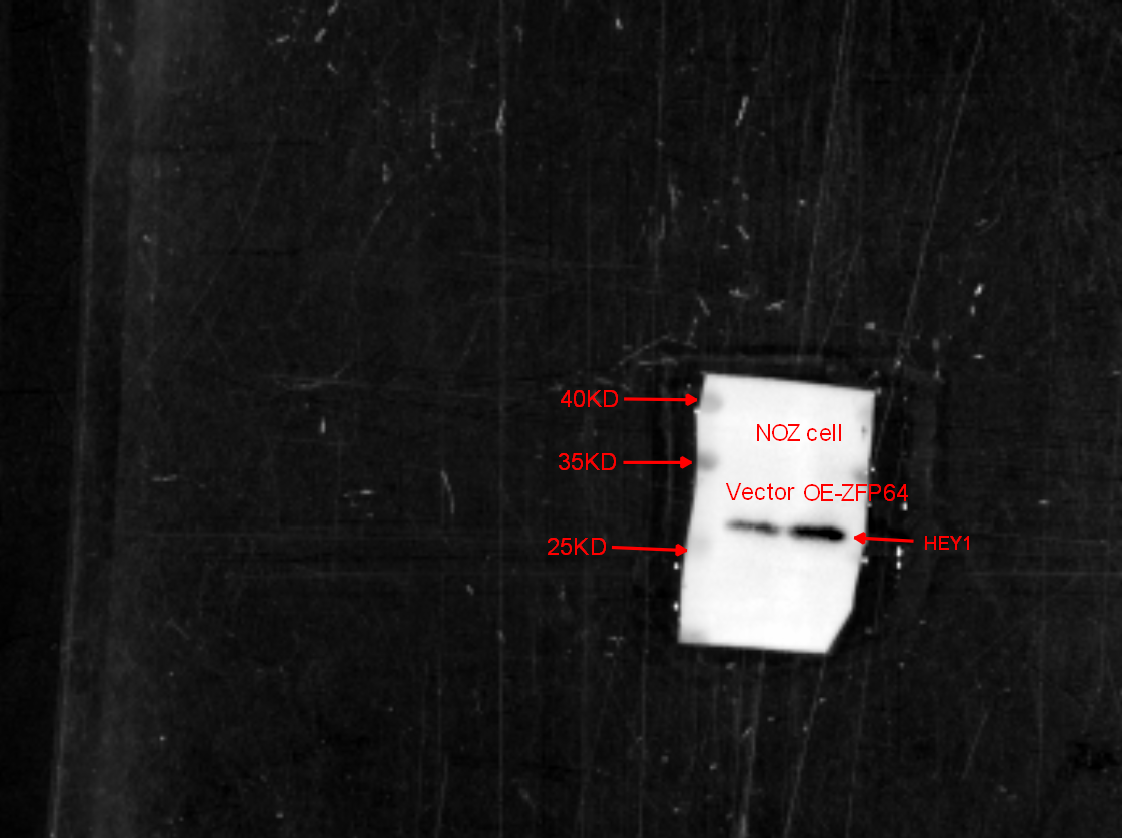

Supplement: Supplementary file 1 [file cancers-15-04508-s001.zip › cancers-2573702-supplementary/Figure S4-Figure 3A/HEY1 (NOZ).tif]

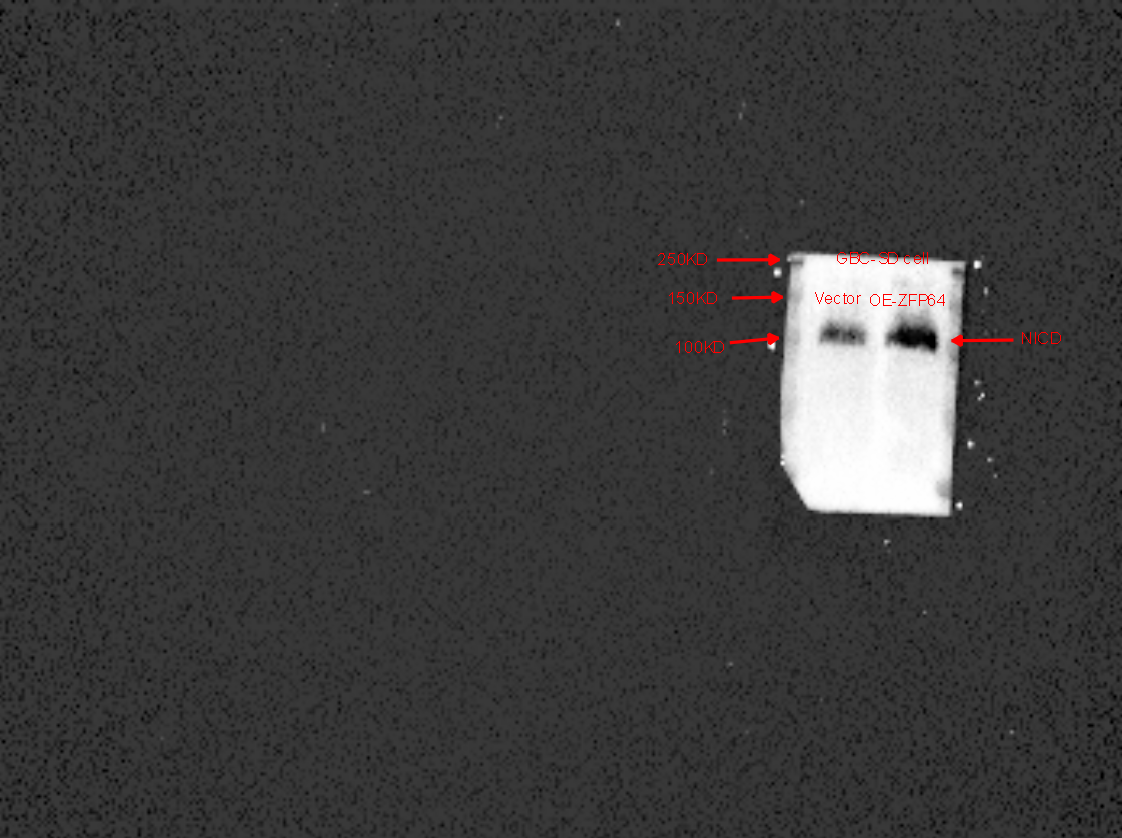

Supplement: Supplementary file 1 [file cancers-15-04508-s001.zip › cancers-2573702-supplementary/Figure S4-Figure 3A/NICD (GBC-SD).tif]

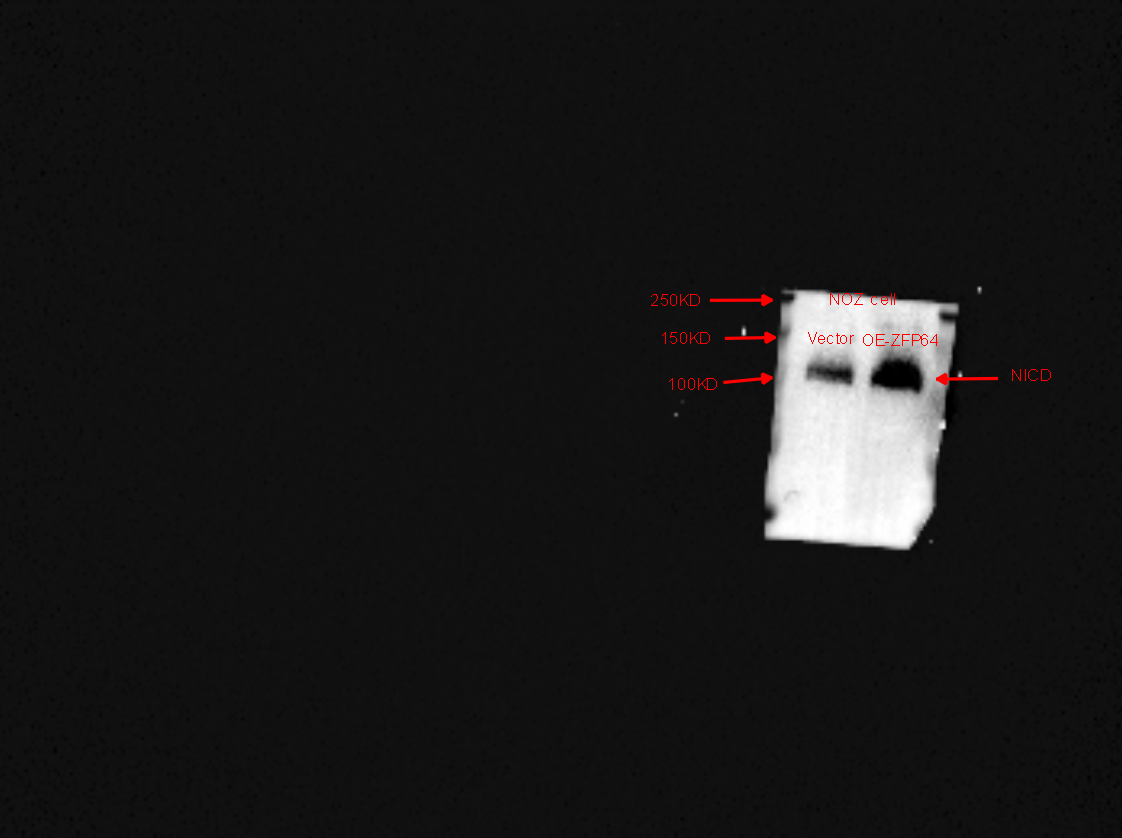

Supplement: Supplementary file 1 [file cancers-15-04508-s001.zip › cancers-2573702-supplementary/Figure S4-Figure 3A/NICD (NOZ).tif]

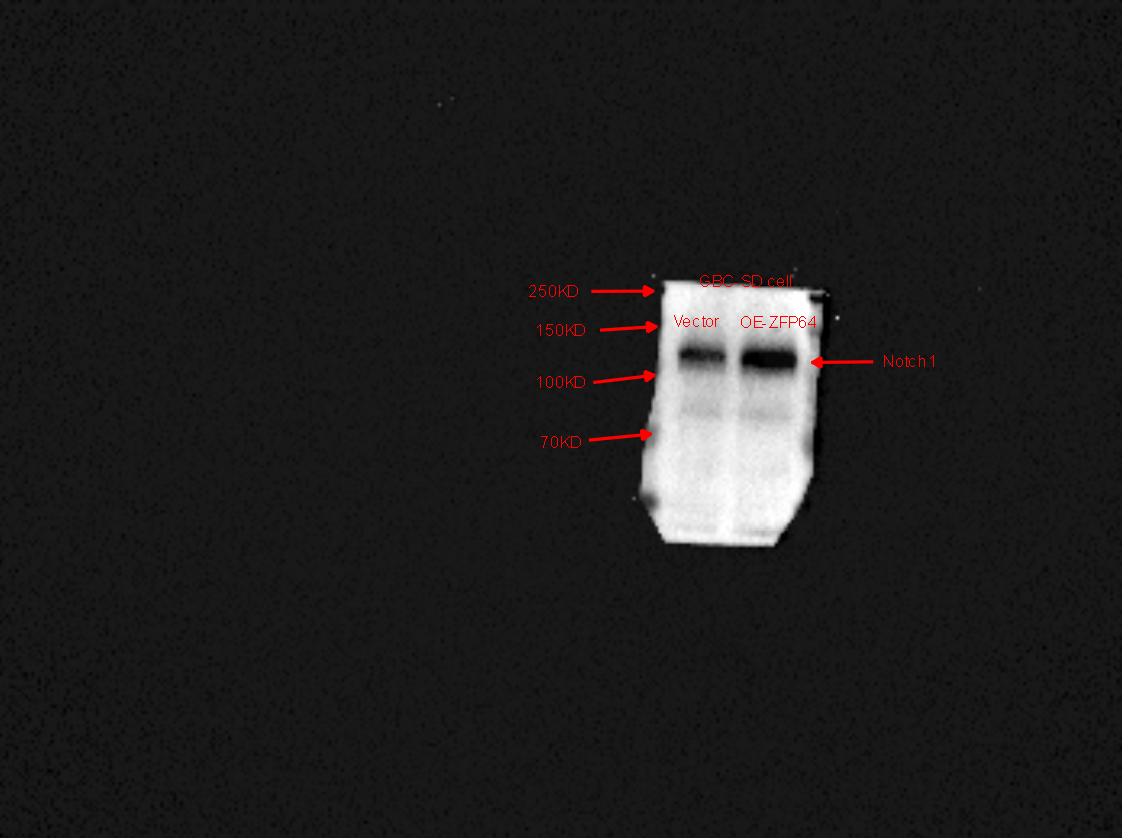

Supplement: Supplementary file 1 [file cancers-15-04508-s001.zip › cancers-2573702-supplementary/Figure S4-Figure 3A/Notch1 (GBC-SD).tif]

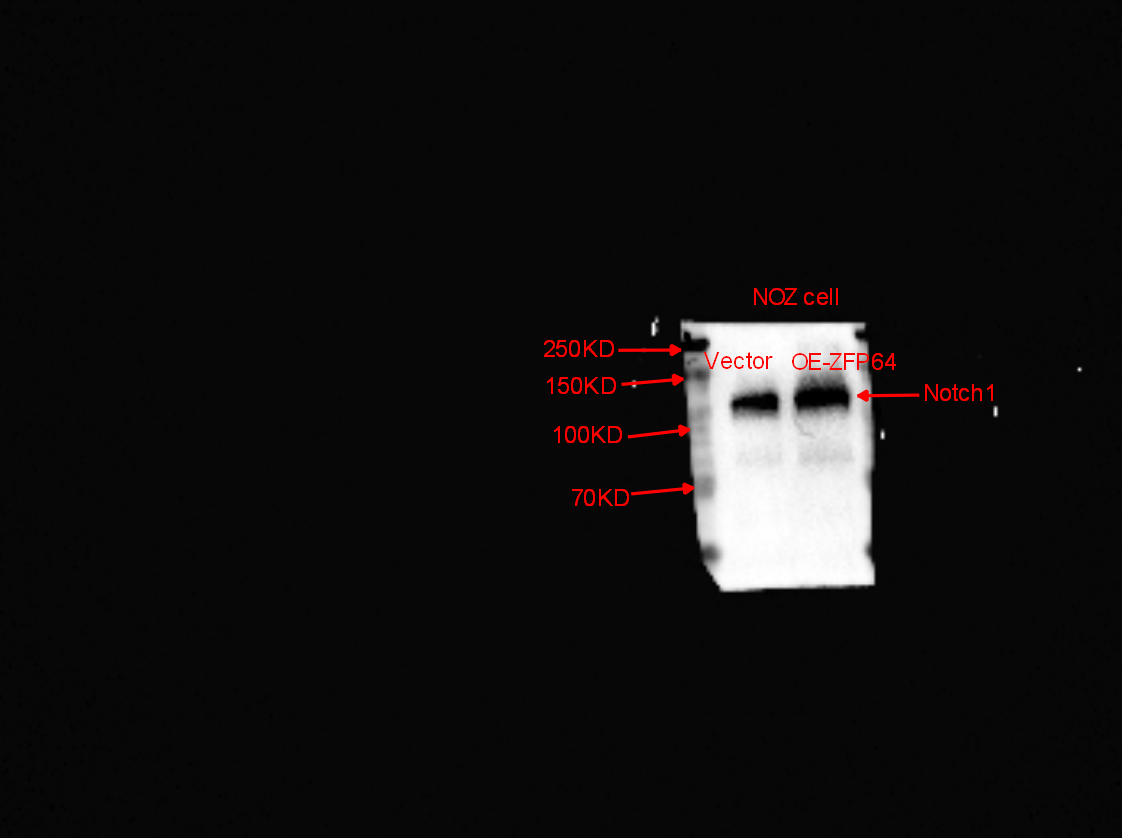

Supplement: Supplementary file 1 [file cancers-15-04508-s001.zip › cancers-2573702-supplementary/Figure S4-Figure 3A/Notch1 (NOZ ).tif]

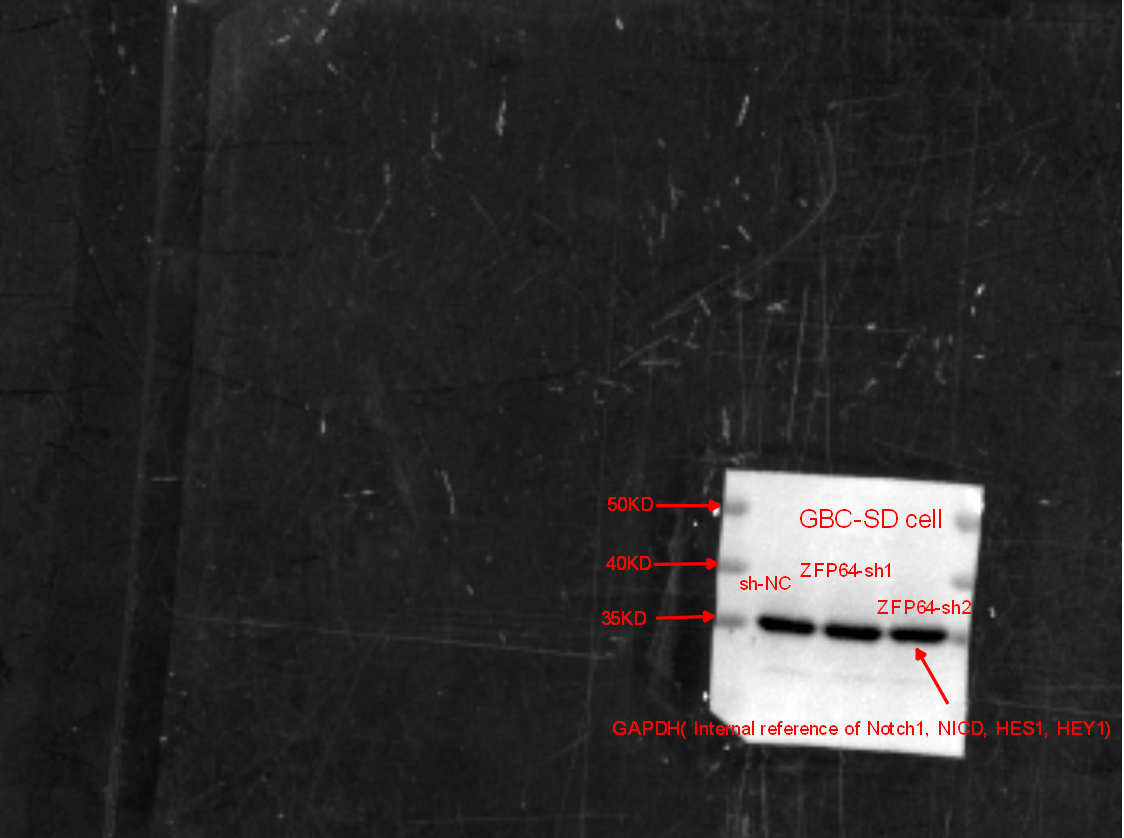

Supplement: Supplementary file 1 [file cancers-15-04508-s001.zip › cancers-2573702-supplementary/Figure S5-Figure 3B/GAPDH (GBC-SD).tif]

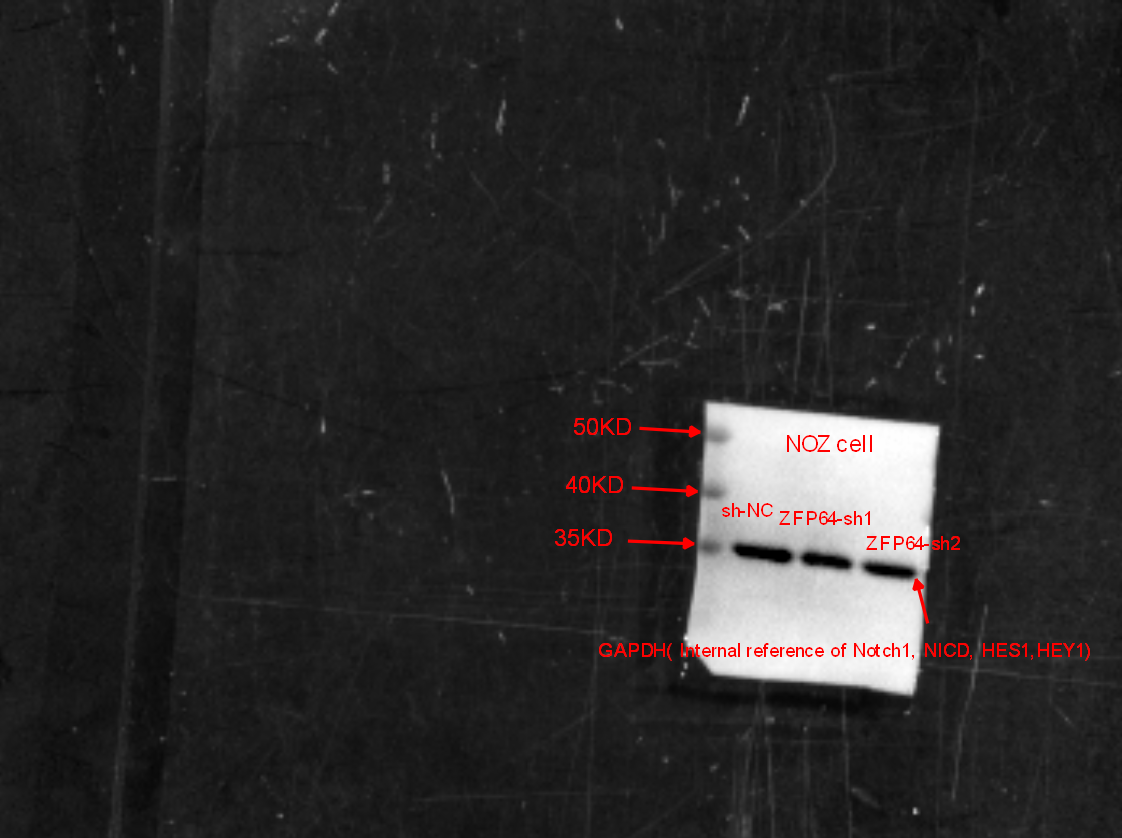

Supplement: Supplementary file 1 [file cancers-15-04508-s001.zip › cancers-2573702-supplementary/Figure S5-Figure 3B/GAPDH (NOZ).tif]

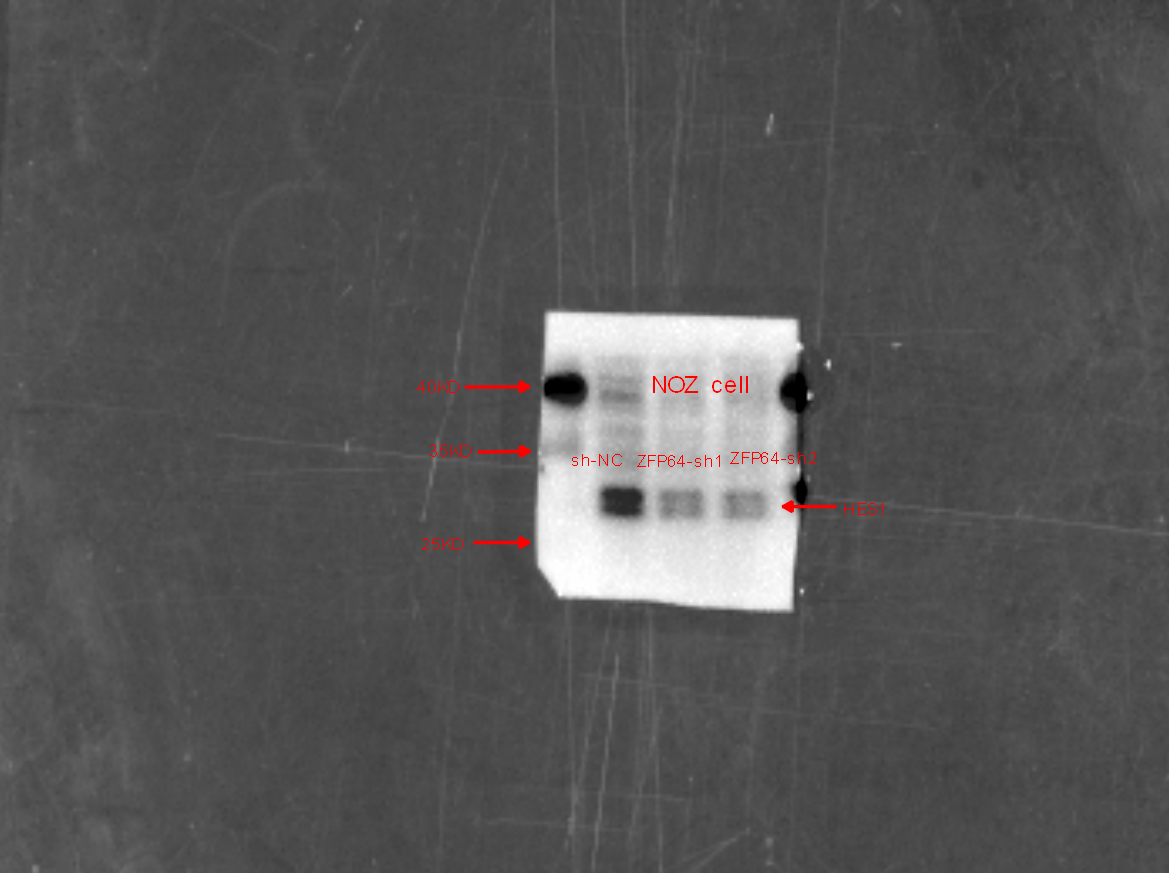

Supplement: Supplementary file 1 [file cancers-15-04508-s001.zip › cancers-2573702-supplementary/Figure S5-Figure 3B/HES1 (NOZ).tif]

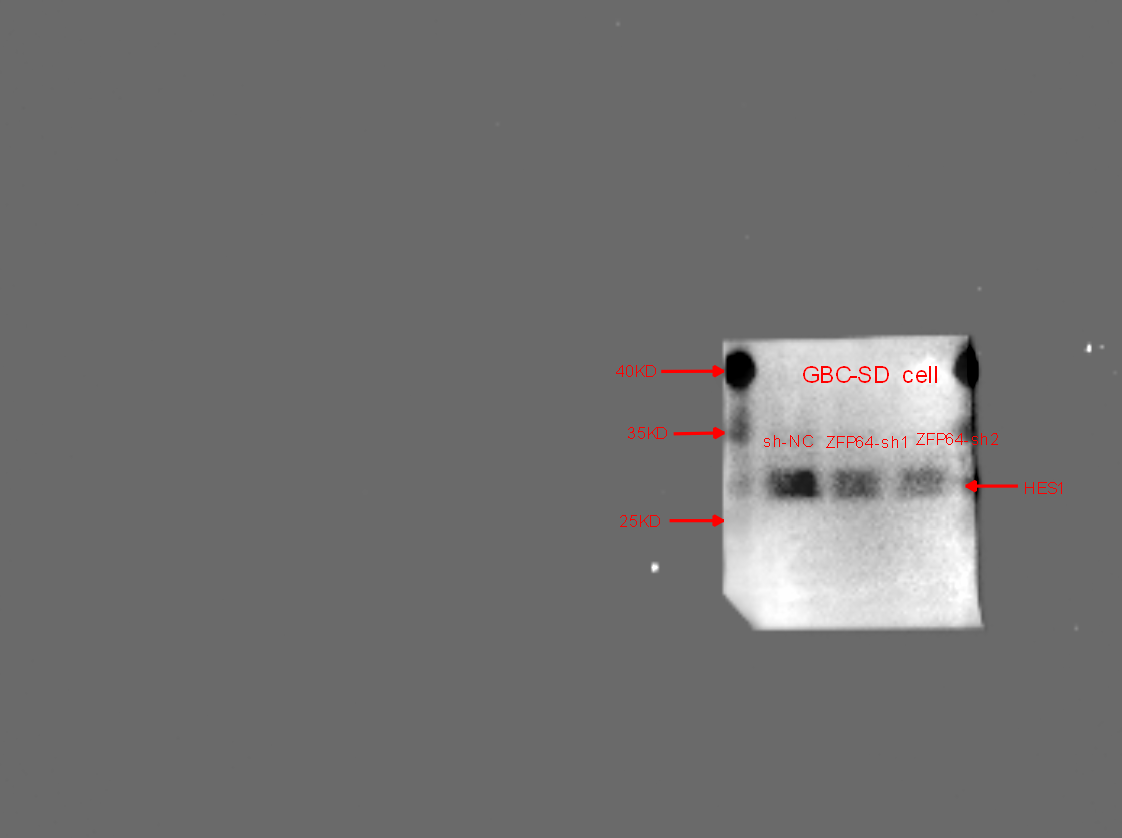

Supplement: Supplementary file 1 [file cancers-15-04508-s001.zip › cancers-2573702-supplementary/Figure S5-Figure 3B/HES1( GBC-SD).tif]

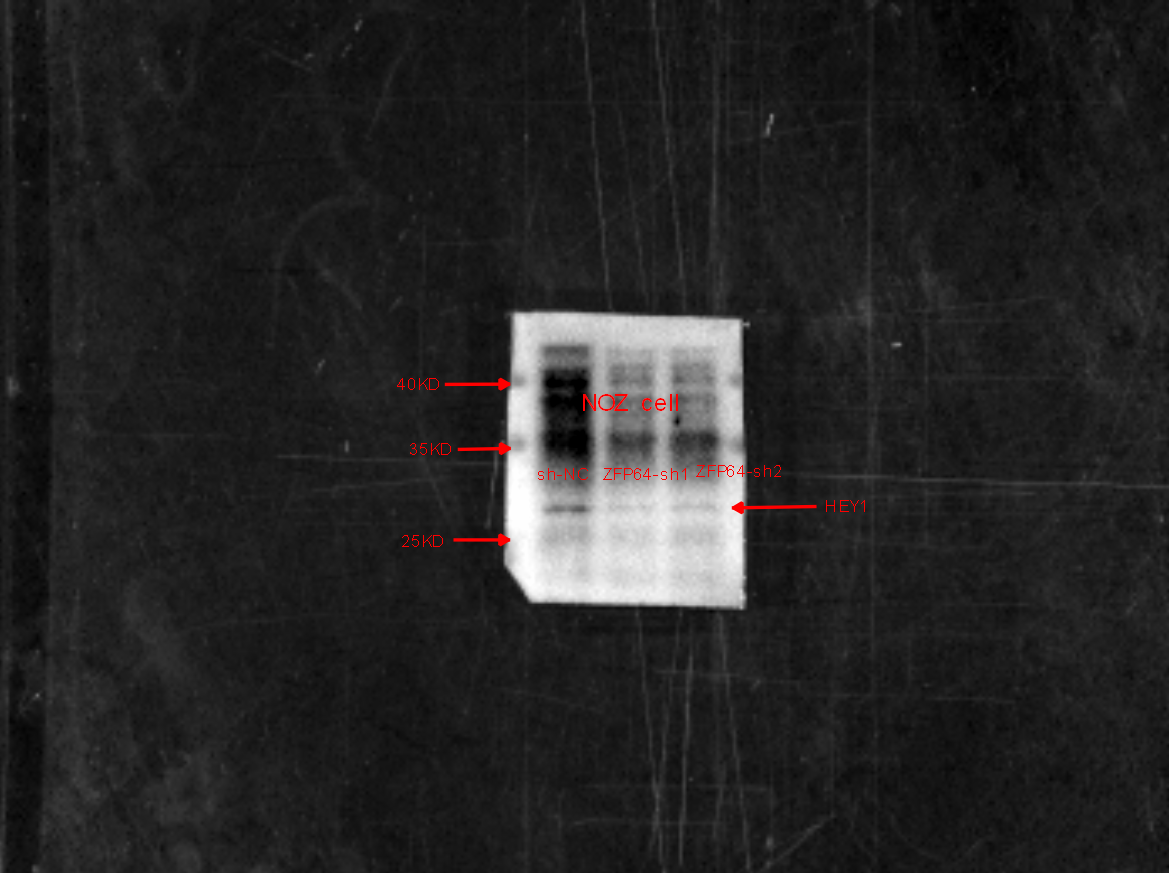

Supplement: Supplementary file 1 [file cancers-15-04508-s001.zip › cancers-2573702-supplementary/Figure S5-Figure 3B/HEY (NOZ).tif]

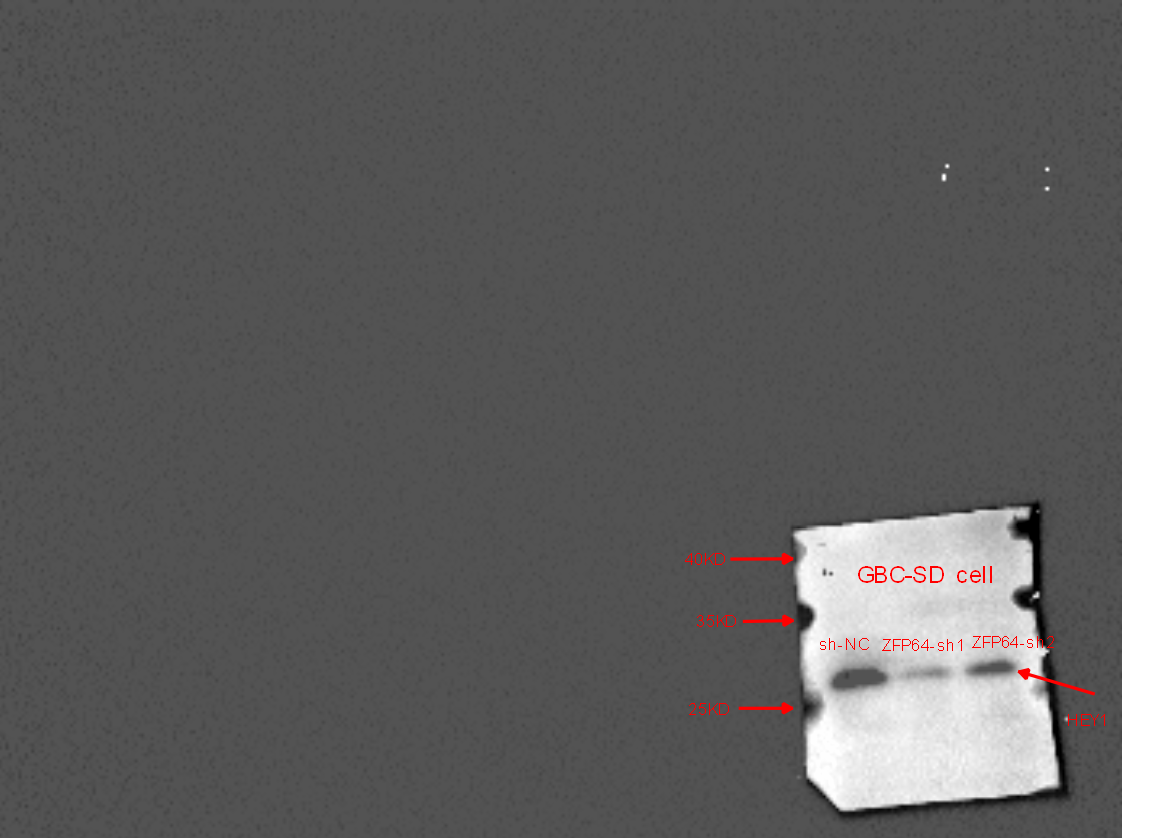

Supplement: Supplementary file 1 [file cancers-15-04508-s001.zip › cancers-2573702-supplementary/Figure S5-Figure 3B/HEY1( GBC-SD).tif]

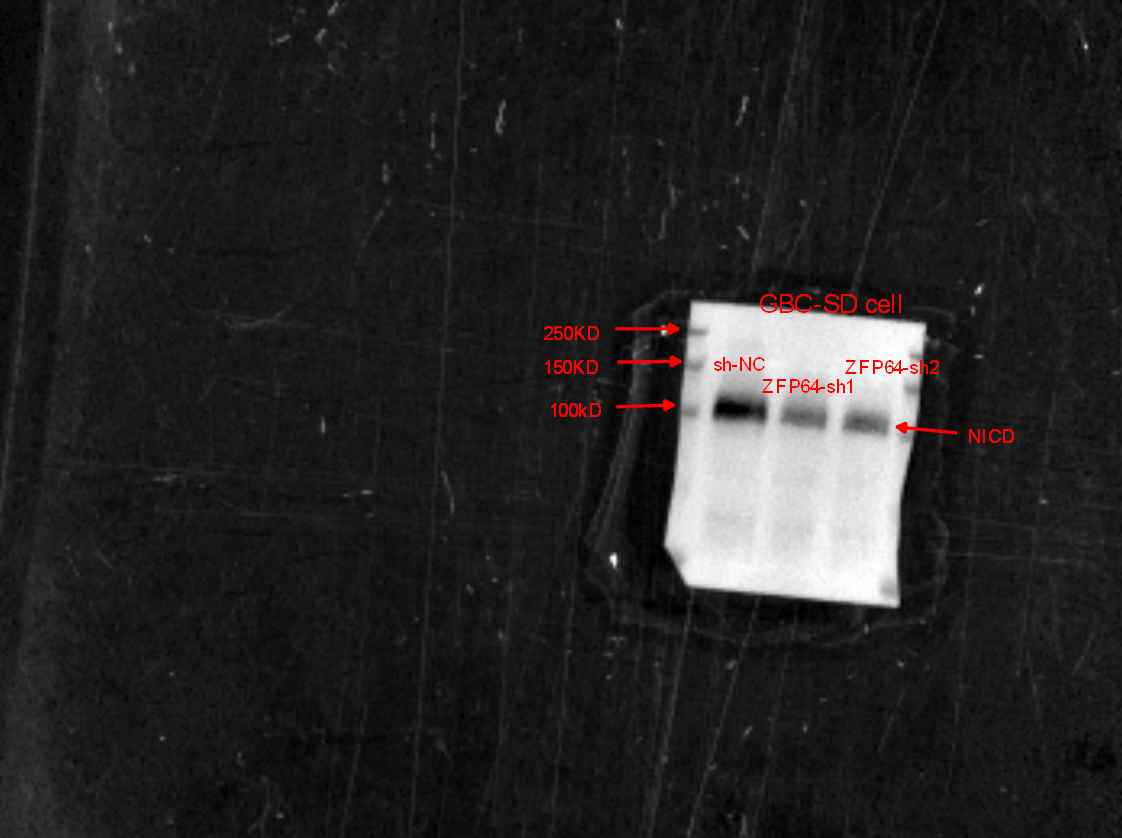

Supplement: Supplementary file 1 [file cancers-15-04508-s001.zip › cancers-2573702-supplementary/Figure S5-Figure 3B/NICD (GBC-SD).tif]

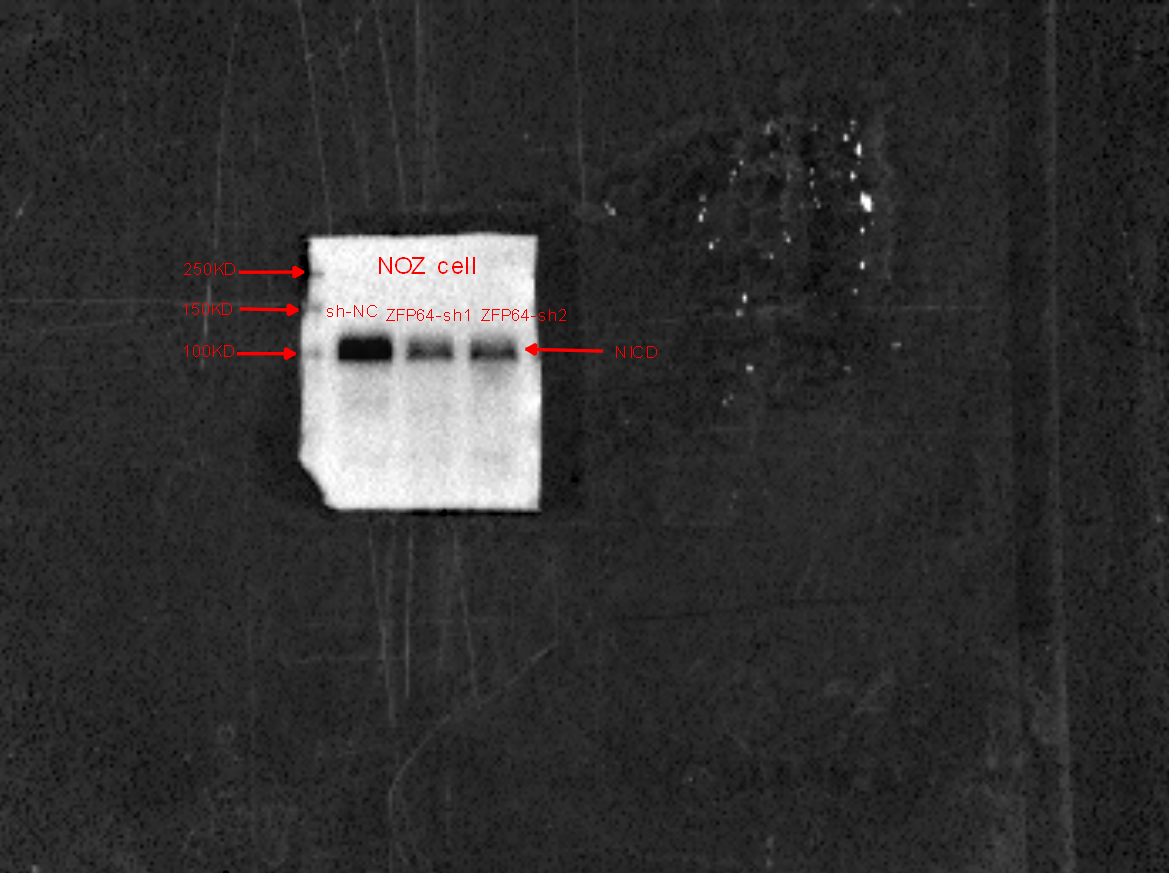

Supplement: Supplementary file 1 [file cancers-15-04508-s001.zip › cancers-2573702-supplementary/Figure S5-Figure 3B/NICD (NOZ).tif]

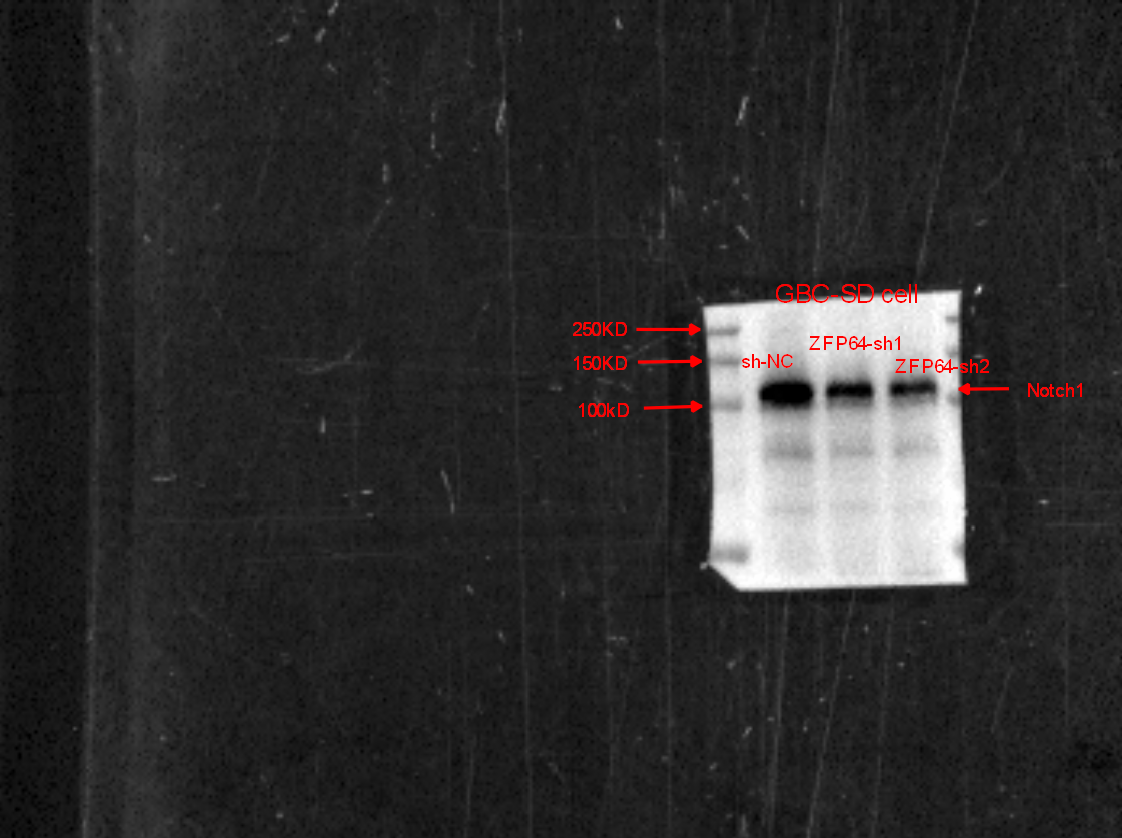

Supplement: Supplementary file 1 [file cancers-15-04508-s001.zip › cancers-2573702-supplementary/Figure S5-Figure 3B/Notch1 (GBC-SD).tif]

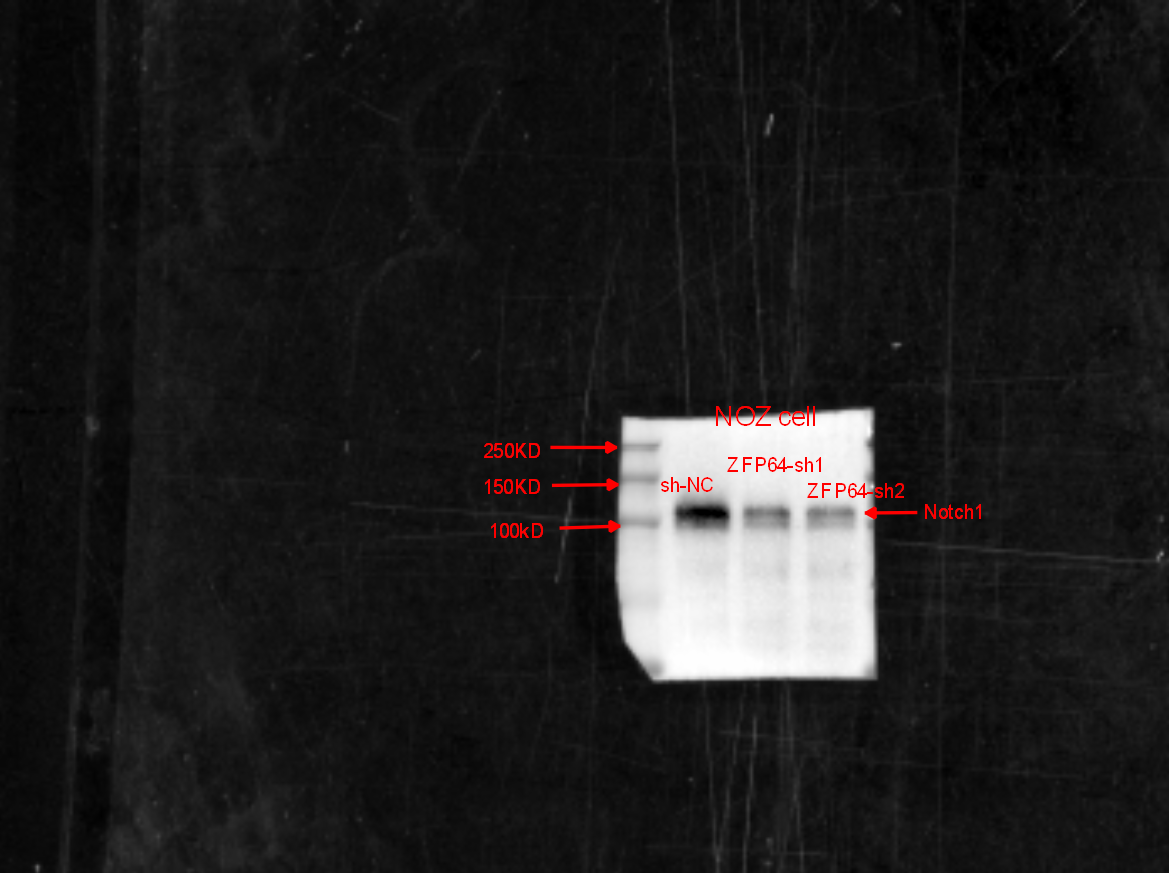

Supplement: Supplementary file 1 [file cancers-15-04508-s001.zip › cancers-2573702-supplementary/Figure S5-Figure 3B/Notch1 (NOZ).tif]

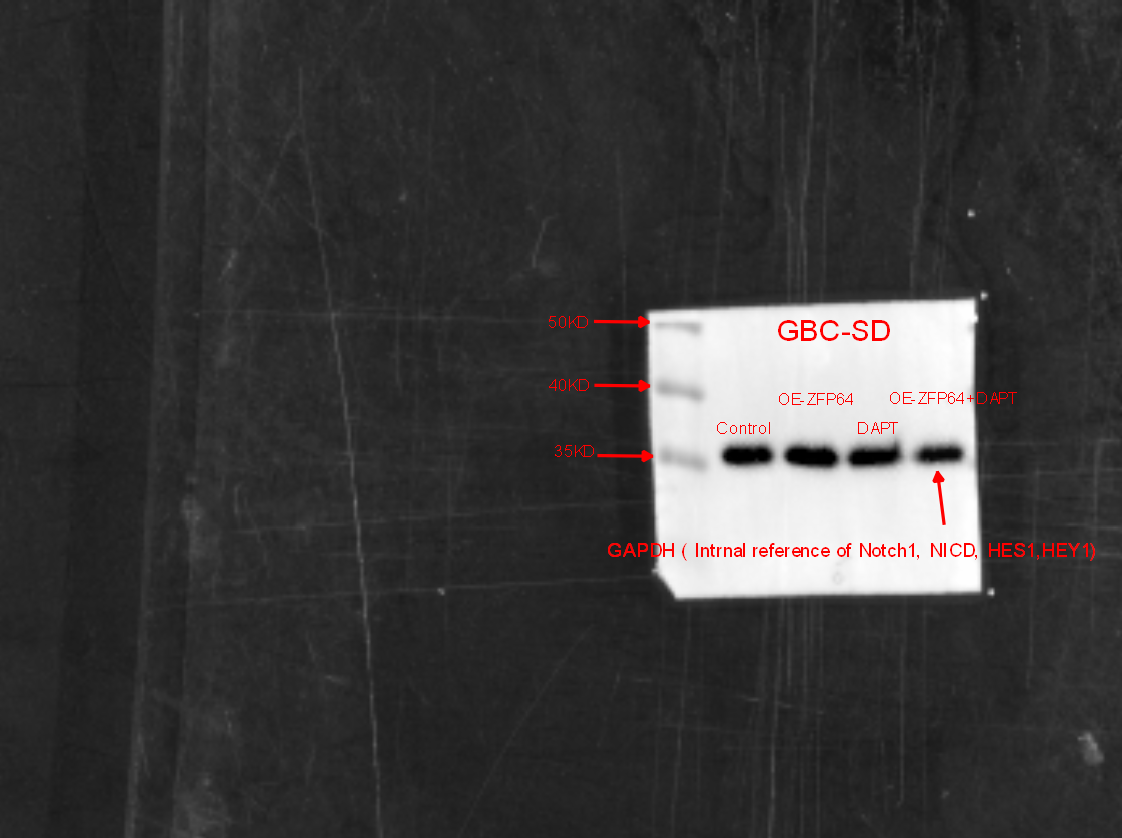

Supplement: Supplementary file 1 [file cancers-15-04508-s001.zip › cancers-2573702-supplementary/Figure S6-Figure 4A/GAPDH (GBC-SD).tif]

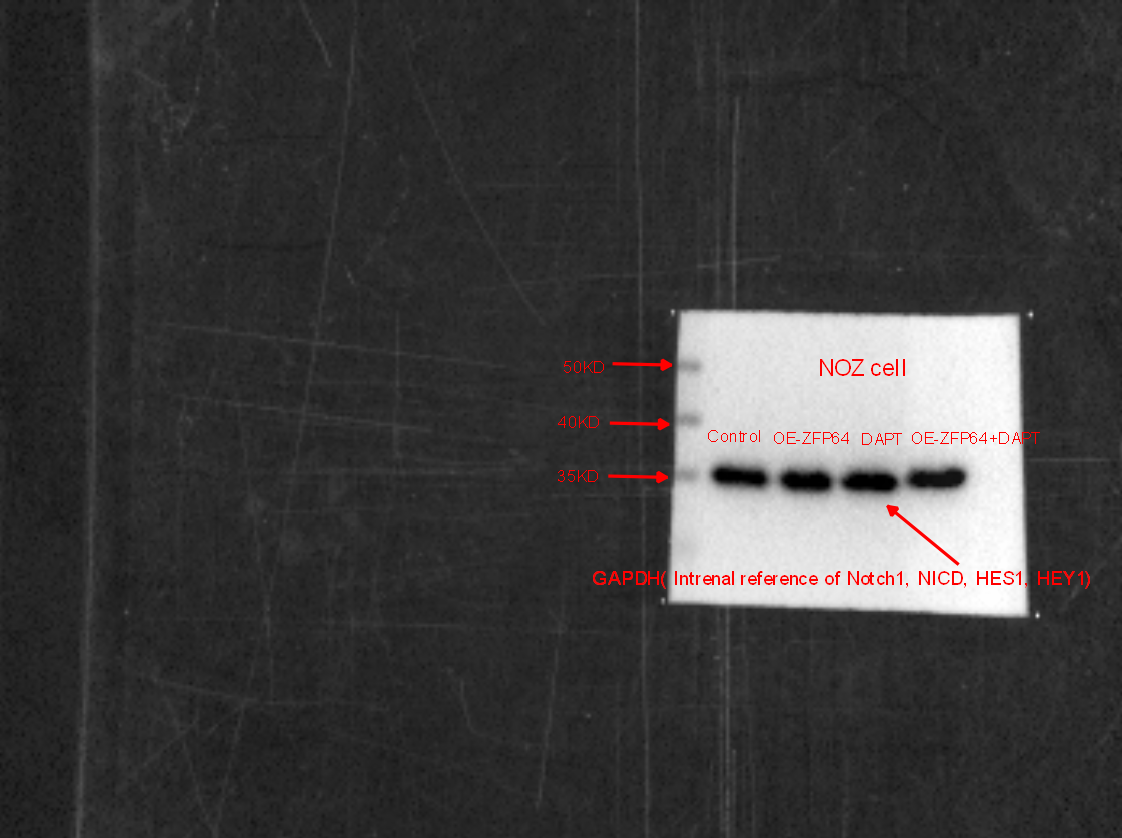

Supplement: Supplementary file 1 [file cancers-15-04508-s001.zip › cancers-2573702-supplementary/Figure S6-Figure 4A/GAPDH (NOZ).tif]

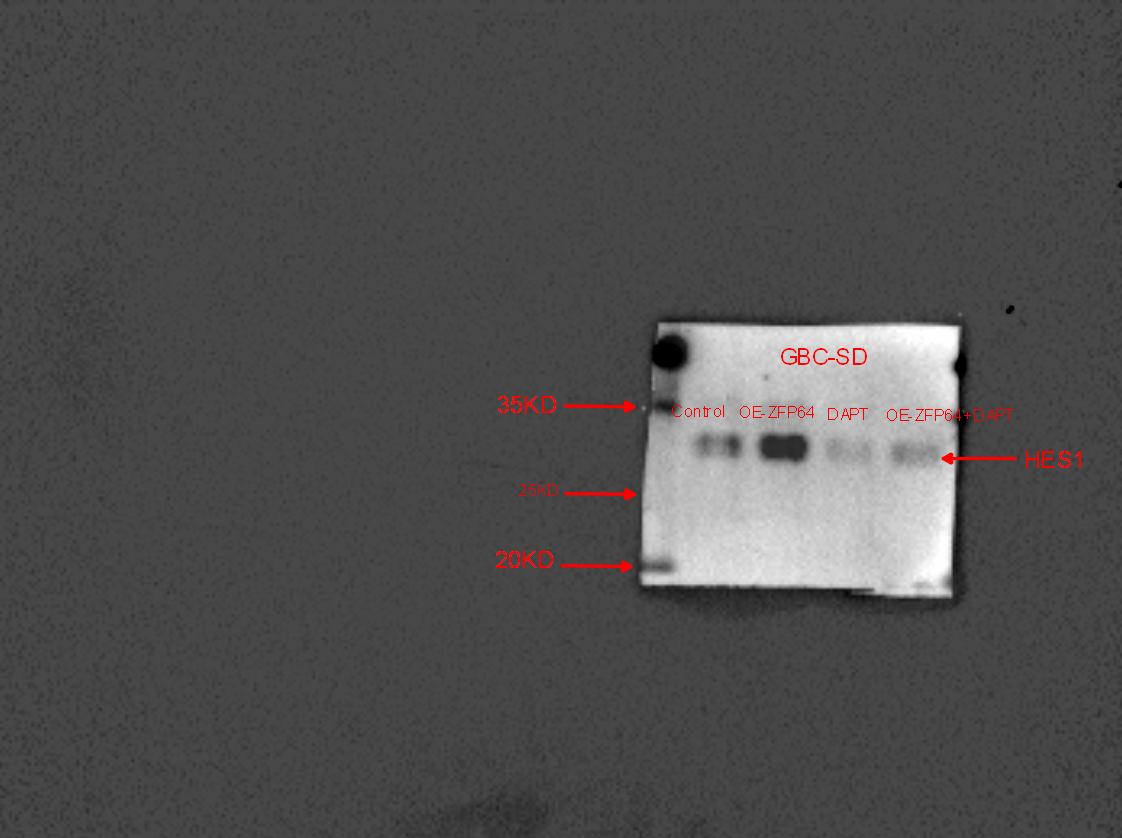

Supplement: Supplementary file 1 [file cancers-15-04508-s001.zip › cancers-2573702-supplementary/Figure S6-Figure 4A/HES1 (GBC-SD).tif]

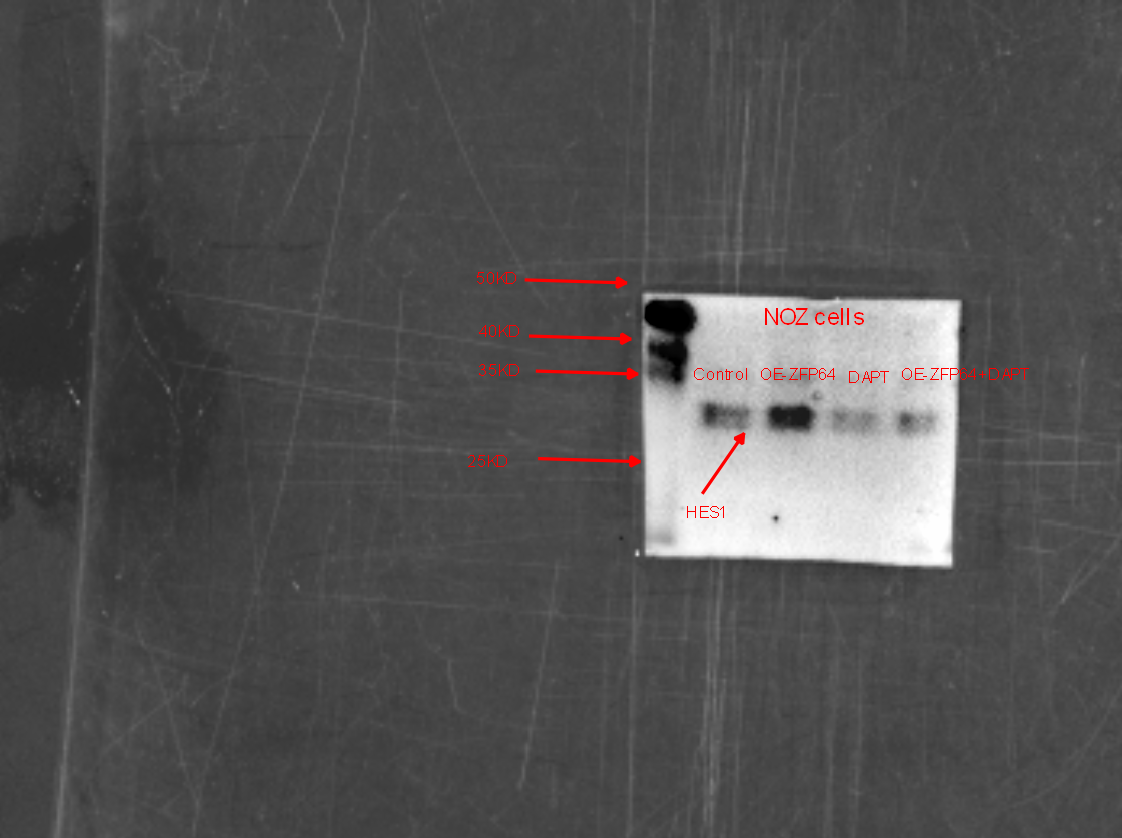

Supplement: Supplementary file 1 [file cancers-15-04508-s001.zip › cancers-2573702-supplementary/Figure S6-Figure 4A/HES1 (NOZ).tif]

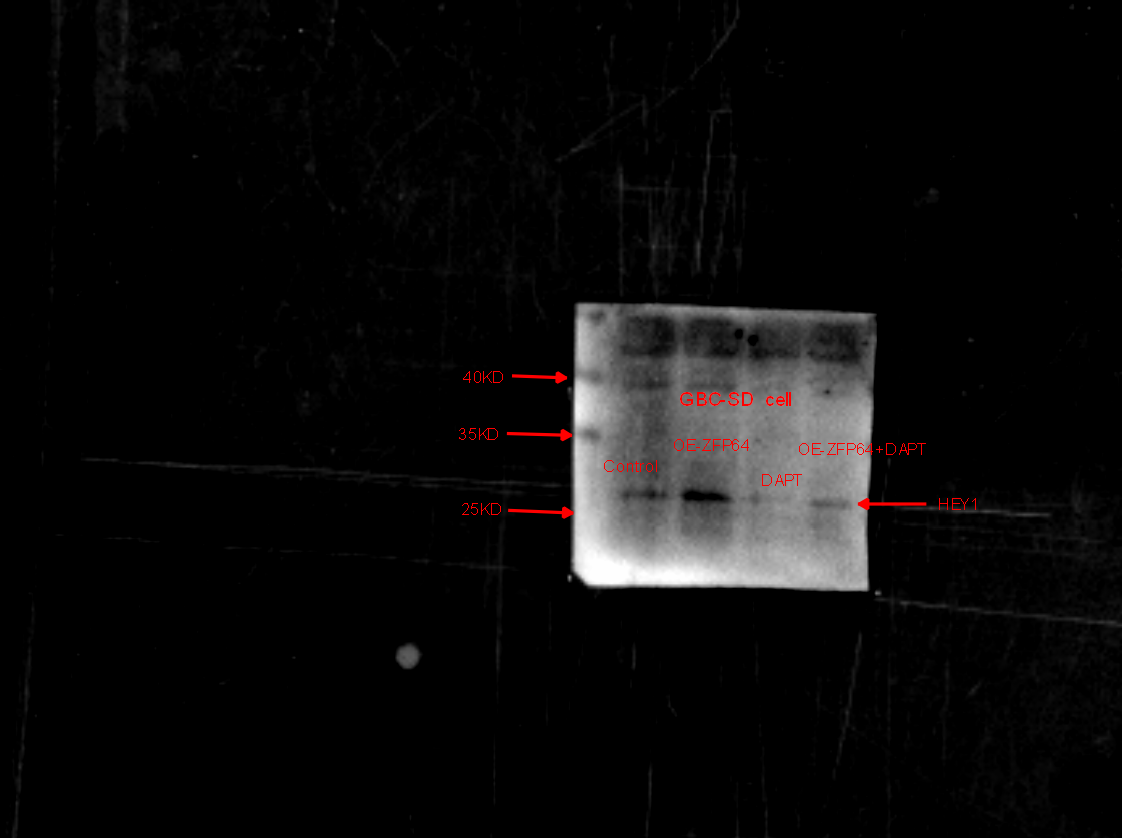

Supplement: Supplementary file 1 [file cancers-15-04508-s001.zip › cancers-2573702-supplementary/Figure S6-Figure 4A/HEY1 (GBC-SD).tif]

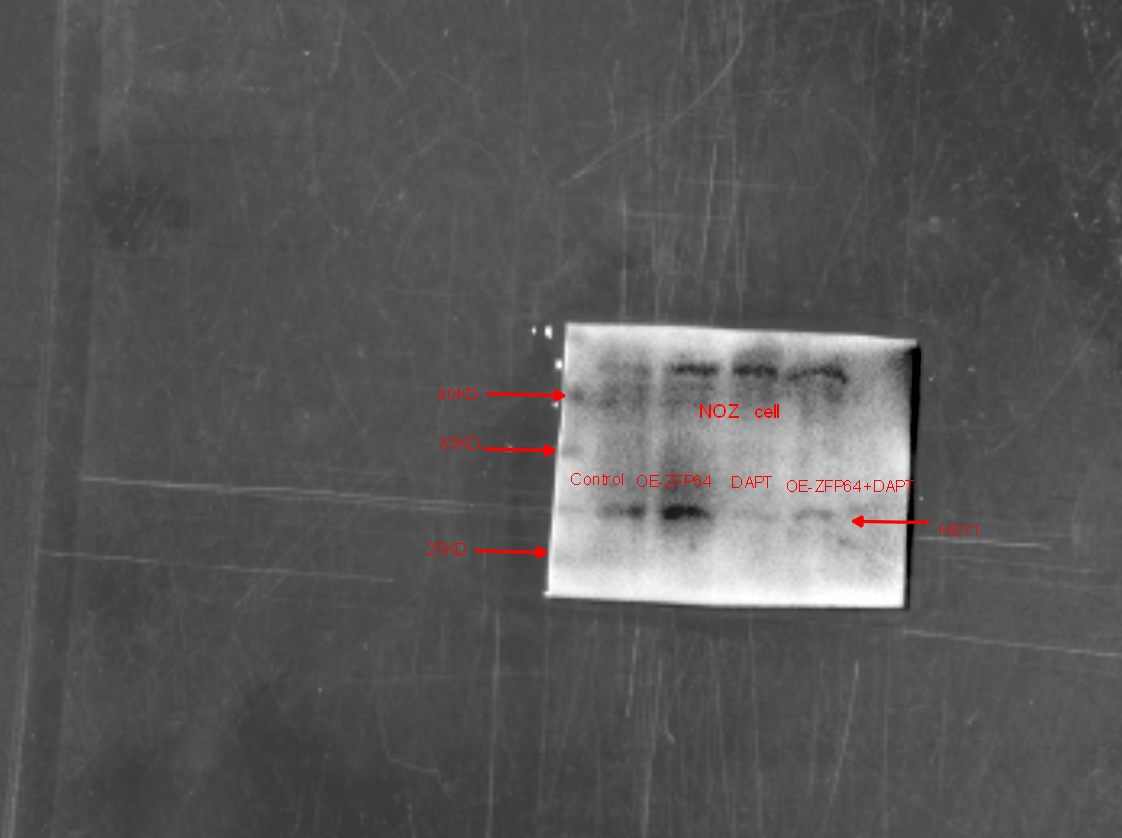

Supplement: Supplementary file 1 [file cancers-15-04508-s001.zip › cancers-2573702-supplementary/Figure S6-Figure 4A/HEY1 (NOZ).tif]

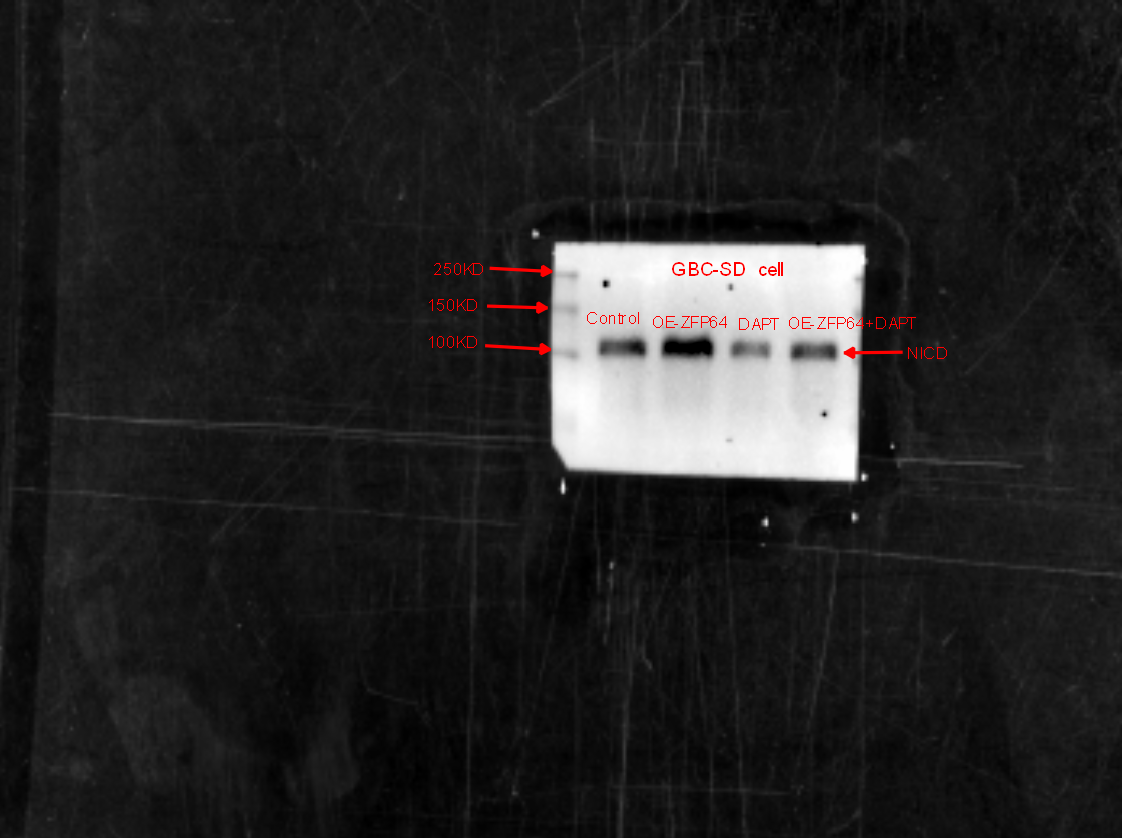

Supplement: Supplementary file 1 [file cancers-15-04508-s001.zip › cancers-2573702-supplementary/Figure S6-Figure 4A/NICD (GBC-SD).tif]

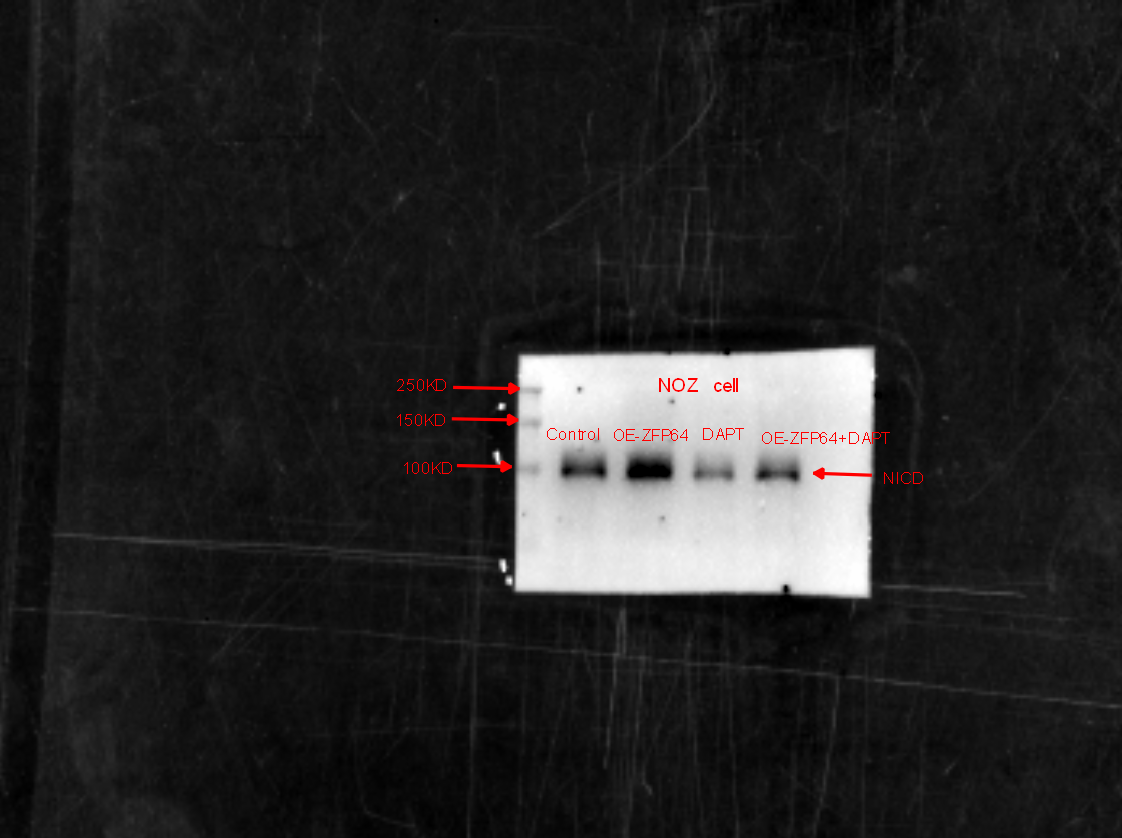

Supplement: Supplementary file 1 [file cancers-15-04508-s001.zip › cancers-2573702-supplementary/Figure S6-Figure 4A/NICD (NOZ).tif]

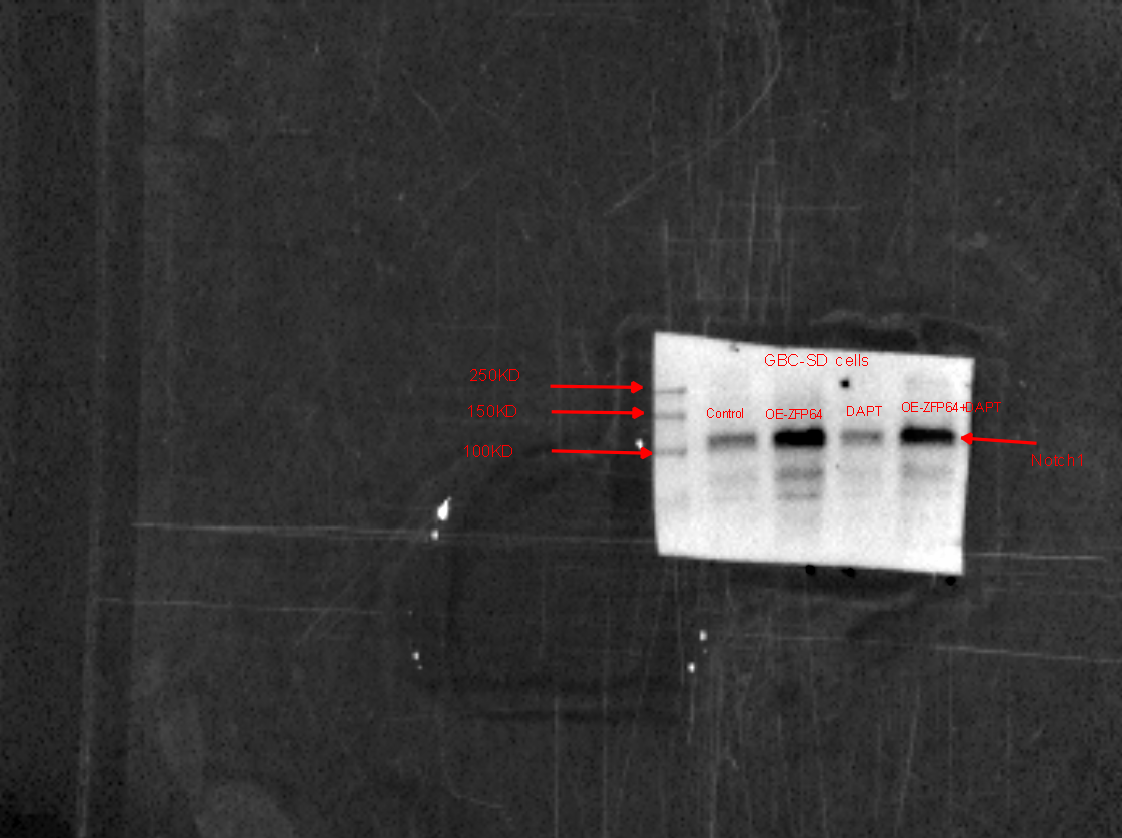

Supplement: Supplementary file 1 [file cancers-15-04508-s001.zip › cancers-2573702-supplementary/Figure S6-Figure 4A/Notch1 (GBC-SD).tif]

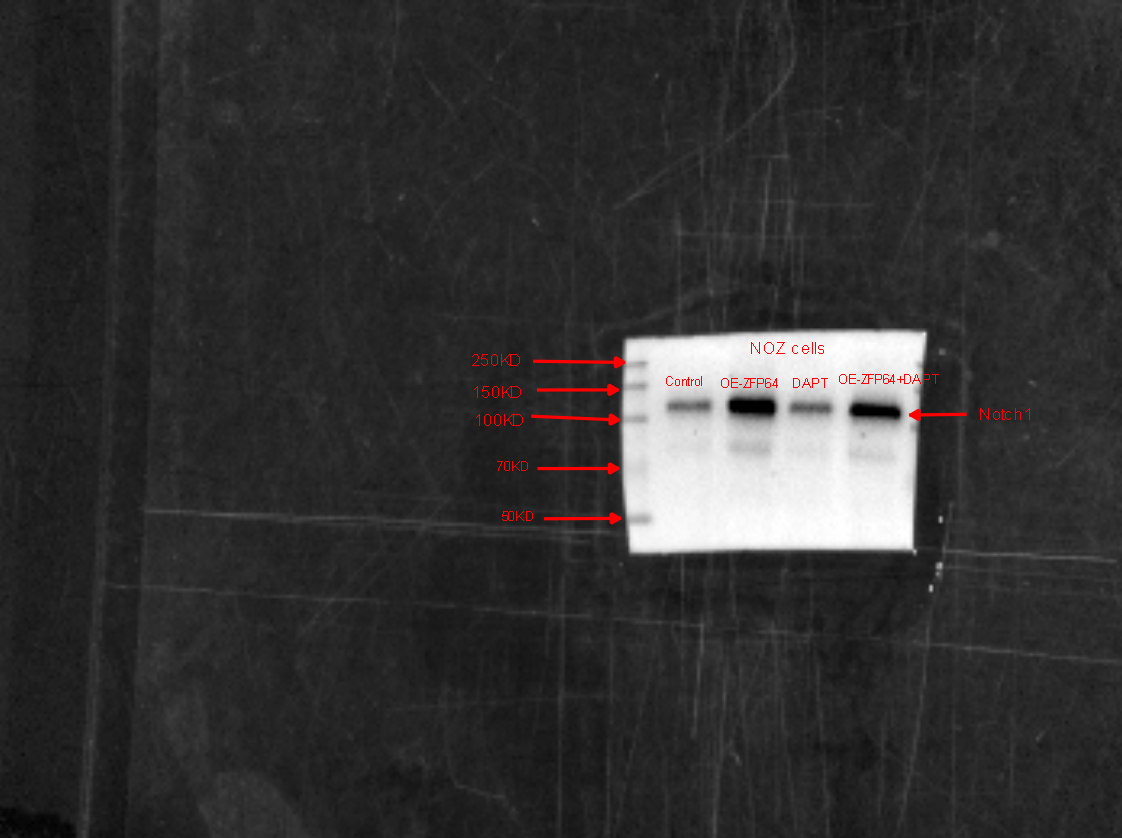

Supplement: Supplementary file 1 [file cancers-15-04508-s001.zip › cancers-2573702-supplementary/Figure S6-Figure 4A/Notch1 (NOZ).tif]

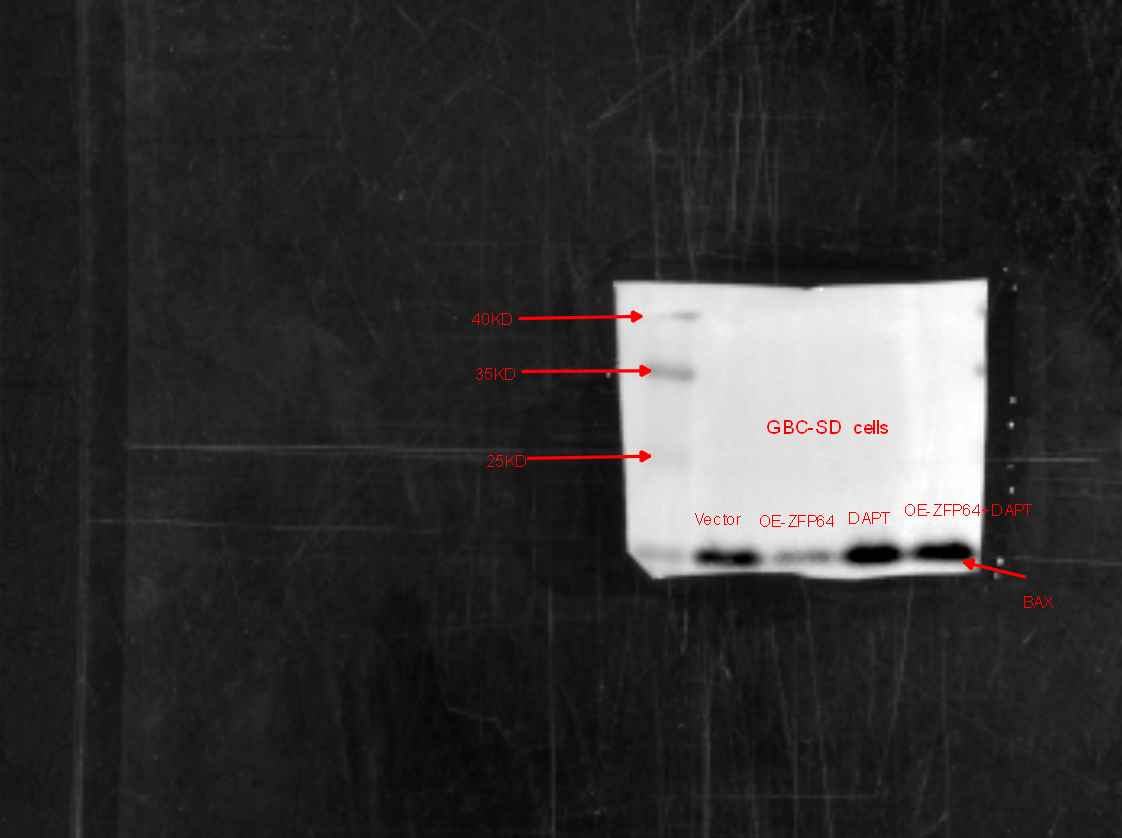

Supplement: Supplementary file 1 [file cancers-15-04508-s001.zip › cancers-2573702-supplementary/Figure S7-Figure 4C/BAX (GBC-SD).tif]

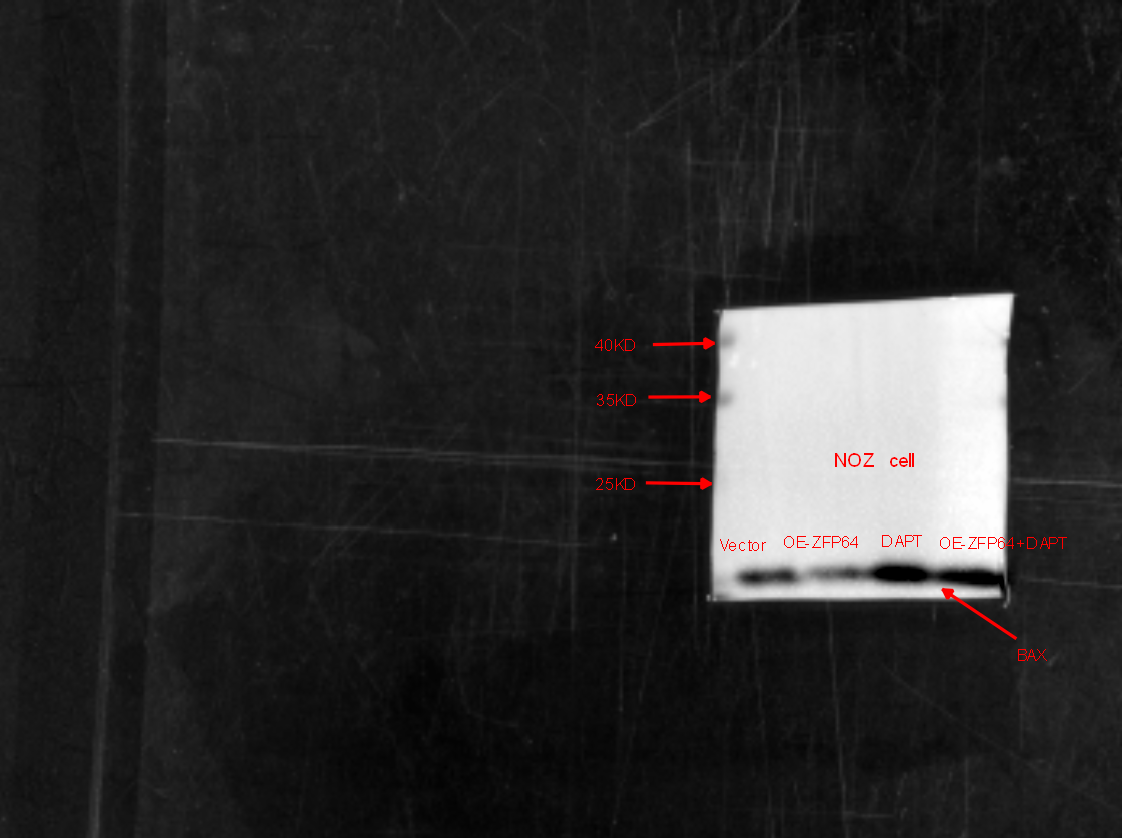

Supplement: Supplementary file 1 [file cancers-15-04508-s001.zip › cancers-2573702-supplementary/Figure S7-Figure 4C/BAX (NOZ).tif]

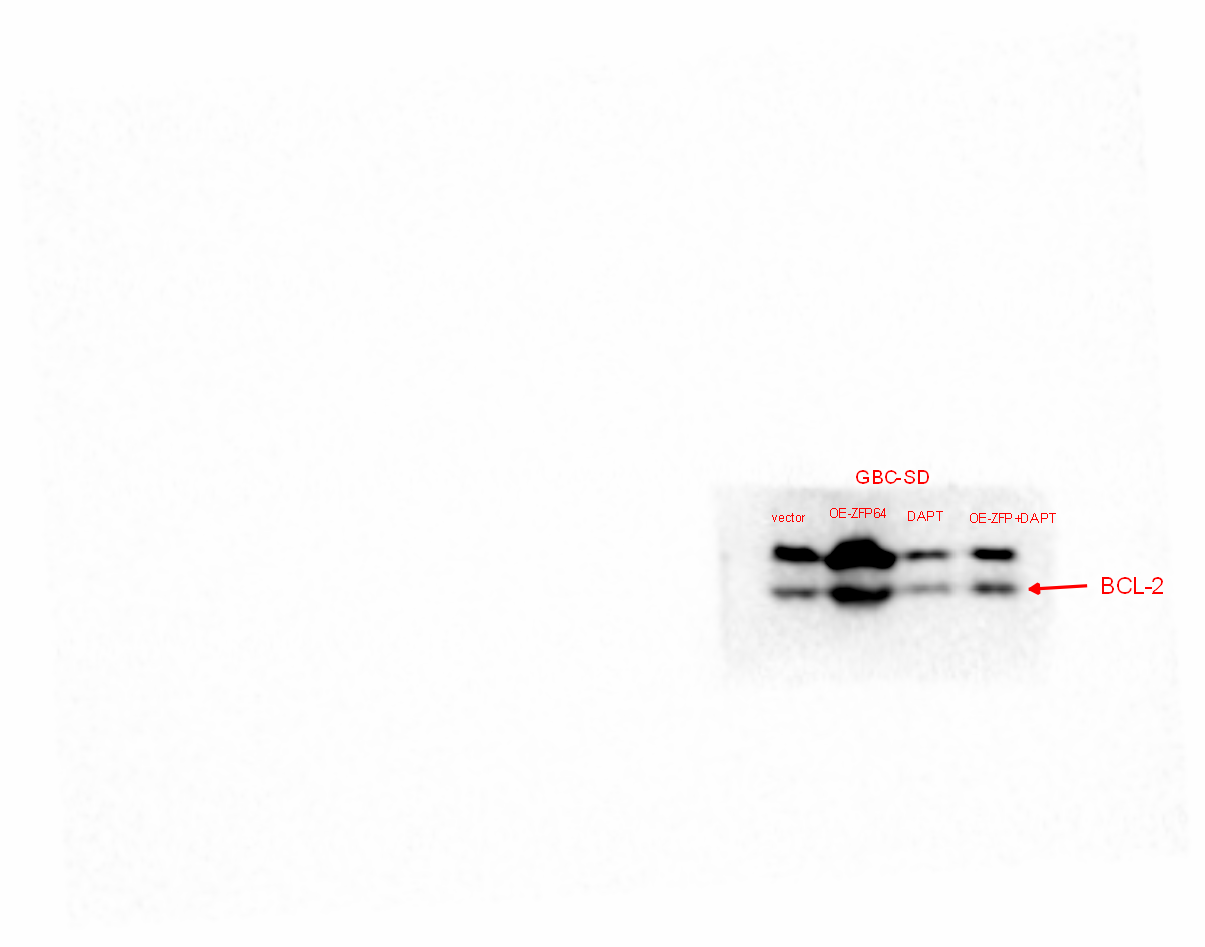

Supplement: Supplementary file 1 [file cancers-15-04508-s001.zip › cancers-2573702-supplementary/Figure S7-Figure 4C/BCL-2 (GBC-SD).tif]

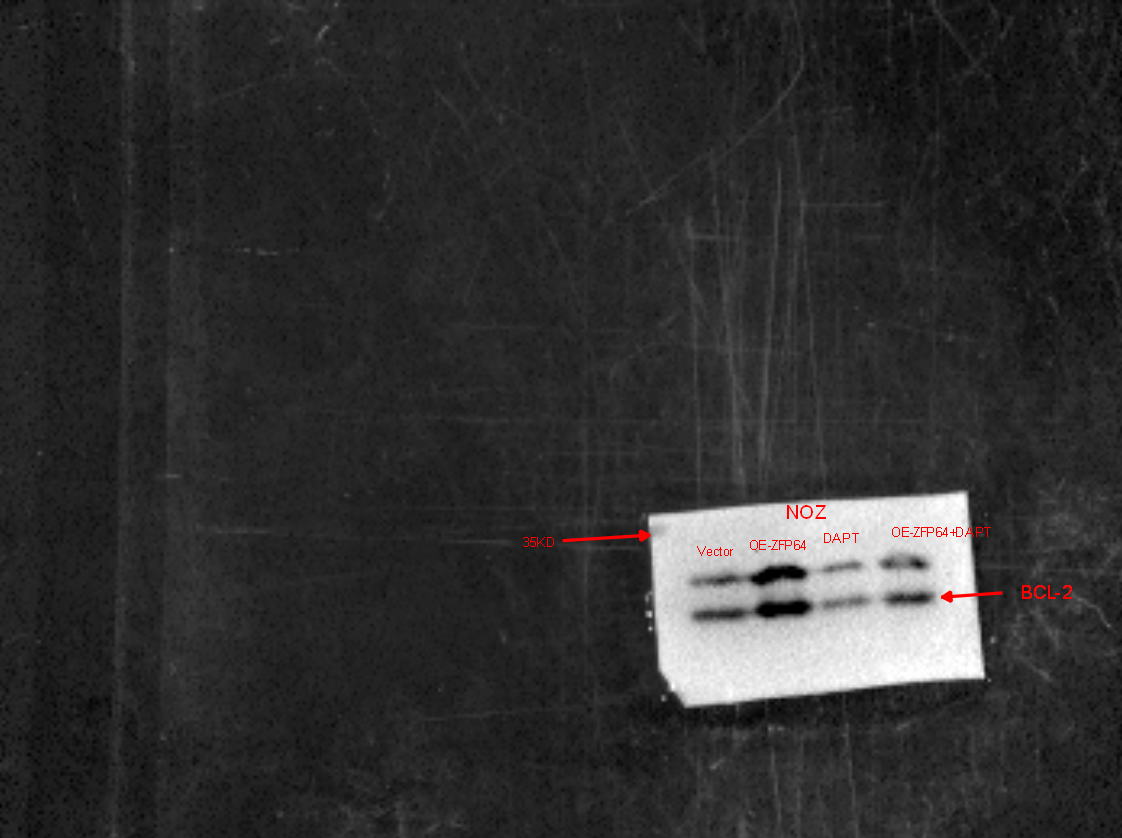

Supplement: Supplementary file 1 [file cancers-15-04508-s001.zip › cancers-2573702-supplementary/Figure S7-Figure 4C/BCL-2 (NOZ).tif]

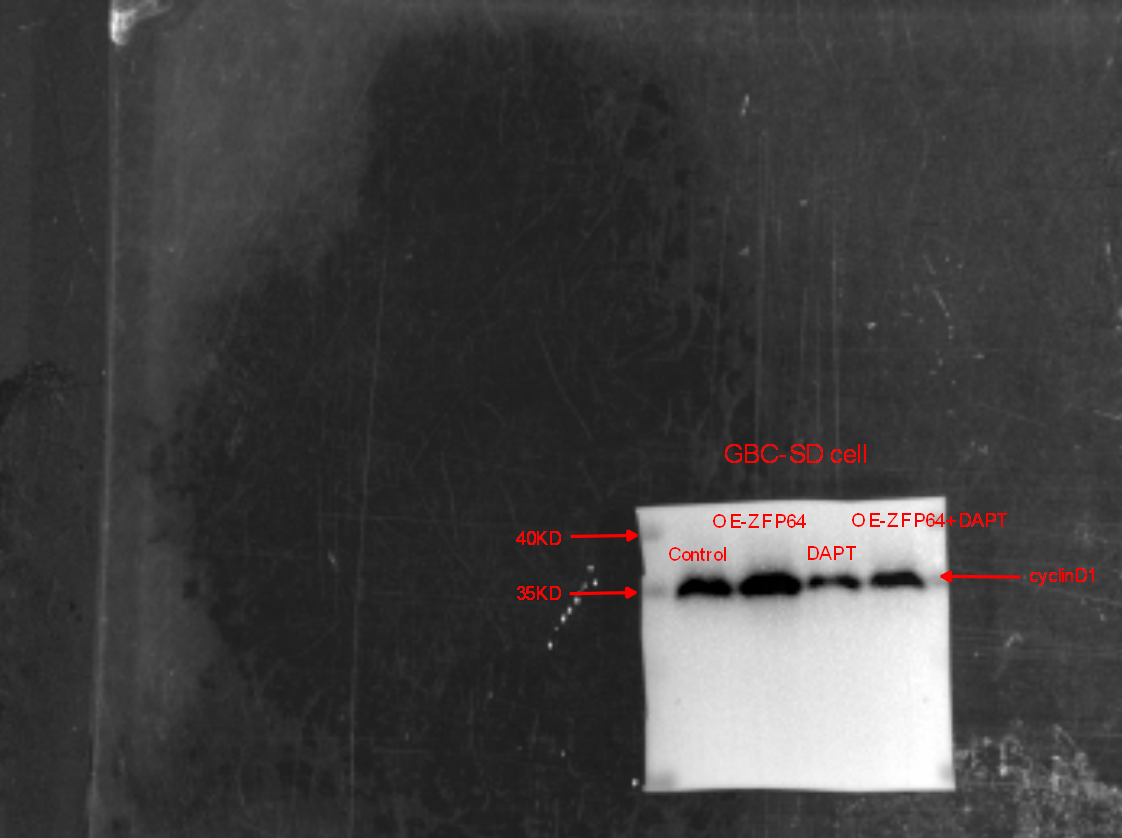

Supplement: Supplementary file 1 [file cancers-15-04508-s001.zip › cancers-2573702-supplementary/Figure S7-Figure 4C/cyclinD1 (GBC-SD).tif]

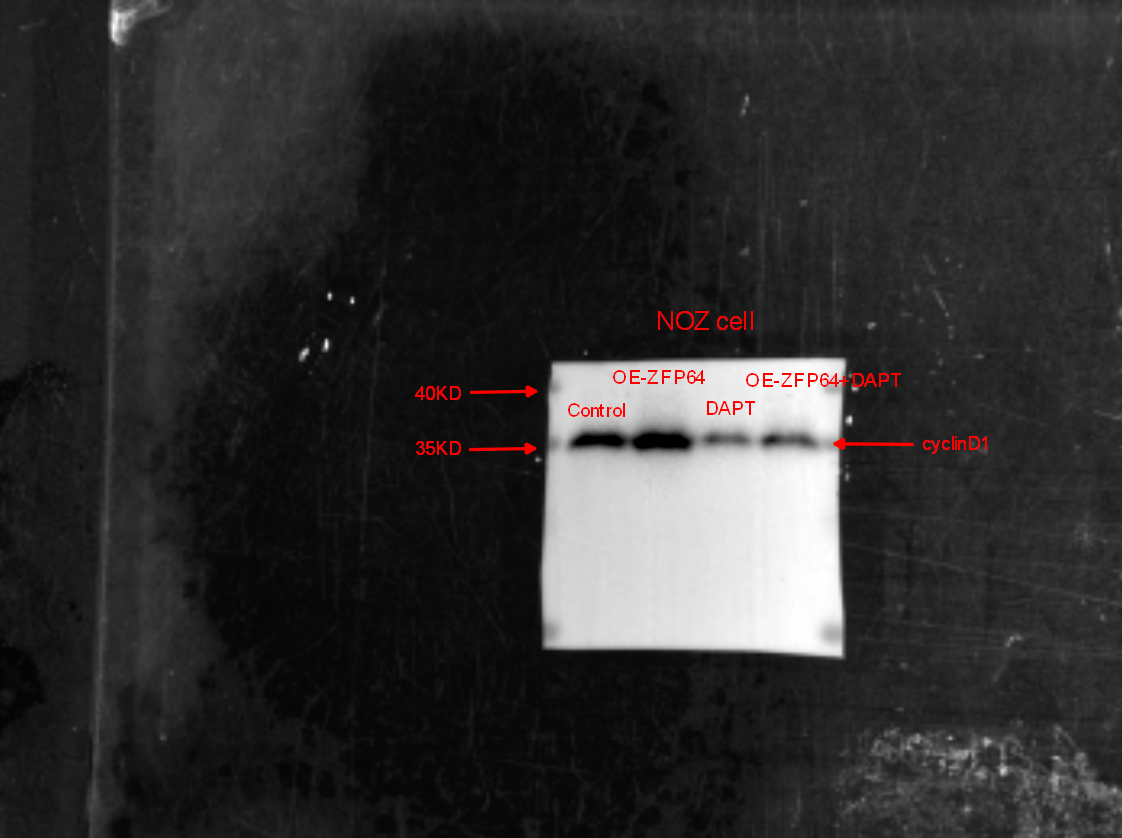

Supplement: Supplementary file 1 [file cancers-15-04508-s001.zip › cancers-2573702-supplementary/Figure S7-Figure 4C/cyclinD1 (NOZ).tif]

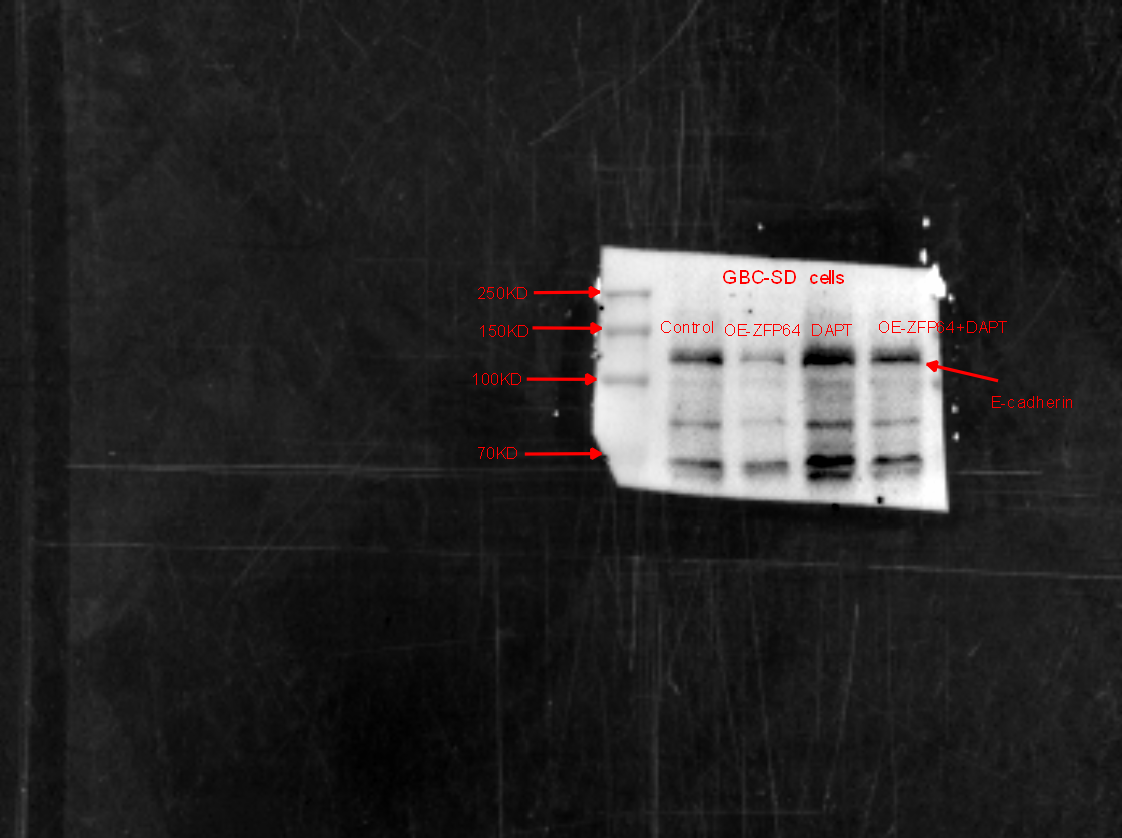

Supplement: Supplementary file 1 [file cancers-15-04508-s001.zip › cancers-2573702-supplementary/Figure S7-Figure 4C/E-cadherin (GBC-SD).tif]

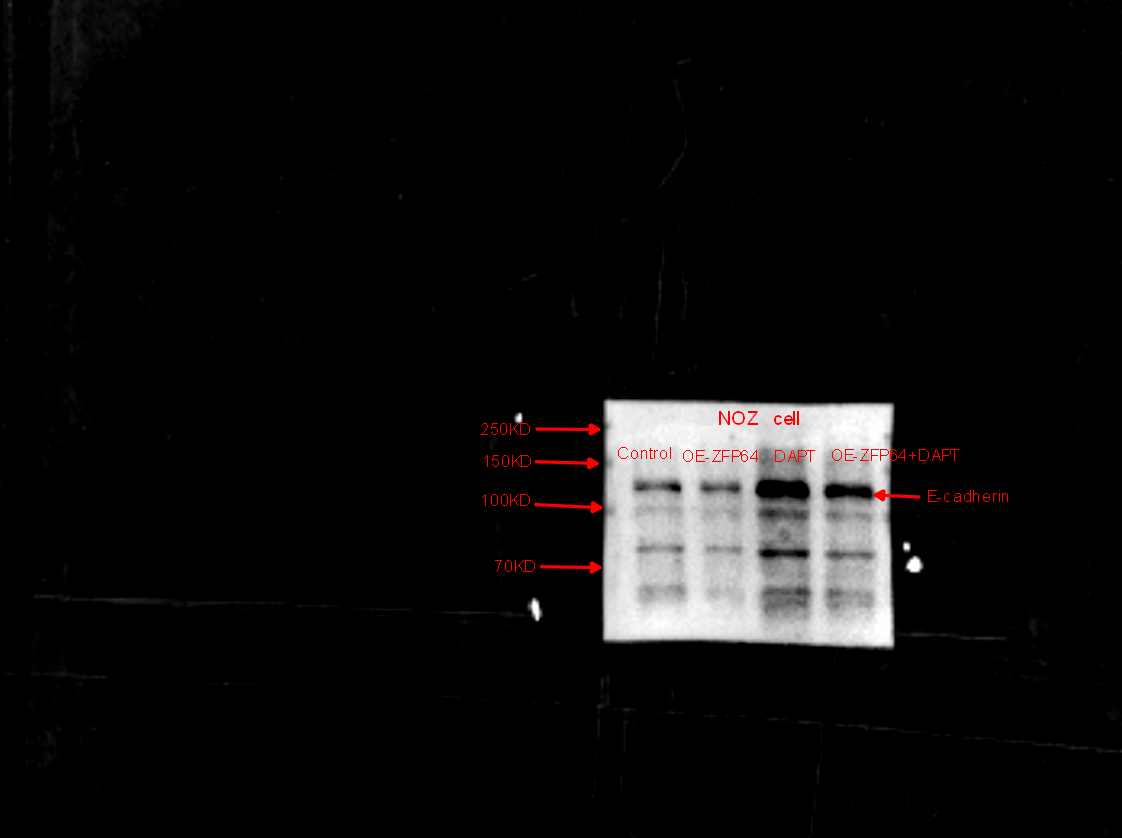

Supplement: Supplementary file 1 [file cancers-15-04508-s001.zip › cancers-2573702-supplementary/Figure S7-Figure 4C/E-cadherin (NOZ).tif]

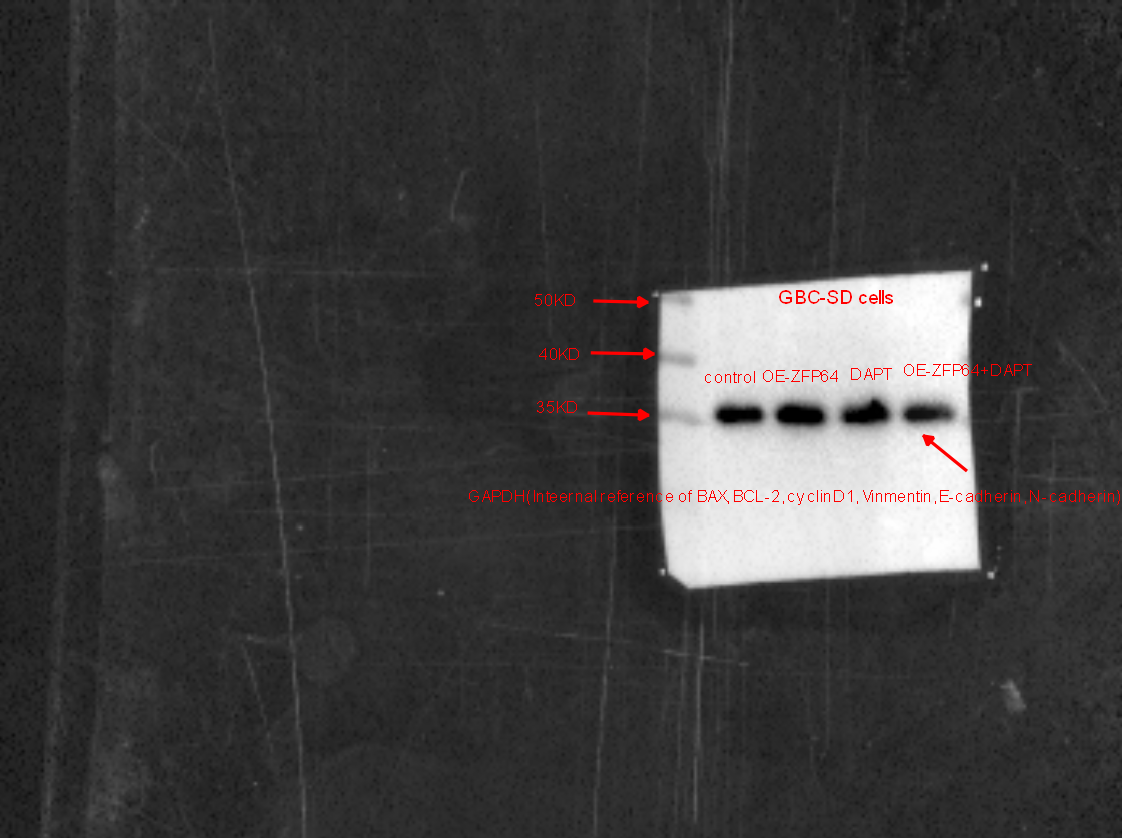

Supplement: Supplementary file 1 [file cancers-15-04508-s001.zip › cancers-2573702-supplementary/Figure S7-Figure 4C/GAPDH (GBC-SD).tif]

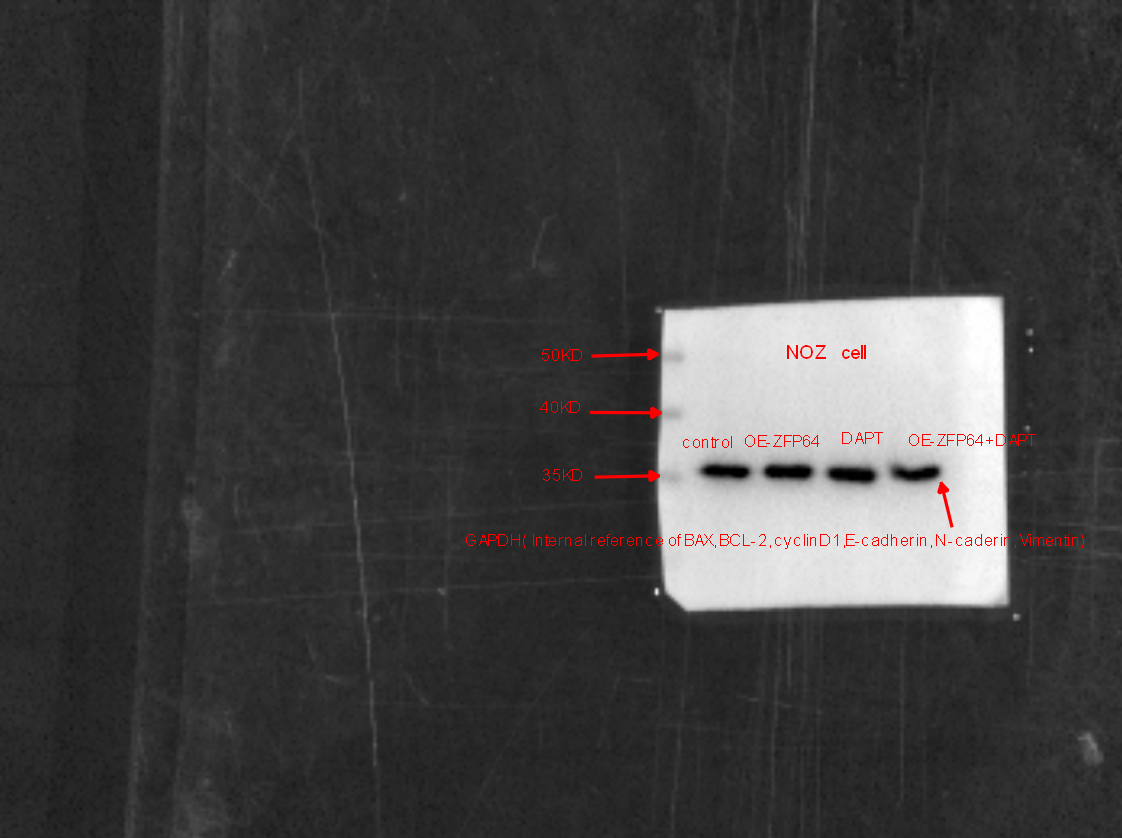

Supplement: Supplementary file 1 [file cancers-15-04508-s001.zip › cancers-2573702-supplementary/Figure S7-Figure 4C/GAPDH (NOZ).tif]

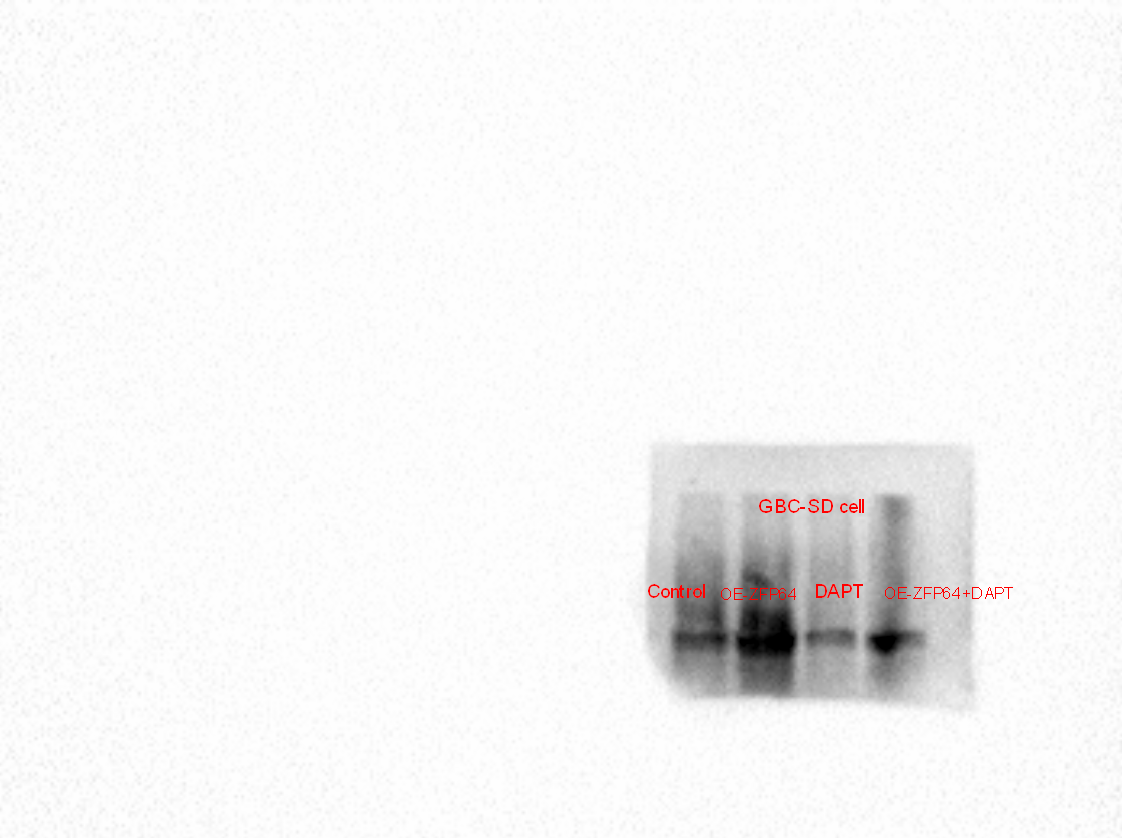

Supplement: Supplementary file 1 [file cancers-15-04508-s001.zip › cancers-2573702-supplementary/Figure S7-Figure 4C/N-cadherin (GBC-SD).tif]

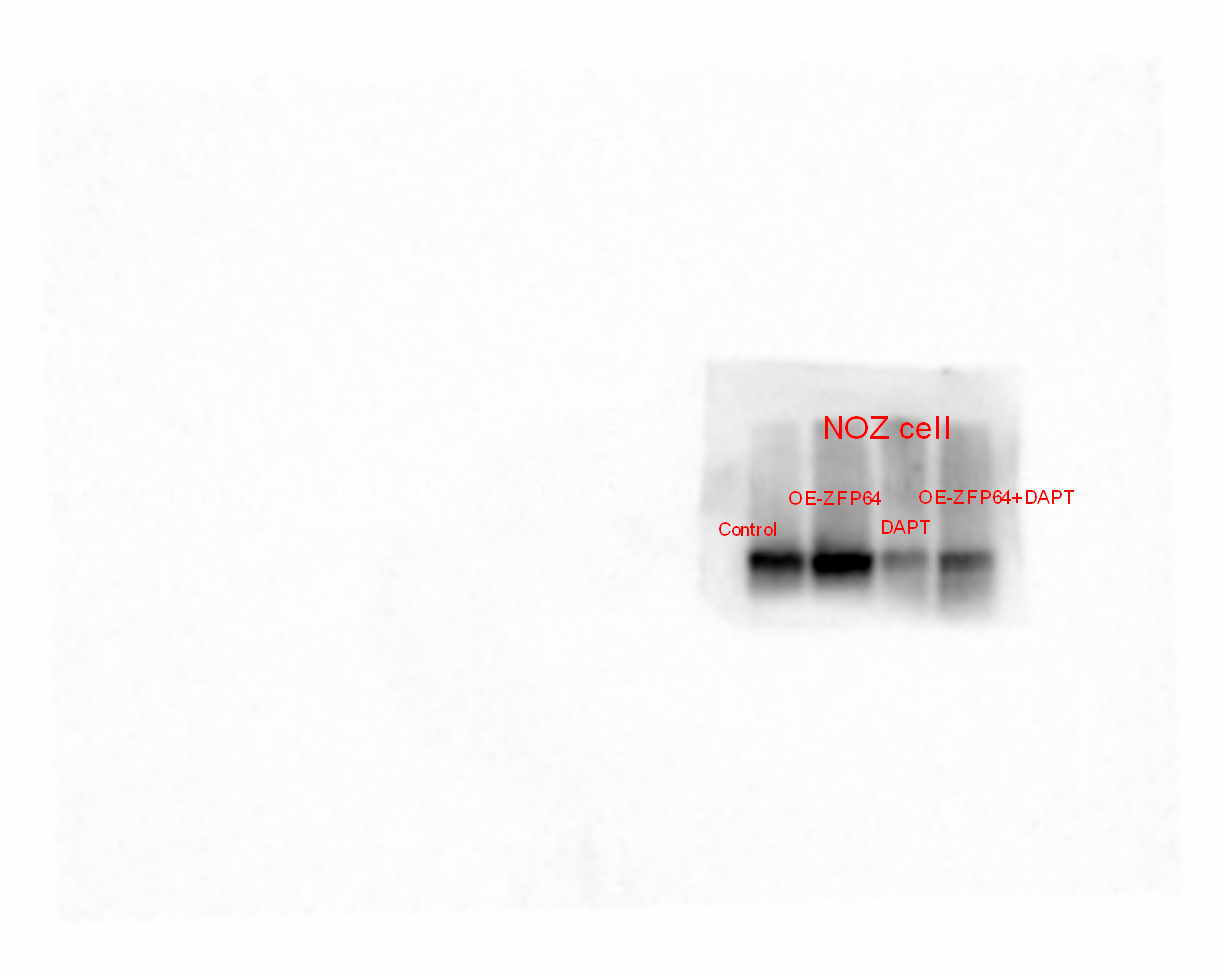

Supplement: Supplementary file 1 [file cancers-15-04508-s001.zip › cancers-2573702-supplementary/Figure S7-Figure 4C/N-cadherin (NOZ).tif]

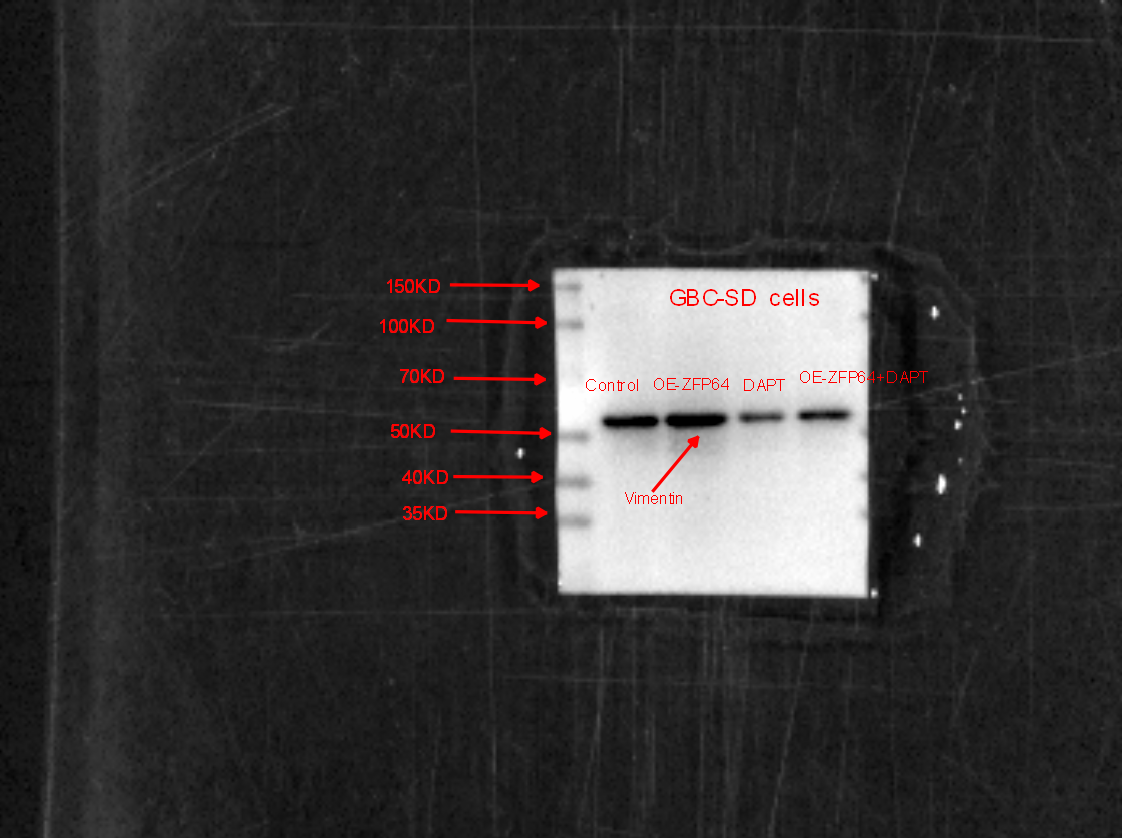

Supplement: Supplementary file 1 [file cancers-15-04508-s001.zip › cancers-2573702-supplementary/Figure S7-Figure 4C/Vimentin (GBC-SD).tif]

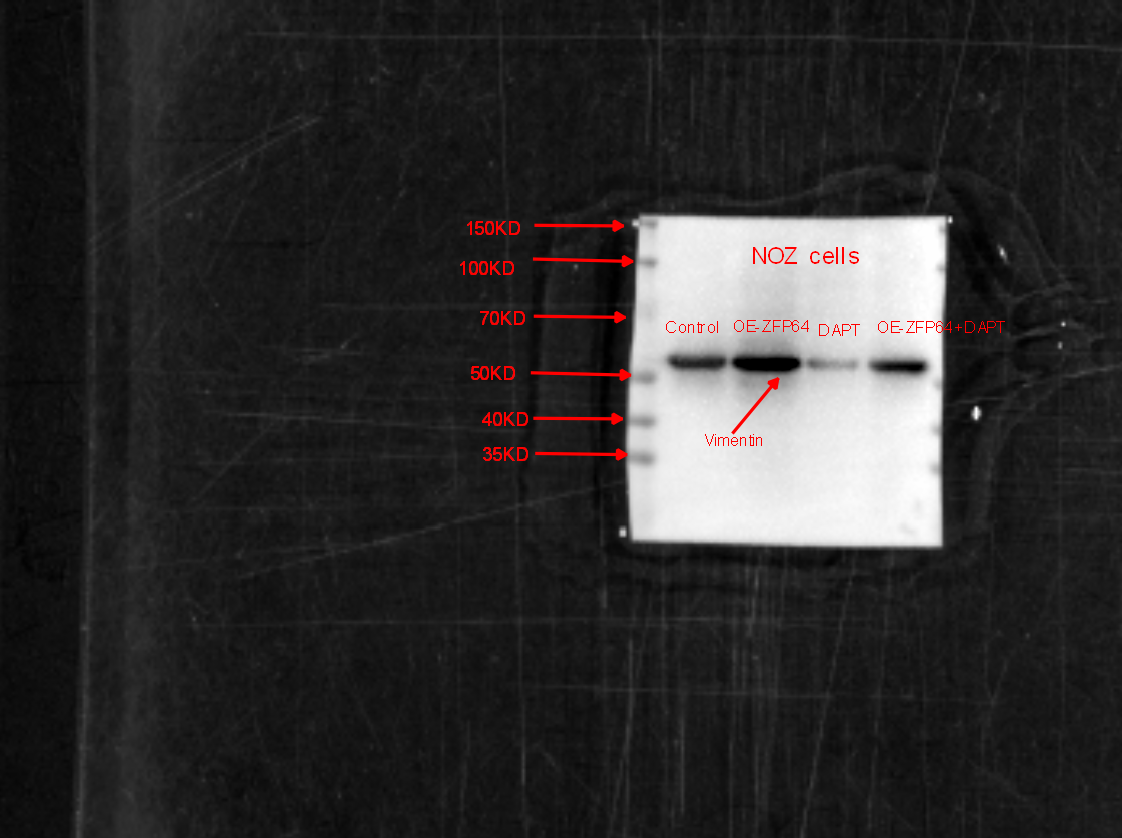

Supplement: Supplementary file 1 [file cancers-15-04508-s001.zip › cancers-2573702-supplementary/Figure S7-Figure 4C/Vimentin (NOZ).tif]

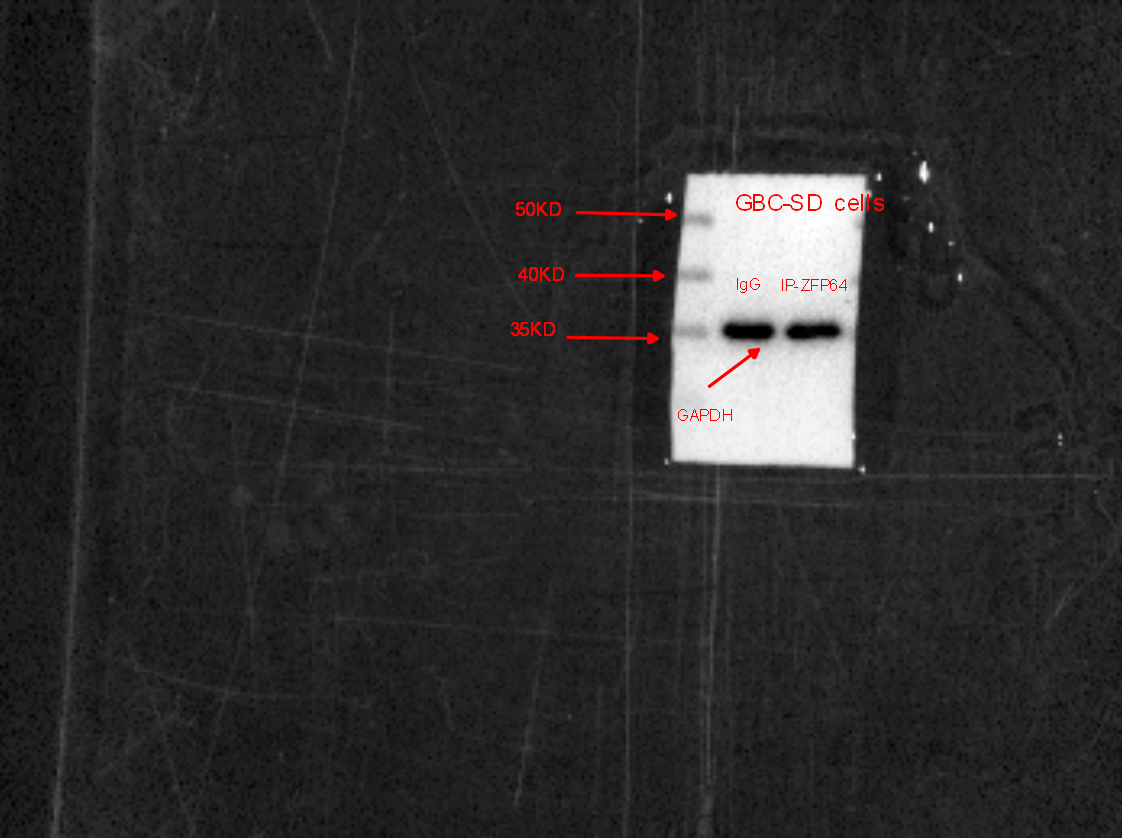

Supplement: Supplementary file 1 [file cancers-15-04508-s001.zip › cancers-2573702-supplementary/Figure S8-Figure 5F/GAPDH (GBC-SD).tif]

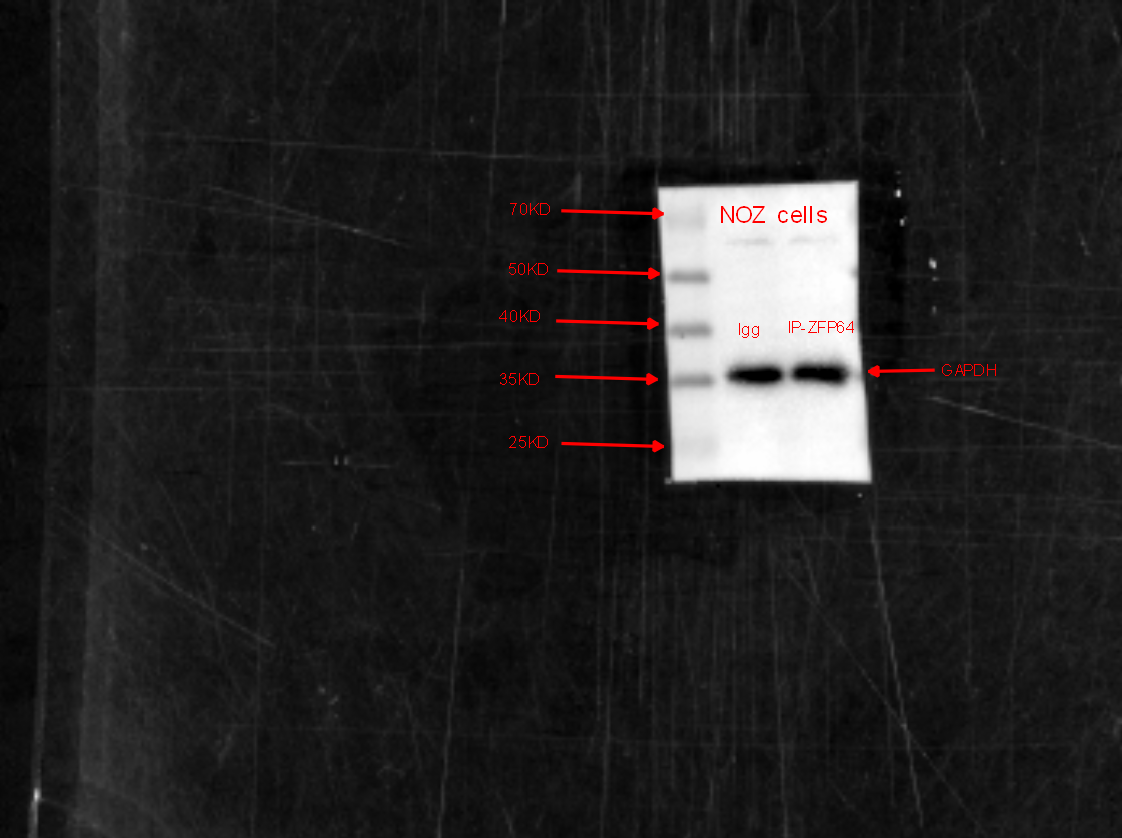

Supplement: Supplementary file 1 [file cancers-15-04508-s001.zip › cancers-2573702-supplementary/Figure S8-Figure 5F/GAPDH (NOZ).tif]

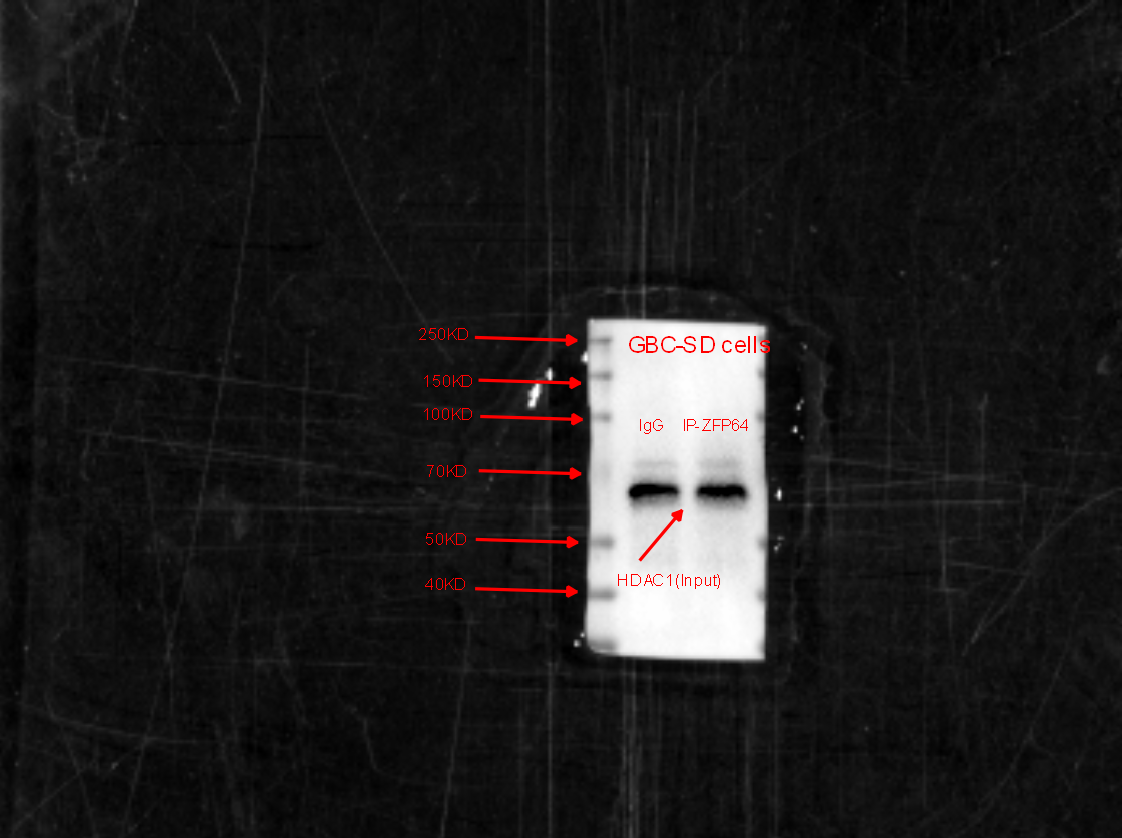

Supplement: Supplementary file 1 [file cancers-15-04508-s001.zip › cancers-2573702-supplementary/Figure S8-Figure 5F/HDAC1 (GBC-SD) (2).tif]

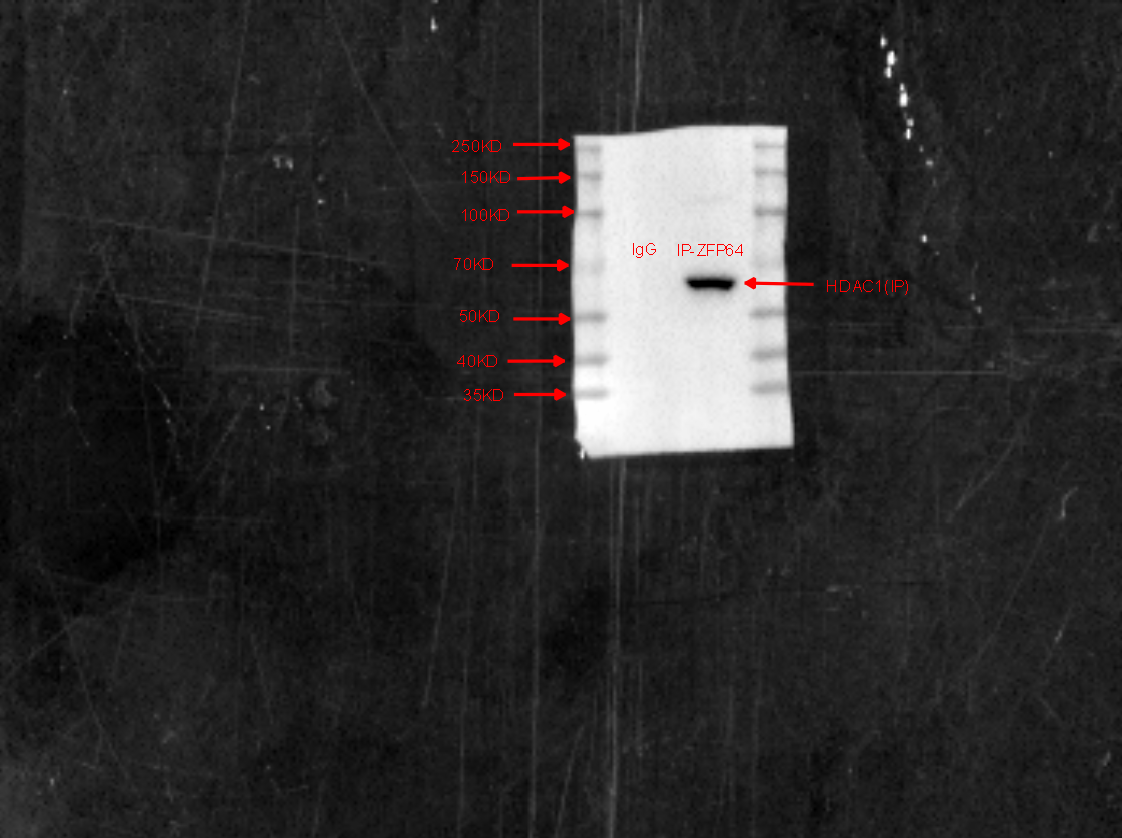

Supplement: Supplementary file 1 [file cancers-15-04508-s001.zip › cancers-2573702-supplementary/Figure S8-Figure 5F/HDAC1 (GBC-SD).tif]

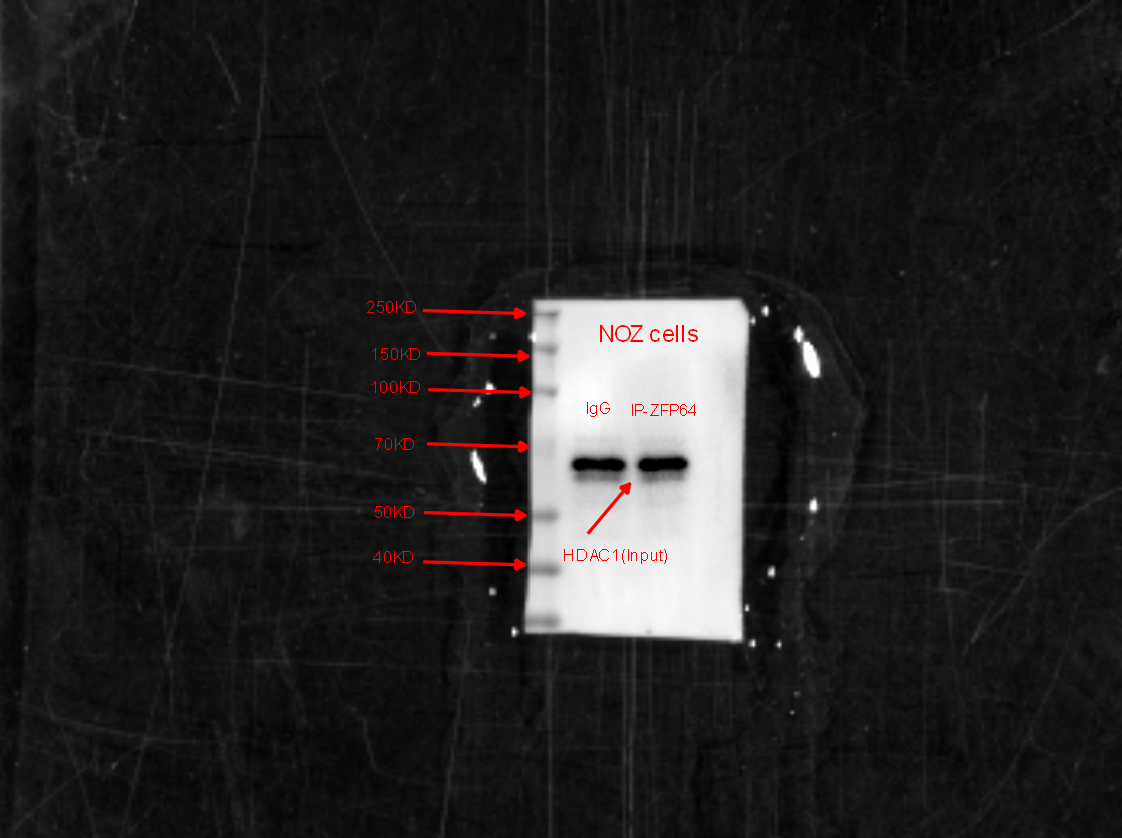

Supplement: Supplementary file 1 [file cancers-15-04508-s001.zip › cancers-2573702-supplementary/Figure S8-Figure 5F/HDAC1 (NOZ) (2).tif]
